# Supplementary material for: Pan-cancer analysis reveals cooperativity of both strands of microRNA that regulate tumorigenesis and patient survival
Source: Nat Commun. 2020 Feb 20;11:968. doi: 10.1038/s41467-020-14713-2 (PMC7033124; doi:10.1038/s41467-020-14713-2)
Supplement: Supplementary file 1 — Supplementary Information [file 41467_2020_14713_MOESM1_ESM.pdf]

Pan-cancer analysis reveals cooperativity of both strands of microRNA that regulate tumorigenesis and patient survival

Mitra et al.

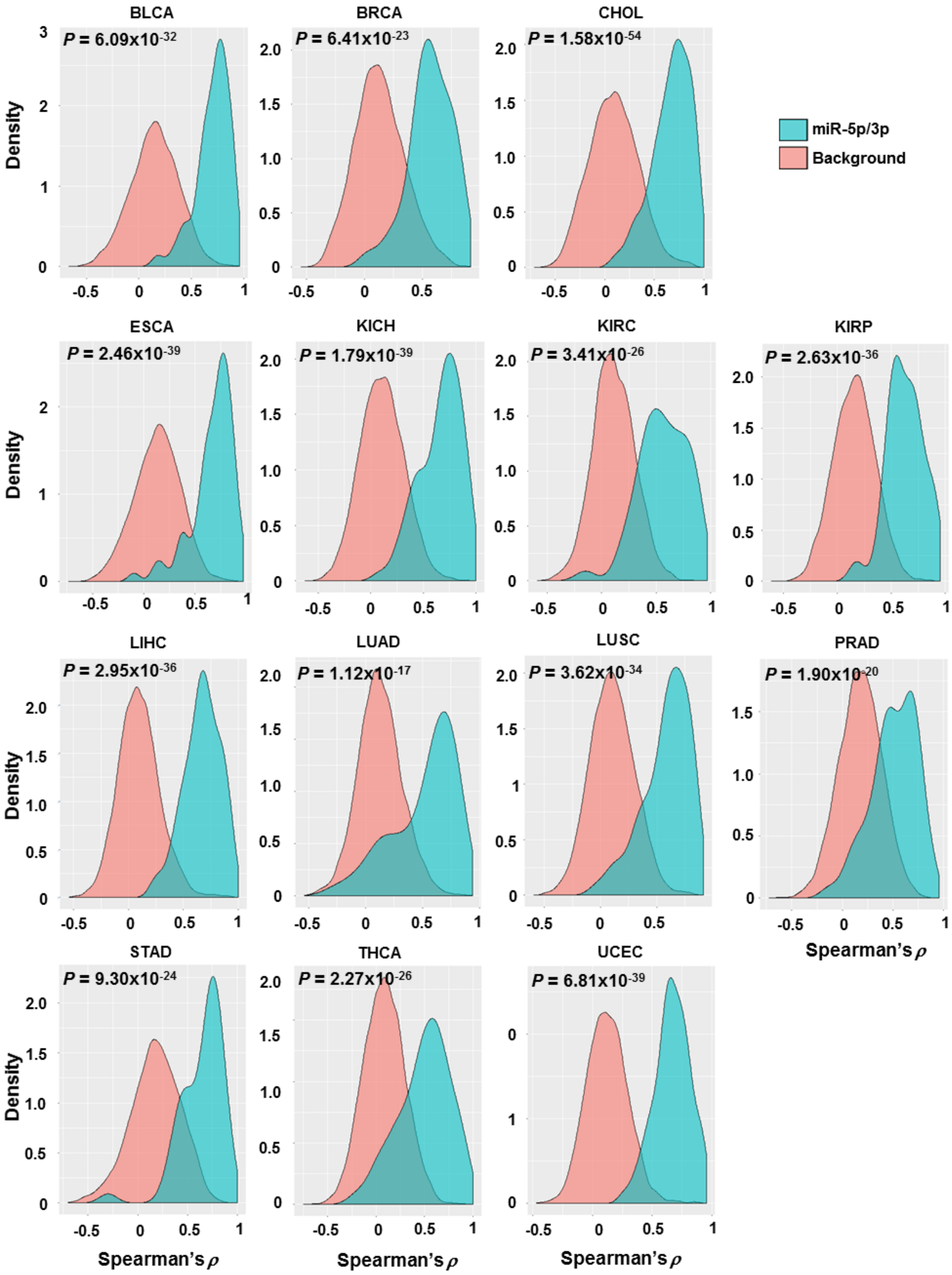

Supplementary Figure 1. Concordance in miRNA-5p/3p expression correlations across cancer types. Density plots showing distribution of miRNA-5p/3p expression correlations (green) compared to the background miRNA pairs (red) that selected randomly from different precursor miRNA. Fourteen different cancer types from TCGA were analyzed. X-axis is expression correlations measured by Spearman's rank correlation. *P*-values determined by Wilcoxon rank-sum test (two-tailed).

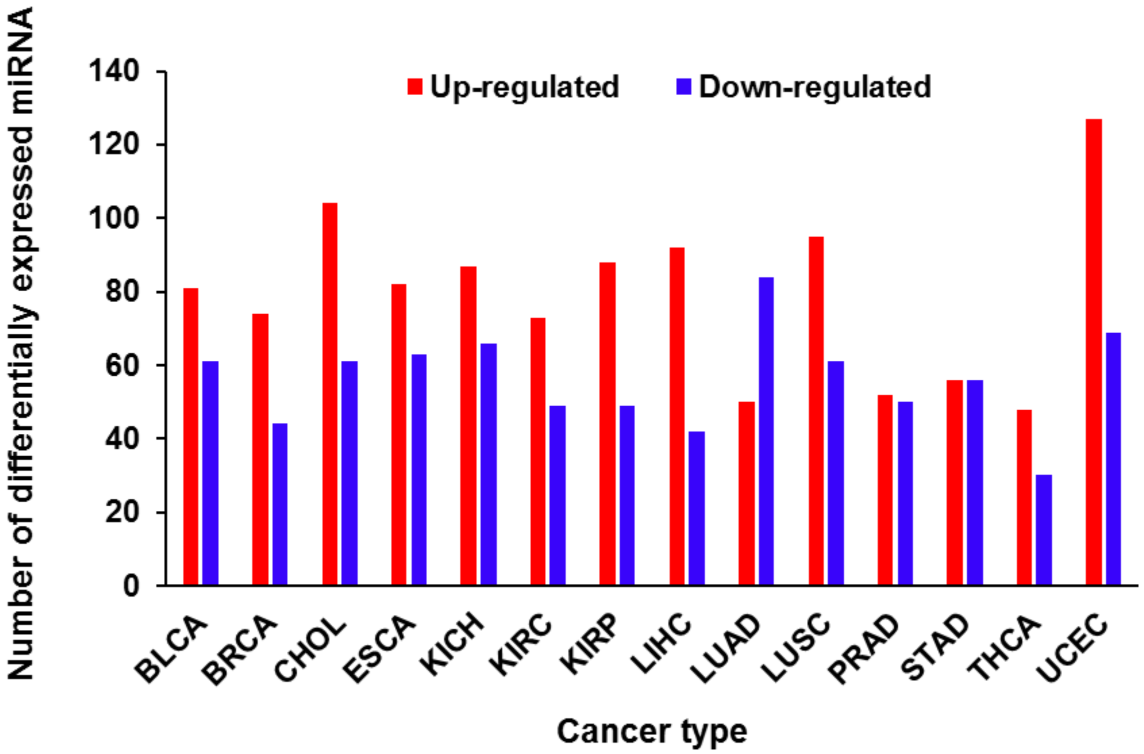

Supplementary Figure 2. Number of differentially expressed miRNA determined in each of the 14 TCGA cancer types. Up- or down-regulation was measured in cancer samples compared with corresponding normal tissue using the tool edgeR. The miRNA with at least 1.5 fold-change and adjusted $P<0.05$, adjusted by Benjamini-Hochberg[1] method, were determined as differentially expressed.

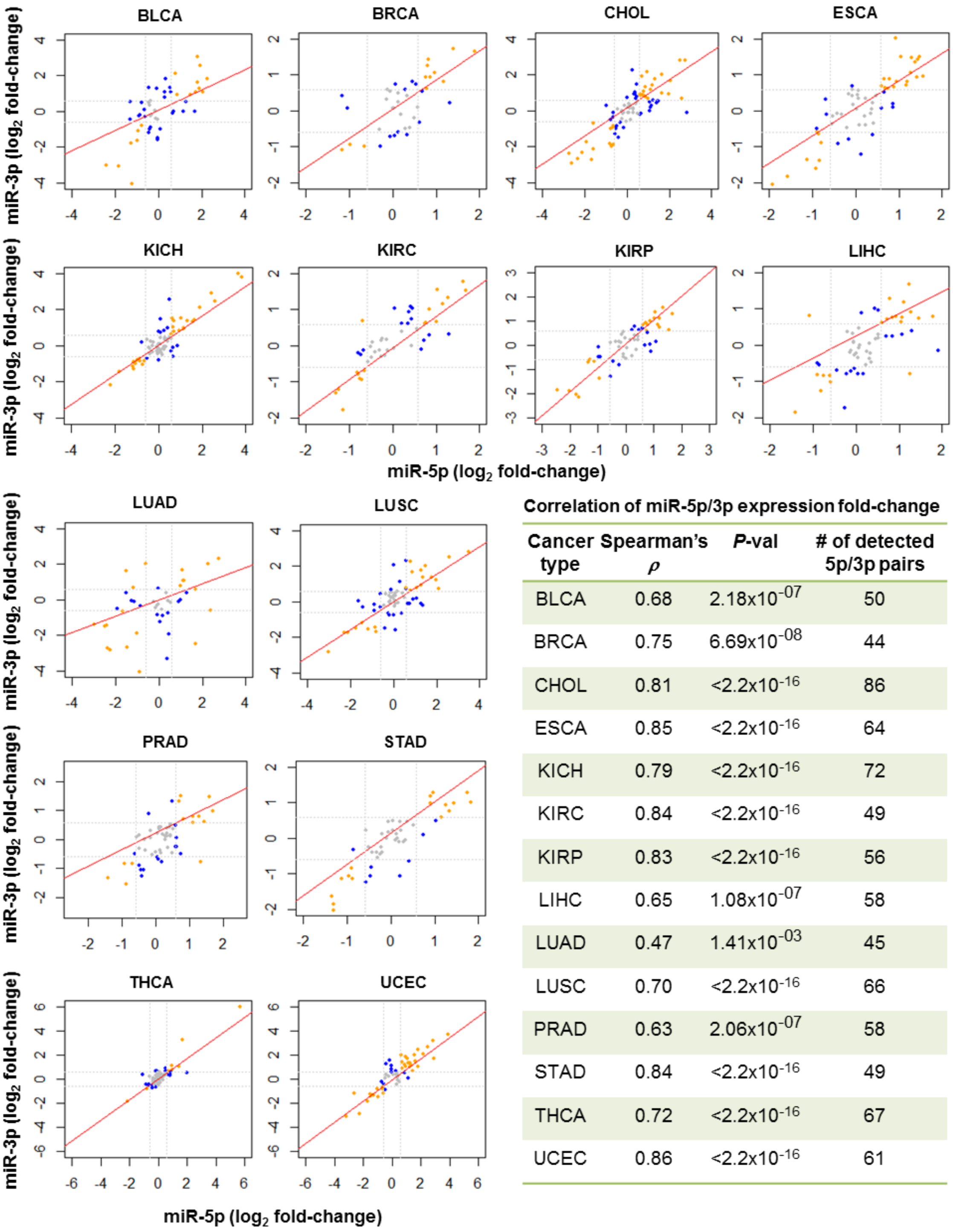

Correlation of miR-5p/3p expression fold-change

Cancer type	Spearman's ρ	*P*-val	# of detected 5p/3p pairs
BLCA	0.68	2.18×10^{-07}	50
BRCA	0.75	6.69×10^{-08}	44
CHOL	0.81	$<2.2 \times 10^{-16}$	86
ESCA	0.85	$<2.2 \times 10^{-16}$	64
KICH	0.79	$<2.2 \times 10^{-16}$	72
KIRC	0.84	$<2.2 \times 10^{-16}$	49
KIRP	0.83	$<2.2 \times 10^{-16}$	56
LIHC	0.65	1.08×10^{-07}	58
LUAD	0.47	1.41×10^{-03}	45
LUSC	0.70	$<2.2 \times 10^{-16}$	66
PRAD	0.63	2.06×10^{-07}	58
STAD	0.84	$<2.2 \times 10^{-16}$	49
THCA	0.72	$<2.2 \times 10^{-16}$	67
UCEC	0.86	$<2.2 \times 10^{-16}$	61

Supplementary Figure 3. Concordant expression fold-change of miRNA-5p/3p pairs across cancer types. Scatterplots show expression fold-change distribution of miRNA-5p/3p pairs in 14 different cancer types compared with the corresponding normal samples. Each dot represents fold-change of one miRNA-5p/3p pair in one cancer type where orange, blue, and grey symbols indicate significant differential expression of both mature strands, one mature strand, or neither strand, respectively. X- and Y-axis indicates direction and degree of fold-change of miRNA-5p/3p pairs. Regression line indicates positive correlation. Table has Spearman's correlation score and *P*-values (two-tailed) indicating degree of concordance in expression fold-changes of miRNA-5p/3p pairs.

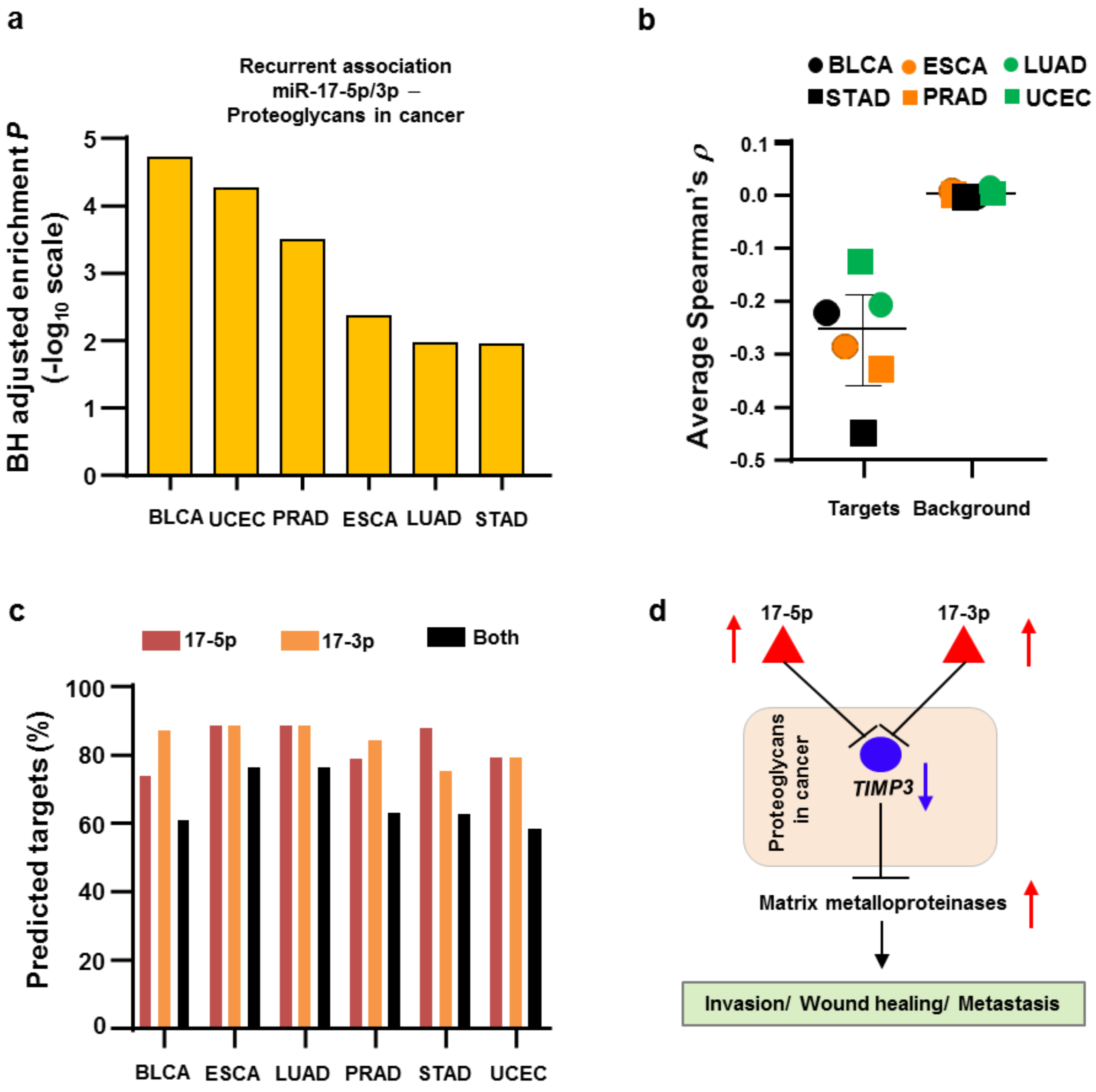

Supplementary Figure 4. miRNA-17-5p/3p-mediated suppression of pathway 'Proteoglycans in cancer' across cancer types. a) The pathway 'Proteoglycans in cancer' in the KEGG database is significantly enriched with predicted targets of the miR-17-5p/3p pair in the indicated cancer types (X-axis). Y-axis represents enrichment significance. **b**) Distribution of average expression correlation between the miR-17-5p/3p pair and the pathway 'Proteoglycans in cancer' in the indicated cancer type (*n*=6). The miRNA-pathway inverse association strength was compared against the expression correlations observed in randomly selected miRNA-gene pairs. Targets: denotes genes in the indicated pathway that were predicted to be regulated by miR-17-5p, miR-17-3p, or both in the specific cancer. Background: denotes randomly selected miRNA-gene pairs (see methods in the main text for details). Data are presented as median with interquartile range. **c**) Proportion of the predicted targets in the indicated pathway only regulated by 5p, 3p, or both strands of miR-17 in the indicated cancer type. **d**) A schematic illustrating both 5p and 3p strands of miR-17 directly regulate *TIMP3*, a member of the pathway 'Proteoglycans in cancer', and coordinately repress its expression. TIMP3 inhibits the expression of matrix metalloproteinases, which facilitate cancer cell migration and invasion[2-4]. Red indicates up- and blue indicates down-regulation in cancer compared to normal. Source data are provided as a Source Data file.

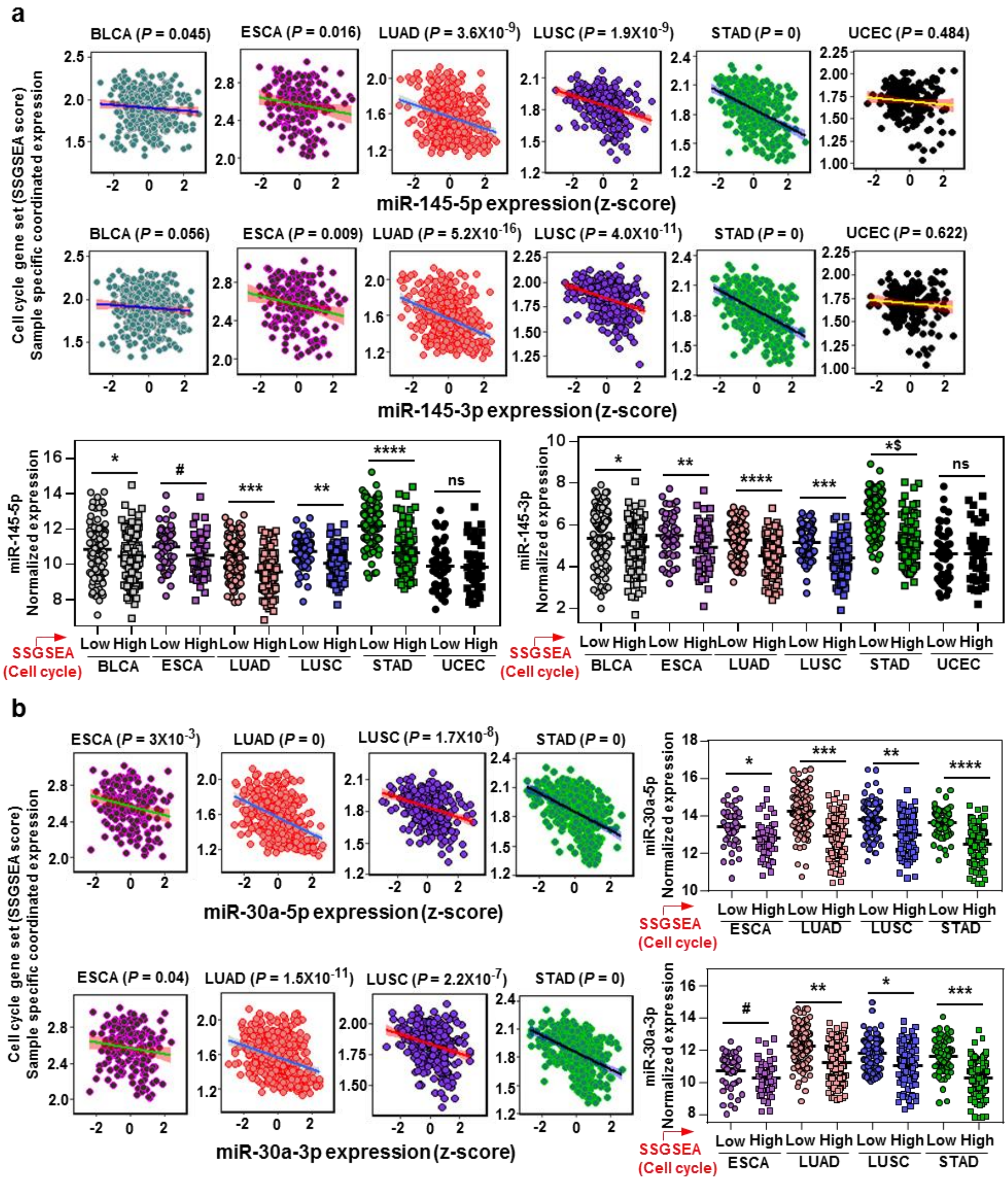

Supplementary Figure 5. Cancer-specific association between levels of cell cycle activation and miRNA expression. Sample-specific cell cycle pathway activation scores, measured by single-sample gene set enrichment analysis (SSGSEA), were plotted against expression levels of either miR-145-5p or miR-145-3p (**a**) and miR-30a-5p or miR-30a-3p (**b**). Regression lines and correlation *P* values indicate association direction and significance between miRNA and pathway activation levels across different cancer types (top in a and left in b). For each cancer type, samples were ranked according to SSGSEA score and stratified into four quartiles. Expression of an individual miRNA strand was measured in the samples with higher cell cycle activation scores (highest quartile) compared with the samples with lower cell cycle activation scores (lowest quartile) across the cancer types (bottom in a and right in b). For a (miR-145-5p), $^{\#}P$=0.064, **P*=0.048, ***P*=$1.25\text{x}10^{-7}$, ****P*=$9.09\text{x}10^{-8}$, *****P*=$1.15\text{x}10^{-14}$; for a (miR-145-3p), **P*=0.027, ***P*=0.022, ****P*=$2.87\text{x}10^{-9}$, *****P*=$2.09\text{x}10^{-11}$, $*^{\$}P<1\text{x}10^{-15}$; for b (miR-30a-5p), **P*=0.004, ***P*=$2.98\text{x}10^{-7}$, ****P*<$1\text{x}10^{-15}$, *****P*<$1\text{x}10^{-15}$; for b (miR-30a-3p), $^{\#}P$=0.076, **P*=$2.04\text{x}10^{-5}$, ***P*=$1.90\text{x}10^{-10}$, ****P*<$1\text{x}10^{-15}$; ns= not significant. Data are presented as mean values ± SEM. Source data are provided as a Source Data file.

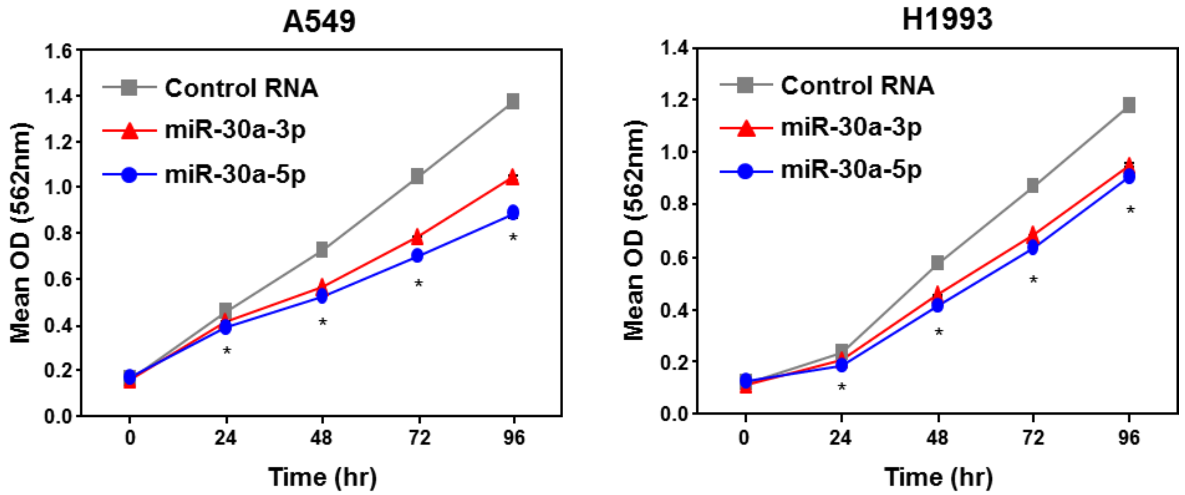

Supplementary Figure 6. Increased levels of miR-30a-5p or miR-30a-3p decrease lung adenocarcinoma cell growth. Lung cancer cell lines were transfected with 50nM miR-30a-5p mimic, miR-30a-3p mimic, or negative control RNA. MTT assays were performed at intervals in quadruplicate. Each assay was performed 2 independent times for both cell lines and one representative experiment is shown. For A549, $*P<3.70\times10^{-6}$ and for H1993, $*P<1.37\times10^{-3}$; two-tailed *t*-tests (comparing individual miRNA mimics to control RNA). Data are presented as mean values ± SEM; error bars are within the symbols.

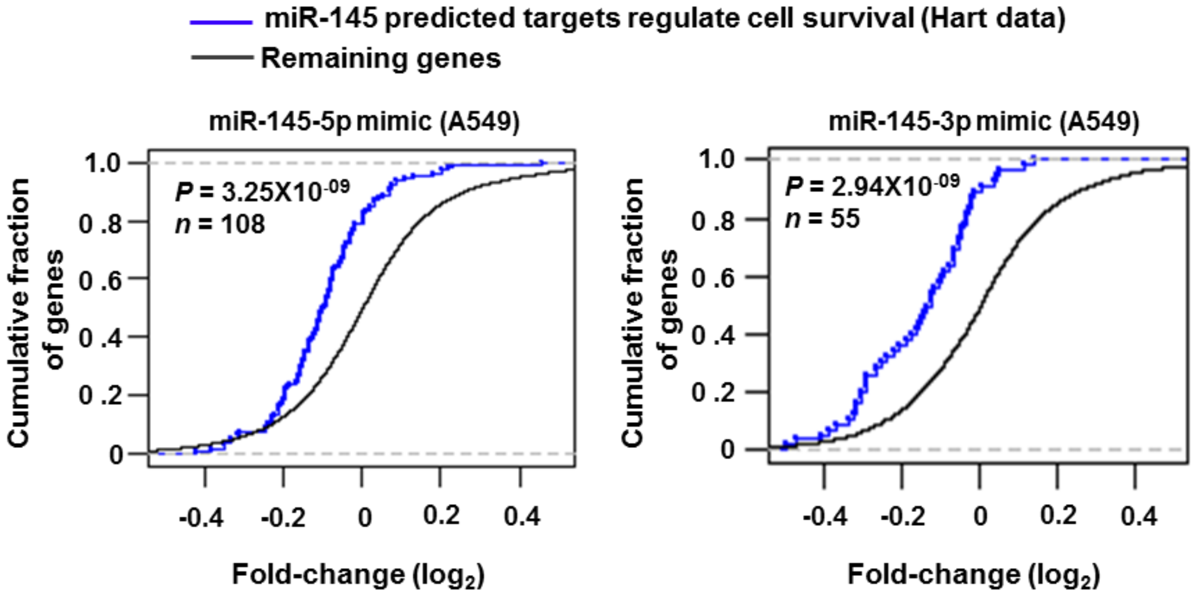

Supplementary Figure 7. Expression changes of essential genes that were predicted to be regulated by miR-145-5p or miR-145-3p. The miRNA-essential gene pairs were obtained from regression analysis in TCGA LUAD. The common essential gene list was downloaded from the Hart laboratory (http://hart-lab.org/downloads). *n* denotes the number of predicted target essential genes of the corresponding miRNA. The miRNA perturbation profile was obtained from GSE107008. *P*-value (two-tailed Kolmogorov-Smironov test) denotes significance in the depletion of the essential genes, which were predicted to be regulated by the indicated miRNA, compared with the remaining genes.

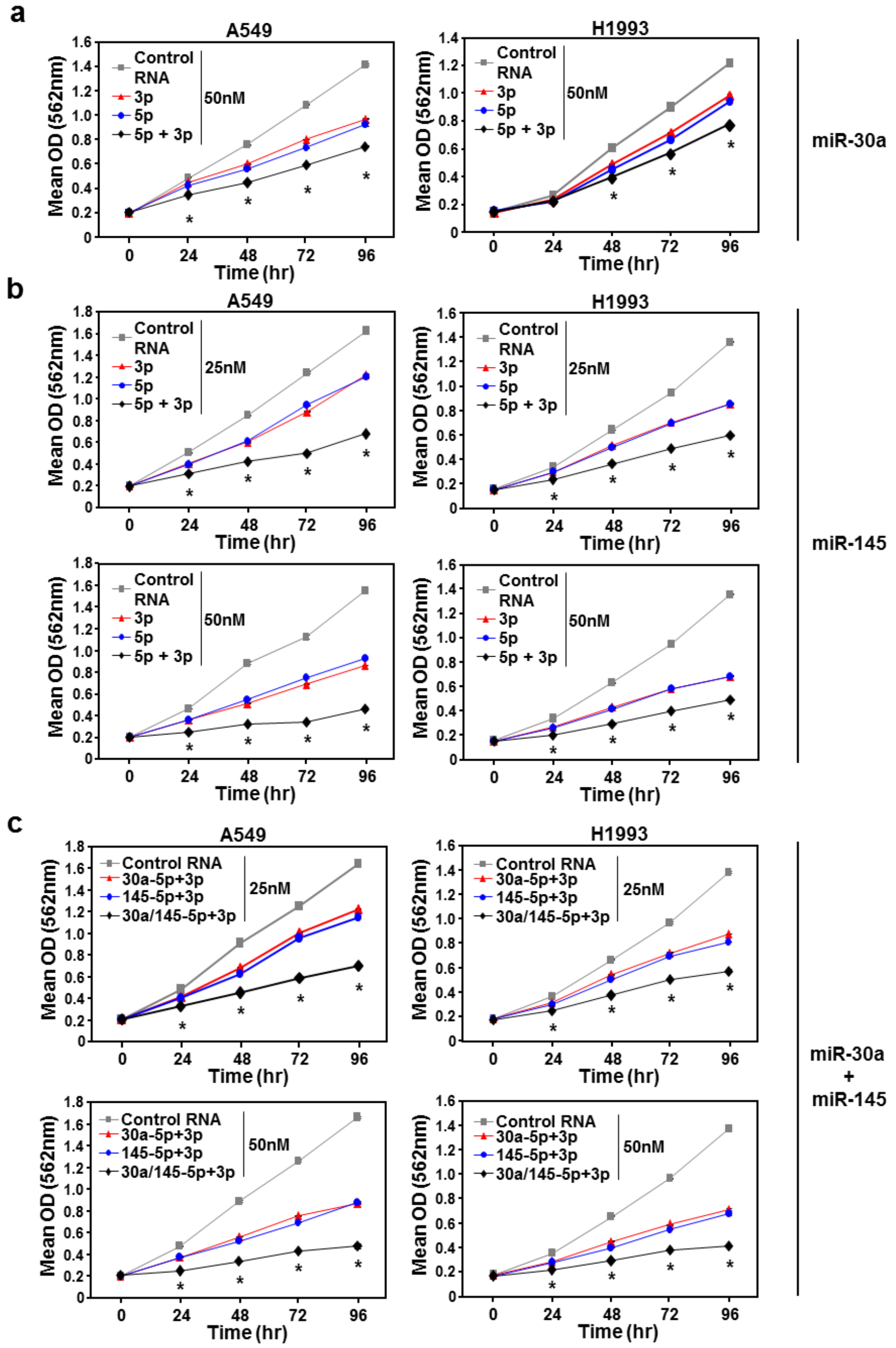

Supplementary Figure 8. Simultaneous over-expression of both strands of miR-30a and/or miR-145 cooperate to decrease lung adenocarcinoma cell growth. Lung cancer cell lines were transfected with 25 or 50nM miR-30a-5p, miR-30a-3p, miR-145-5p, miR-145-3p miRNA mimic, and/or negative control RNA. Control RNA was added to equalize the total amount of RNA transfected. MTT assays were performed at intervals, in quadruplicate. Each assay was performed 2 independent times for both cell lines and one representative experiment is shown. For a, A549, $*P<2.77\times10^{-5}$ and for H1993, $*P<2.58\times10^{-5}$; for b, A549, $*P<2.04\times10^{-5}$ at 25nM and $*P<6.65\times10^{-6}$ at 50nM and for H1993, $*P<4.19\times10^{-5}$ at 25nM and $*P<2.45\times10^{-6}$ at 50nM; for c, A549, $*P<2.96\times10^{-6}$ at 25nM and $*P<2.63\times10^{-6}$ at 50nM and for H1993, $*P<1.60\times10^{-5}$ at 25nM and $*P<1.20\times10^{-4}$ at 50nM; two-tailed *t*-tests (comparing 5p+3p to only the 5p or 3p mimic for a and b and comparing 30a-5p/3p+145-5p/3p to 30a-5p/3p or 145-5p/3p for c). Data are presented as mean values ± SEM; error bars are within the symbols.

Supplementary Table 1. Fourteen TCGA cancer types

Cancer type	TCGA ID
Bladder urothelial carcinoma	BLCA
Breast invasive carcinoma	BRCA
Cholangio carcinoma	CHOL
Esophageal carcinoma	ESCA
Kidney chromophobe	KICH
Kidney renal clear cell carcinoma	KIRC
Kidney renal papillary cell carcinoma	KIRP
Liver hepatocellular carcinoma	LIHC
Lung adenocarcinoma	LUAD
Lung squamous cell carcinoma	LUSC
Prostate adenocarcinoma	PRAD
Stomach adenocarcinoma	STAD
Thyroid carcinoma	THCA
Uterine corpus endometrial carcinoma	UCEC

Supplementary Table 2. Number of cell lines used in RNAi and CRISPR screens that originated from the indicated primary tumor sites

Cancer type	Number of cell lines	
	RNAi	CRISPR
BLCA	12	23
BRCA	80	28
ESCA	24	24
KIRC	7	6
LIHC	16	15
LUAD	55	33
LUSC	18	13
STAD	24	19
UCEC	15	16

Supplementary Table 3. miRNA whose down-regulation potentially induce survival/growth of cancer cell lines screened by RNAi

miRNA	Normalized Enrichment Score	FDR$	Cell line	Cancer type
hsa-miR-28-3p	-1.825	0.001	AGS_STOMACH	STAD
hsa-miR-28-3p	-1.785	0.002	GSU_STOMACH	STAD
hsa-miR-28-3p	-1.751	0.002	SNU601_STOMACH	STAD
hsa-miR-28-3p	-1.733	0.003	SNU719_STOMACH	STAD
hsa-miR-28-3p	-1.724	0.004	KE39_STOMACH	STAD
hsa-miR-28-3p	-1.627	0.011	MKN45_STOMACH	STAD
hsa-miR-28-3p	-1.651	0.011	NUGC3_STOMACH	STAD
hsa-miR-28-3p	-1.582	0.014	2313287_STOMACH	STAD
hsa-miR-28-3p	-1.674	0.015	NCCSTCK140_STOMACH	STAD
hsa-miR-28-3p	-1.591	0.017	OCUM1_STOMACH	STAD
hsa-miR-28-3p	-1.696	0.019	SNU216_STOMACH	STAD
hsa-miR-28-3p	-1.615	0.021	SNU5_STOMACH	STAD
hsa-miR-28-3p	-1.672	0.021	ECC10_STOMACH	STAD
hsa-miR-28-3p	-1.598	0.023	MKN1_STOMACH	STAD
hsa-miR-28-3p	-1.549	0.031	NCIN87_STOMACH	STAD
hsa-miR-28-3p	-1.507	0.031	HUG1N_STOMACH	STAD
hsa-miR-28-3p	-1.507	0.042	IM95_STOMACH	STAD
hsa-miR-28-3p	-1.469	0.046	KATOIII_STOMACH	STAD
hsa-miR-28-3p	-1.577	0.054	MKN7_STOMACH	STAD
hsa-miR-28-3p	-1.475	0.084	GSS_STOMACH	STAD
hsa-miR-28-5p	-1.733	0.002	AGS_STOMACH	STAD
hsa-miR-28-5p	-1.737	0.003	GSU_STOMACH	STAD
hsa-miR-28-5p	-1.690	0.005	MKN45_STOMACH	STAD
hsa-miR-28-5p	-1.668	0.008	SNU719_STOMACH	STAD
hsa-miR-28-5p	-1.669	0.009	OCUM1_STOMACH	STAD
hsa-miR-28-5p	-1.581	0.009	SNU601_STOMACH	STAD
hsa-miR-28-5p	-1.619	0.013	IM95_STOMACH	STAD
hsa-miR-28-5p	-1.654	0.014	SNU5_STOMACH	STAD
hsa-miR-28-5p	-1.608	0.016	NUGC3_STOMACH	STAD
hsa-miR-28-5p	-1.609	0.016	NCIN87_STOMACH	STAD
hsa-miR-28-5p	-1.554	0.020	KE39_STOMACH	STAD
hsa-miR-28-5p	-1.519	0.027	KATOIII_STOMACH	STAD
hsa-miR-28-5p	-1.535	0.036	MKN1_STOMACH	STAD
hsa-miR-28-5p	-1.475	0.046	2313287_STOMACH	STAD
hsa-miR-28-5p	-1.555	0.051	SNU216_STOMACH	STAD
hsa-miR-28-5p	-1.432	0.062	HUG1N_STOMACH	STAD
hsa-miR-28-5p	-1.457	0.083	GSS_STOMACH	STAD
hsa-miR-30a-3p	-1.787	0.006	KYSE150_OESOPHAGUS	ESCA

hsa-miR-30a-3p	-1.744	0.009	KYSE30_OESOPHAGUS	ESCA
hsa-miR-30a-3p	-1.676	0.024	TE10_OESOPHAGUS	ESCA
hsa-miR-30a-3p	-1.641	0.037	KYSE140_OESOPHAGUS	ESCA
hsa-miR-30a-3p	-1.542	0.050	TE4_OESOPHAGUS	ESCA
hsa-miR-30a-3p	-1.557	0.088	TE1_OESOPHAGUS	ESCA
hsa-miR-30a-3p	-1.885	0.005	JHH5_LIVER	LIHC
hsa-miR-30a-3p	-1.798	0.010	JHH1_LIVER	LIHC
hsa-miR-30a-3p	-1.741	0.021	HUH7_LIVER	LIHC
hsa-miR-30a-3p	-1.749	0.027	HLE_LIVER	LIHC
hsa-miR-30a-3p	-1.800	0.005	NCIH2126_LUNG	LUAD
hsa-miR-30a-3p	-1.725	0.014	LXF289_LUNG	LUAD
hsa-miR-30a-3p	-1.725	0.022	NCIH854_LUNG	LUAD
hsa-miR-30a-3p	-1.651	0.029	NCIH1623_LUNG	LUAD
hsa-miR-30a-3p	-1.726	0.033	HOP62_LUNG	LUAD
hsa-miR-30a-3p	-1.630	0.044	NCIH2122_LUNG	LUAD
hsa-miR-30a-3p	-1.522	0.045	NCIH1568_LUNG	LUAD
hsa-miR-30a-3p	-1.593	0.054	RERFLCAD2_LUNG	LUAD
hsa-miR-30a-3p	-1.585	0.057	NCIH1944_LUNG	LUAD
hsa-miR-30a-3p	-1.537	0.062	RERFLCMS_LUNG	LUAD
hsa-miR-30a-3p	-1.608	0.064	NCIH2030_LUNG	LUAD
hsa-miR-30a-3p	-1.585	0.065	NCIH1373_LUNG	LUAD
hsa-miR-30a-3p	-1.653	0.074	EKVX_LUNG	LUAD
hsa-miR-30a-3p	-1.653	0.080	HCC1833_LUNG	LUAD
hsa-miR-30a-3p	-1.544	0.081	NCIH1435_LUNG	LUAD
hsa-miR-30a-3p	-1.576	0.083	NCIH3122_LUNG	LUAD
hsa-miR-30a-3p	-1.548	0.084	NCIH838_LUNG	LUAD
hsa-miR-30a-3p	-1.640	0.096	COLO699_LUNG	LUAD
hsa-miR-30a-3p	-1.487	0.097	NCIH2009_LUNG	LUAD
hsa-miR-30a-3p	-1.556	0.099	NCIH2291_LUNG	LUAD
hsa-miR-30a-3p	-1.901	0.001	LOUNH91_LUNG	LUSC
hsa-miR-30a-3p	-1.860	0.002	KNS62_LUNG	LUSC
hsa-miR-30a-3p	-1.831	0.002	EPLC272H_LUNG	LUSC
hsa-miR-30a-3p	-1.837	0.002	CALU1_LUNG	LUSC
hsa-miR-30a-3p	-1.773	0.004	EBC1_LUNG	LUSC
hsa-miR-30a-3p	-1.748	0.004	RERFLCAI_LUNG	LUSC
hsa-miR-30a-3p	-1.731	0.006	NCIH2170_LUNG	LUSC
hsa-miR-30a-3p	-1.687	0.008	SKMES1_LUNG	LUSC
hsa-miR-30a-3p	-1.702	0.010	HCC15_LUNG	LUSC
hsa-miR-30a-3p	-1.657	0.010	LUDLU1_LUNG	LUSC
hsa-miR-30a-3p	-1.691	0.010	NCIH2882_LUNG	LUSC
hsa-miR-30a-3p	-1.649	0.012	LK2_LUNG	LUSC
hsa-miR-30a-3p	-1.640	0.012	NCIH1703_LUNG	LUSC
hsa-miR-30a-3p	-1.693	0.017	HCC2814_LUNG	LUSC

hsa-miR-30a-3p	-1.622	0.029	NCIH520_LUNG	LUSC
hsa-miR-30a-3p	-1.498	0.047	SQ1_LUNG	LUSC
hsa-miR-30a-3p	-1.566	0.051	LC1SQSF_LUNG	LUSC
hsa-miR-30a-3p	-1.545	0.056	HCC95_LUNG	LUSC
hsa-miR-30a-3p	-1.858	0.000	MKN1_STOMACH	STAD
hsa-miR-30a-3p	-2.052	0.000	GSU_STOMACH	STAD
hsa-miR-30a-3p	-1.849	0.000	AGS_STOMACH	STAD
hsa-miR-30a-3p	-1.809	0.000	SNU601_STOMACH	STAD
hsa-miR-30a-3p	-1.969	0.000	HUG1N_STOMACH	STAD
hsa-miR-30a-3p	-1.861	0.001	SNU719_STOMACH	STAD
hsa-miR-30a-3p	-1.827	0.001	KE39_STOMACH	STAD
hsa-miR-30a-3p	-1.859	0.001	SNU5_STOMACH	STAD
hsa-miR-30a-3p	-1.814	0.002	IM95_STOMACH	STAD
hsa-miR-30a-3p	-1.765	0.002	MKN45_STOMACH	STAD
hsa-miR-30a-3p	-1.741	0.003	NUGC3_STOMACH	STAD
hsa-miR-30a-3p	-1.751	0.003	KATOIII_STOMACH	STAD
hsa-miR-30a-3p	-1.857	0.003	MKN7_STOMACH	STAD
hsa-miR-30a-3p	-1.758	0.004	NCIN87_STOMACH	STAD
hsa-miR-30a-3p	-1.874	0.006	SNU216_STOMACH	STAD
hsa-miR-30a-3p	-1.688	0.007	2313287_STOMACH	STAD
hsa-miR-30a-3p	-1.656	0.008	OCUM1_STOMACH	STAD
hsa-miR-30a-3p	-1.760	0.011	NCCSTCK140_STOMACH	STAD
hsa-miR-30a-3p	-1.710	0.029	ECC10_STOMACH	STAD
hsa-miR-30a-3p	-1.710	0.050	GSS_STOMACH	STAD
hsa-miR-30a-3p	-1.624	0.050	FU97_STOMACH	STAD
hsa-miR-30a-3p	-1.681	0.053	HGC27_STOMACH	STAD
hsa-miR-30a-5p	-1.919	0.000	KYSE450_OESOPHAGUS	ESCA
hsa-miR-30a-5p	-1.842	0.004	KYSE150_OESOPHAGUS	ESCA
hsa-miR-30a-5p	-1.804	0.005	TT_OESOPHAGUS	ESCA
hsa-miR-30a-5p	-1.818	0.006	TE10_OESOPHAGUS	ESCA
hsa-miR-30a-5p	-1.752	0.009	TE1_OESOPHAGUS	ESCA
hsa-miR-30a-5p	-1.708	0.011	KYSE30_OESOPHAGUS	ESCA
hsa-miR-30a-5p	-1.776	0.023	OE33_OESOPHAGUS	ESCA
hsa-miR-30a-5p	-1.628	0.023	KYSE410_OESOPHAGUS	ESCA
hsa-miR-30a-5p	-1.605	0.023	COLO680N_OESOPHAGUS	ESCA
hsa-miR-30a-5p	-1.645	0.026	KYSE520_OESOPHAGUS	ESCA
hsa-miR-30a-5p	-1.668	0.031	KYSE140_OESOPHAGUS	ESCA
hsa-miR-30a-5p	-1.735	0.052	ECGI10_OESOPHAGUS	ESCA
hsa-miR-30a-5p	-1.602	0.053	TE11_OESOPHAGUS	ESCA
hsa-miR-30a-5p	-1.618	0.077	KYSE510_OESOPHAGUS	ESCA
hsa-miR-30a-5p	-1.491	0.086	TE4_OESOPHAGUS	ESCA
hsa-miR-30a-5p	-1.620	0.087	OE21_OESOPHAGUS	ESCA
hsa-miR-30a-5p	-1.562	0.093	KYSE70_OESOPHAGUS	ESCA

hsa-miR-30a-5p	-1.912	0.000	NCIH2126_LUNG	LUAD
hsa-miR-30a-5p	-1.865	0.003	RERFLCAD2_LUNG	LUAD
hsa-miR-30a-5p	-1.788	0.007	HCC4006_LUNG	LUAD
hsa-miR-30a-5p	-1.858	0.009	NCIH441_LUNG	LUAD
hsa-miR-30a-5p	-1.759	0.009	NCIH1623_LUNG	LUAD
hsa-miR-30a-5p	-1.748	0.013	HCC827_LUNG	LUAD
hsa-miR-30a-5p	-1.804	0.013	A549_LUNG	LUAD
hsa-miR-30a-5p	-1.752	0.014	NCIH838_LUNG	LUAD
hsa-miR-30a-5p	-1.794	0.015	NCIH1437_LUNG	LUAD
hsa-miR-30a-5p	-1.725	0.016	NCIH2009_LUNG	LUAD
hsa-miR-30a-5p	-1.802	0.027	HCC1833_LUNG	LUAD
hsa-miR-30a-5p	-1.709	0.033	HCC2450_LUNG	LUAD
hsa-miR-30a-5p	-1.769	0.033	NCIH3255_LUNG	LUAD
hsa-miR-30a-5p	-1.784	0.035	NCIH2030_LUNG	LUAD
hsa-miR-30a-5p	-1.715	0.039	NCIH2122_LUNG	LUAD
hsa-miR-30a-5p	-1.739	0.039	NCIH2228_LUNG	LUAD
hsa-miR-30a-5p	-1.608	0.043	NCIH854_LUNG	LUAD
hsa-miR-30a-5p	-1.675	0.044	NCIH2087_LUNG	LUAD
hsa-miR-30a-5p	-1.603	0.049	LXF289_LUNG	LUAD
hsa-miR-30a-5p	-1.618	0.053	NCIH1944_LUNG	LUAD
hsa-miR-30a-5p	-1.484	0.055	NCIH1568_LUNG	LUAD
hsa-miR-30a-5p	-1.612	0.061	NCIH1373_LUNG	LUAD
hsa-miR-30a-5p	-1.609	0.074	NCIH2291_LUNG	LUAD
hsa-miR-30a-5p	-1.741	0.078	NCIH3122_LUNG	LUAD
hsa-miR-30a-5p	-1.653	0.084	HLC1_LUNG	LUAD
hsa-miR-30a-5p	-1.663	0.084	HCC44_LUNG	LUAD
hsa-miR-30a-5p	-1.662	0.087	NCIH1792_LUNG	LUAD
hsa-miR-30a-5p	-1.658	0.088	NCIH1975_LUNG	LUAD
hsa-miR-30a-5p	-1.555	0.091	HOP62_LUNG	LUAD
hsa-miR-30a-5p	-1.814	0.001	KNS62_LUNG	LUSC
hsa-miR-30a-5p	-1.871	0.001	HCC15_LUNG	LUSC
hsa-miR-30a-5p	-1.795	0.004	NCIH2170_LUNG	LUSC
hsa-miR-30a-5p	-1.725	0.005	LUDLU1_LUNG	LUSC
hsa-miR-30a-5p	-1.720	0.005	LOUNH91_LUNG	LUSC
hsa-miR-30a-5p	-1.727	0.005	RERFLCAI_LUNG	LUSC
hsa-miR-30a-5p	-1.670	0.007	EBC1_LUNG	LUSC
hsa-miR-30a-5p	-1.656	0.012	EPLC272H_LUNG	LUSC
hsa-miR-30a-5p	-1.624	0.013	SKMES1_LUNG	LUSC
hsa-miR-30a-5p	-1.606	0.013	NCIH1703_LUNG	LUSC
hsa-miR-30a-5p	-1.784	0.014	LC1SQSF_LUNG	LUSC
hsa-miR-30a-5p	-1.614	0.015	LK2_LUNG	LUSC
hsa-miR-30a-5p	-1.627	0.017	NCIH2882_LUNG	LUSC
hsa-miR-30a-5p	-1.603	0.031	SQ1_LUNG	LUSC

hsa-miR-30a-5p	-1.605	0.033	HCC2814_LUNG	LUSC
hsa-miR-30a-5p	-1.529	0.036	NCIH520_LUNG	LUSC
hsa-miR-30a-5p	-1.652	0.053	HCC95_LUNG	LUSC
hsa-miR-30a-5p	-1.398	0.088	CALU1_LUNG	LUSC
hsa-miR-30a-5p	-1.860	0.001	GSU_STOMACH	STAD
hsa-miR-30a-5p	-1.910	0.001	HUG1N_STOMACH	STAD
hsa-miR-30a-5p	-1.750	0.002	SNU601_STOMACH	STAD
hsa-miR-30a-5p	-1.753	0.003	IM95_STOMACH	STAD
hsa-miR-30a-5p	-1.707	0.005	NUGC3_STOMACH	STAD
hsa-miR-30a-5p	-1.648	0.010	SNU719_STOMACH	STAD
hsa-miR-30a-5p	-1.659	0.014	SNU5_STOMACH	STAD
hsa-miR-30a-5p	-1.595	0.015	MKN45_STOMACH	STAD
hsa-miR-30a-5p	-1.573	0.015	2313287_STOMACH	STAD
hsa-miR-30a-5p	-1.603	0.017	NCIN87_STOMACH	STAD
hsa-miR-30a-5p	-1.564	0.019	KE39_STOMACH	STAD
hsa-miR-30a-5p	-1.637	0.019	NCCSTCK140_STOMACH	STAD
hsa-miR-30a-5p	-1.568	0.021	AGS_STOMACH	STAD
hsa-miR-30a-5p	-1.589	0.039	SNU216_STOMACH	STAD
hsa-miR-30a-5p	-1.526	0.039	MKN1_STOMACH	STAD
hsa-miR-30a-5p	-1.480	0.042	KATOIII_STOMACH	STAD
hsa-miR-30a-5p	-1.602	0.043	MKN7_STOMACH	STAD
hsa-miR-30a-5p	-1.551	0.045	GSS_STOMACH	STAD
hsa-miR-30a-5p	-1.590	0.051	FU97_STOMACH	STAD
hsa-miR-30a-5p	-1.623	0.056	HGC27_STOMACH	STAD
hsa-miR-30a-5p	-1.490	0.057	OCUM1_STOMACH	STAD
hsa-miR-139-3p	-1.601	0.050	RERFLCMS_LUNG	LUAD
hsa-miR-139-3p	-1.634	0.051	NCIH1435_LUNG	LUAD
hsa-miR-139-3p	-1.464	0.095	RERFLCAD2_LUNG	LUAD
hsa-miR-139-3p	-1.495	0.095	NCIH2009_LUNG	LUAD
hsa-miR-139-3p	-1.454	0.099	NCIH1623_LUNG	LUAD
hsa-miR-139-5p	-1.663	0.019	UBLC1_URINARY_TRACT	BLCA
hsa-miR-139-5p	-1.631	0.056	CAL29_URINARY_TRACT	BLCA
hsa-miR-139-5p	-2.126	0.000	MDAMB231_BREAST	BRCA
hsa-miR-139-5p	-1.974	0.000	MDAMB415_BREAST	BRCA
hsa-miR-139-5p	-1.955	0.000	CAMA1_BREAST	BRCA
hsa-miR-139-5p	-1.802	0.001	HCC1419_BREAST	BRCA
hsa-miR-139-5p	-1.903	0.001	HCC1806_BREAST	BRCA
hsa-miR-139-5p	-1.894	0.001	ZR751_BREAST	BRCA
hsa-miR-139-5p	-1.905	0.001	MDAMB361_BREAST	BRCA
hsa-miR-139-5p	-1.885	0.001	HS578T_BREAST	BRCA
hsa-miR-139-5p	-1.884	0.002	SKBR5_BREAST	BRCA
hsa-miR-139-5p	-1.852	0.002	MACLS2_BREAST	BRCA
hsa-miR-139-5p	-1.882	0.002	HCC38_BREAST	BRCA

hsa-miR-139-5p	-1.804	0.002	HCC1954_BREAST	BRCA
hsa-miR-139-5p	-1.876	0.002	MX1_BREAST	BRCA
hsa-miR-139-5p	-1.850	0.003	HCC1428_BREAST	BRCA
hsa-miR-139-5p	-1.820	0.003	CAL851_BREAST	BRCA
hsa-miR-139-5p	-1.847	0.003	MDAMB468_BREAST	BRCA
hsa-miR-139-5p	-1.875	0.003	MDAMB175VII_BREAST	BRCA
hsa-miR-139-5p	-1.837	0.003	SUM149PT_BREAST	BRCA
hsa-miR-139-5p	-1.865	0.003	CAL148_BREAST	BRCA
hsa-miR-139-5p	-1.901	0.004	184A1_BREAST	BRCA
hsa-miR-139-5p	-1.893	0.005	ZR75B_BREAST	BRCA
hsa-miR-139-5p	-1.898	0.005	BT549_BREAST	BRCA
hsa-miR-139-5p	-1.757	0.006	JIMT1_BREAST	BRCA
hsa-miR-139-5p	-1.776	0.006	SW527_BREAST	BRCA
hsa-miR-139-5p	-1.794	0.007	CAL51_BREAST	BRCA
hsa-miR-139-5p	-1.801	0.008	BT20_BREAST	BRCA
hsa-miR-139-5p	-1.773	0.008	MCF12A_BREAST	BRCA
hsa-miR-139-5p	-1.742	0.009	MDAMB436_BREAST	BRCA
hsa-miR-139-5p	-1.768	0.009	KPL1_BREAST	BRCA
hsa-miR-139-5p	-1.743	0.010	MB157_BREAST	BRCA
hsa-miR-139-5p	-1.773	0.010	EFM19_BREAST	BRCA
hsa-miR-139-5p	-1.709	0.010	HCC2218_BREAST	BRCA
hsa-miR-139-5p	-1.719	0.011	HCC70_BREAST	BRCA
hsa-miR-139-5p	-1.799	0.013	HCC3153_BREAST	BRCA
hsa-miR-139-5p	-1.712	0.013	UACC812_BREAST	BRCA
hsa-miR-139-5p	-1.679	0.015	HCC1937_BREAST	BRCA
hsa-miR-139-5p	-1.672	0.015	MDAMB330_BREAST	BRCA
hsa-miR-139-5p	-1.687	0.015	SUM185PE_BREAST	BRCA
hsa-miR-139-5p	-1.815	0.017	SUM159PT_BREAST	BRCA
hsa-miR-139-5p	-1.723	0.017	HCC1500_BREAST	BRCA
hsa-miR-139-5p	-1.813	0.018	EFM192A_BREAST	BRCA
hsa-miR-139-5p	-1.729	0.018	HDQP1_BREAST	BRCA
hsa-miR-139-5p	-1.677	0.022	MDAMB453_BREAST	BRCA
hsa-miR-139-5p	-1.730	0.023	HCC1143_BREAST	BRCA
hsa-miR-139-5p	-1.820	0.024	MDAMB157_BREAST	BRCA
hsa-miR-139-5p	-1.713	0.028	HCC1395_BREAST	BRCA
hsa-miR-139-5p	-1.703	0.029	MCF7_BREAST	BRCA
hsa-miR-139-5p	-1.659	0.029	T47D_BREAST	BRCA
hsa-miR-139-5p	-1.694	0.030	EVSAT_BREAST	BRCA
hsa-miR-139-5p	-1.649	0.031	SUM190PT_BREAST	BRCA
hsa-miR-139-5p	-1.707	0.042	SKBR7_BREAST	BRCA
hsa-miR-139-5p	-1.564	0.047	SUM1315MO2_BREAST	BRCA
hsa-miR-139-5p	-1.748	0.050	DU4475_BREAST	BRCA
hsa-miR-139-5p	-1.629	0.051	BT483_BREAST	BRCA

hsa-miR-139-5p	-1.667	0.052	BT474_BREAST	BRCA
hsa-miR-139-5p	-1.638	0.055	HCC202_BREAST	BRCA
hsa-miR-139-5p	-1.669	0.057	SUM52PE_BREAST	BRCA
hsa-miR-139-5p	-1.609	0.058	HCC1008_BREAST	BRCA
hsa-miR-139-5p	-1.598	0.061	HMC18_BREAST	BRCA
hsa-miR-139-5p	-1.655	0.062	SUM225CWN_BREAST	BRCA
hsa-miR-139-5p	-1.658	0.065	600MPE_BREAST	BRCA
hsa-miR-139-5p	-1.721	0.074	HCC1569_BREAST	BRCA
hsa-miR-139-5p	-1.623	0.078	HCC2185_BREAST	BRCA
hsa-miR-139-5p	-1.590	0.081	LY2_BREAST	BRCA
hsa-miR-139-5p	-1.636	0.081	CAL120_BREAST	BRCA
hsa-miR-139-5p	-1.577	0.082	UACC893_BREAST	BRCA
hsa-miR-139-5p	-1.626	0.087	HCC1187_BREAST	BRCA
hsa-miR-139-5p	-1.597	0.090	OCUBM_BREAST	BRCA
hsa-miR-139-5p	-1.605	0.096	SKBR3_BREAST	BRCA
hsa-miR-139-5p	-1.594	0.096	AU565_BREAST	BRCA
hsa-miR-139-5p	-1.661	0.025	TE1_OESOPHAGUS	ESCA
hsa-miR-139-5p	-1.603	0.027	TE4_OESOPHAGUS	ESCA
hsa-miR-139-5p	-1.656	0.028	KYSE520_OESOPHAGUS	ESCA
hsa-miR-139-5p	-1.623	0.030	COLO680N_OESOPHAGUS	ESCA
hsa-miR-139-5p	-1.580	0.044	KYSE30_OESOPHAGUS	ESCA
hsa-miR-139-5p	-1.600	0.046	KYSE140_OESOPHAGUS	ESCA
hsa-miR-139-5p	-1.609	0.051	KYSE150_OESOPHAGUS	ESCA
hsa-miR-139-5p	-1.567	0.069	TE8_OESOPHAGUS	ESCA
hsa-miR-139-5p	-1.555	0.083	TE10_OESOPHAGUS	ESCA
hsa-miR-139-5p	-1.663	0.053	JHH5_LIVER	LIHC
hsa-miR-139-5p	-1.982	0.001	LXF289_LUNG	LUAD
hsa-miR-139-5p	-1.871	0.002	NCIH2126_LUNG	LUAD
hsa-miR-139-5p	-1.960	0.002	NCIH854_LUNG	LUAD
hsa-miR-139-5p	-1.938	0.002	NCIH1792_LUNG	LUAD
hsa-miR-139-5p	-1.825	0.005	NCIH1623_LUNG	LUAD
hsa-miR-139-5p	-1.790	0.007	HCC827_LUNG	LUAD
hsa-miR-139-5p	-1.770	0.008	HCC4006_LUNG	LUAD
hsa-miR-139-5p	-1.782	0.009	RERFLCMS_LUNG	LUAD
hsa-miR-139-5p	-1.773	0.009	NCIH1944_LUNG	LUAD
hsa-miR-139-5p	-1.870	0.009	HLC1_LUNG	LUAD
hsa-miR-139-5p	-1.794	0.013	RERFLCAD2_LUNG	LUAD
hsa-miR-139-5p	-1.838	0.014	NCIH2291_LUNG	LUAD
hsa-miR-139-5p	-1.823	0.014	NCIH2009_LUNG	LUAD
hsa-miR-139-5p	-1.838	0.014	HOP62_LUNG	LUAD
hsa-miR-139-5p	-1.742	0.016	NCIH1437_LUNG	LUAD
hsa-miR-139-5p	-1.783	0.018	NCIH2030_LUNG	LUAD
hsa-miR-139-5p	-1.659	0.027	NCIH1568_LUNG	LUAD

hsa-miR-139-5p	-1.671	0.028	NCIH838_LUNG	LUAD
hsa-miR-139-5p	-1.789	0.030	HCC2450_LUNG	LUAD
hsa-miR-139-5p	-1.746	0.031	ABC1_LUNG	LUAD
hsa-miR-139-5p	-1.829	0.031	CORL105_LUNG	LUAD
hsa-miR-139-5p	-1.698	0.031	A549_LUNG	LUAD
hsa-miR-139-5p	-1.764	0.035	NCIH2122_LUNG	LUAD
hsa-miR-139-5p	-1.776	0.040	CALU3_LUNG	LUAD
hsa-miR-139-5p	-1.750	0.042	NCIH1435_LUNG	LUAD
hsa-miR-139-5p	-1.710	0.049	NCIH2087_LUNG	LUAD
hsa-miR-139-5p	-1.707	0.049	HCC461_LUNG	LUAD
hsa-miR-139-5p	-1.705	0.061	A427_LUNG	LUAD
hsa-miR-139-5p	-1.770	0.064	NCIH3255_LUNG	LUAD
hsa-miR-139-5p	-1.673	0.064	NCIH3122_LUNG	LUAD
hsa-miR-139-5p	-1.672	0.066	NCIH1355_LUNG	LUAD
hsa-miR-139-5p	-1.648	0.068	HCC515_LUNG	LUAD
hsa-miR-139-5p	-1.607	0.072	COLO699_LUNG	LUAD
hsa-miR-139-5p	-1.614	0.075	NCIH1373_LUNG	LUAD
hsa-miR-139-5p	-1.619	0.076	NCIH23_LUNG	LUAD
hsa-miR-139-5p	-1.655	0.077	NCIH1648_LUNG	LUAD
hsa-miR-139-5p	-1.638	0.084	NCIH2405_LUNG	LUAD
hsa-miR-139-5p	-1.686	0.088	HCC44_LUNG	LUAD
hsa-miR-139-5p	-1.690	0.091	NCIH1975_LUNG	LUAD
hsa-miR-139-5p	-1.662	0.094	MORCPR_LUNG	LUAD
hsa-miR-139-5p	-1.711	0.096	EKVX_LUNG	LUAD
hsa-miR-139-5p	-1.813	0.002	SKMES1_LUNG	LUSC
hsa-miR-139-5p	-1.779	0.003	LOUNH91_LUNG	LUSC
hsa-miR-139-5p	-1.794	0.003	RERFLCAI_LUNG	LUSC
hsa-miR-139-5p	-1.717	0.006	EBC1_LUNG	LUSC
hsa-miR-139-5p	-1.727	0.011	HCC15_LUNG	LUSC
hsa-miR-139-5p	-1.643	0.011	LUDLU1_LUNG	LUSC
hsa-miR-139-5p	-1.720	0.012	LK2_LUNG	LUSC
hsa-miR-139-5p	-1.611	0.012	KNS62_LUNG	LUSC
hsa-miR-139-5p	-1.631	0.015	EPLC272H_LUNG	LUSC
hsa-miR-139-5p	-1.624	0.016	CALU1_LUNG	LUSC
hsa-miR-139-5p	-1.621	0.016	NCIH2882_LUNG	LUSC
hsa-miR-139-5p	-1.553	0.026	NCIH1703_LUNG	LUSC
hsa-miR-139-5p	-1.641	0.037	NCIH520_LUNG	LUSC
hsa-miR-139-5p	-1.599	0.040	HCC95_LUNG	LUSC
hsa-miR-139-5p	-1.490	0.044	NCIH2170_LUNG	LUSC
hsa-miR-139-5p	-1.584	0.057	LC1SQSF_LUNG	LUSC
hsa-miR-139-5p	-1.950	0.000	GSU_STOMACH	STAD
hsa-miR-139-5p	-1.772	0.001	AGS_STOMACH	STAD
hsa-miR-139-5p	-1.742	0.001	SNU601_STOMACH	STAD

hsa-miR-139-5p	-1.789	0.002	MKN45_STOMACH	STAD
hsa-miR-139-5p	-1.806	0.003	NCIN87_STOMACH	STAD
hsa-miR-139-5p	-1.752	0.003	SNU719_STOMACH	STAD
hsa-miR-139-5p	-1.798	0.003	NUGC3_STOMACH	STAD
hsa-miR-139-5p	-1.761	0.003	IM95_STOMACH	STAD
hsa-miR-139-5p	-1.736	0.005	SNU5_STOMACH	STAD
hsa-miR-139-5p	-1.714	0.005	HUG1N_STOMACH	STAD
hsa-miR-139-5p	-1.655	0.008	KE39_STOMACH	STAD
hsa-miR-139-5p	-1.643	0.012	MKN1_STOMACH	STAD
hsa-miR-139-5p	-1.597	0.014	2313287_STOMACH	STAD
hsa-miR-139-5p	-1.589	0.015	KATOIII_STOMACH	STAD
hsa-miR-139-5p	-1.695	0.016	SNU216_STOMACH	STAD
hsa-miR-139-5p	-1.522	0.059	GSS_STOMACH	STAD
hsa-miR-139-5p	-1.522	0.082	ECC10_STOMACH	STAD
hsa-miR-139-5p	-1.520	0.082	FU97_STOMACH	STAD
hsa-miR-139-5p	-1.479	0.086	NCCSTCK140_STOMACH	STAD
hsa-miR-143-3p	-2.053	0.000	MCF7_BREAST	BRCA
hsa-miR-143-3p	-2.046	0.000	HCC1500_BREAST	BRCA
hsa-miR-143-3p	-1.953	0.000	MDAMB231_BREAST	BRCA
hsa-miR-143-3p	-1.930	0.000	CAL148_BREAST	BRCA
hsa-miR-143-3p	-1.908	0.000	HCC1008_BREAST	BRCA
hsa-miR-143-3p	-1.906	0.000	SUM149PT_BREAST	BRCA
hsa-miR-143-3p	-1.900	0.000	ZR7530_BREAST	BRCA
hsa-miR-143-3p	-1.887	0.000	HCC1806_BREAST	BRCA
hsa-miR-143-3p	-1.960	0.001	HS578T_BREAST	BRCA
hsa-miR-143-3p	-2.047	0.001	MX1_BREAST	BRCA
hsa-miR-143-3p	-1.883	0.001	MDAMB415_BREAST	BRCA
hsa-miR-143-3p	-1.917	0.001	JIMT1_BREAST	BRCA
hsa-miR-143-3p	-1.865	0.001	HCC2218_BREAST	BRCA
hsa-miR-143-3p	-1.868	0.001	KPL1_BREAST	BRCA
hsa-miR-143-3p	-1.881	0.001	HCC1954_BREAST	BRCA
hsa-miR-143-3p	-1.894	0.002	HCC38_BREAST	BRCA
hsa-miR-143-3p	-1.925	0.002	SKBR5_BREAST	BRCA
hsa-miR-143-3p	-1.854	0.002	CAL851_BREAST	BRCA
hsa-miR-143-3p	-1.825	0.002	CAMA1_BREAST	BRCA
hsa-miR-143-3p	-1.841	0.002	HCC1419_BREAST	BRCA
hsa-miR-143-3p	-1.832	0.002	MCF12A_BREAST	BRCA
hsa-miR-143-3p	-1.812	0.002	UACC893_BREAST	BRCA
hsa-miR-143-3p	-1.872	0.003	BT483_BREAST	BRCA
hsa-miR-143-3p	-1.937	0.003	MDAMB175VII_BREAST	BRCA
hsa-miR-143-3p	-1.833	0.003	ZR751_BREAST	BRCA
hsa-miR-143-3p	-1.833	0.004	UACC812_BREAST	BRCA
hsa-miR-143-3p	-1.819	0.004	MDAMB436_BREAST	BRCA

hsa-miR-143-3p	-1.893	0.004	T47D_BREAST	BRCA
hsa-miR-143-3p	-1.855	0.004	MDAMB361_BREAST	BRCA
hsa-miR-143-3p	-1.818	0.004	MDAMB468_BREAST	BRCA
hsa-miR-143-3p	-1.826	0.005	CAL51_BREAST	BRCA
hsa-miR-143-3p	-1.870	0.005	OCUBM_BREAST	BRCA
hsa-miR-143-3p	-1.879	0.005	SUM190PT_BREAST	BRCA
hsa-miR-143-3p	-1.919	0.005	184A1_BREAST	BRCA
hsa-miR-143-3p	-1.769	0.005	MDAMB453_BREAST	BRCA
hsa-miR-143-3p	-1.769	0.007	EFM19_BREAST	BRCA
hsa-miR-143-3p	-1.753	0.007	HCC70_BREAST	BRCA
hsa-miR-143-3p	-1.803	0.007	MDAMB330_BREAST	BRCA
hsa-miR-143-3p	-1.740	0.007	MB157_BREAST	BRCA
hsa-miR-143-3p	-1.739	0.008	HCC1937_BREAST	BRCA
hsa-miR-143-3p	-1.823	0.009	MDAMB134VI_BREAST	BRCA
hsa-miR-143-3p	-1.803	0.010	HCC1143_BREAST	BRCA
hsa-miR-143-3p	-1.690	0.012	MACLS2_BREAST	BRCA
hsa-miR-143-3p	-1.825	0.012	HCC1395_BREAST	BRCA
hsa-miR-143-3p	-1.741	0.013	SUM185PE_BREAST	BRCA
hsa-miR-143-3p	-1.743	0.013	HCC3153_BREAST	BRCA
hsa-miR-143-3p	-1.743	0.016	ZR75B_BREAST	BRCA
hsa-miR-143-3p	-1.761	0.017	BT549_BREAST	BRCA
hsa-miR-143-3p	-1.734	0.019	EVSAT_BREAST	BRCA
hsa-miR-143-3p	-1.756	0.019	HCC202_BREAST	BRCA
hsa-miR-143-3p	-1.789	0.020	HCC2688_BREAST	BRCA
hsa-miR-143-3p	-1.696	0.021	BT20_BREAST	BRCA
hsa-miR-143-3p	-1.694	0.025	HDQP1_BREAST	BRCA
hsa-miR-143-3p	-1.607	0.028	SW527_BREAST	BRCA
hsa-miR-143-3p	-1.673	0.029	HMC18_BREAST	BRCA
hsa-miR-143-3p	-1.648	0.031	HCC1428_BREAST	BRCA
hsa-miR-143-3p	-1.680	0.039	EFM192A_BREAST	BRCA
hsa-miR-143-3p	-1.637	0.043	SUM1315MO2_BREAST	BRCA
hsa-miR-143-3p	-1.717	0.055	SUM52PE_BREAST	BRCA
hsa-miR-143-3p	-1.616	0.060	BT474_BREAST	BRCA
hsa-miR-143-3p	-1.610	0.063	HCC2185_BREAST	BRCA
hsa-miR-143-3p	-1.684	0.064	HCC1569_BREAST	BRCA
hsa-miR-143-3p	-1.680	0.065	LY2_BREAST	BRCA
hsa-miR-143-3p	-1.623	0.068	YMB1_BREAST	BRCA
hsa-miR-143-3p	-1.661	0.069	MDAMB157_BREAST	BRCA
hsa-miR-143-3p	-1.628	0.071	DU4475_BREAST	BRCA
hsa-miR-143-3p	-1.698	0.072	600MPE_BREAST	BRCA
hsa-miR-143-3p	-1.661	0.077	SKBR3_BREAST	BRCA
hsa-miR-143-3p	-1.556	0.077	AU565_BREAST	BRCA
hsa-miR-143-3p	-1.600	0.096	HCC1187_BREAST	BRCA

hsa-miR-143-3p	-1.576	0.099	SUM44PE_BREAST	BRCA
hsa-miR-143-3p	-1.881	0.001	TE1_OESOPHAGUS	ESCA
hsa-miR-143-3p	-1.882	0.002	TE4_OESOPHAGUS	ESCA
hsa-miR-143-3p	-1.757	0.003	COLO680N_OESOPHAGUS	ESCA
hsa-miR-143-3p	-1.822	0.005	KYSE520_OESOPHAGUS	ESCA
hsa-miR-143-3p	-1.809	0.005	KYSE30_OESOPHAGUS	ESCA
hsa-miR-143-3p	-1.813	0.007	KYSE450_OESOPHAGUS	ESCA
hsa-miR-143-3p	-1.759	0.010	KYSE140_OESOPHAGUS	ESCA
hsa-miR-143-3p	-1.712	0.016	TE10_OESOPHAGUS	ESCA
hsa-miR-143-3p	-1.740	0.022	TE8_OESOPHAGUS	ESCA
hsa-miR-143-3p	-1.592	0.035	KYSE410_OESOPHAGUS	ESCA
hsa-miR-143-3p	-1.647	0.045	KYSE150_OESOPHAGUS	ESCA
hsa-miR-143-3p	-1.650	0.048	KYSE70_OESOPHAGUS	ESCA
hsa-miR-143-3p	-1.576	0.064	TE11_OESOPHAGUS	ESCA
hsa-miR-143-3p	-1.641	0.075	TE6_OESOPHAGUS	ESCA
hsa-miR-143-3p	-1.571	0.078	TE14_OESOPHAGUS	ESCA
hsa-miR-143-3p	-1.563	0.096	JHESOAD1_OESOPHAGUS	ESCA
hsa-miR-143-3p	-1.555	0.041	NCIH1623_LUNG	LUAD
hsa-miR-143-3p	-1.452	0.074	NCIH1568_LUNG	LUAD
hsa-miR-143-3p	-1.557	0.082	NCIH2009_LUNG	LUAD
hsa-miR-143-3p	-2.068	0.001	LOUNH91_LUNG	LUSC
hsa-miR-143-3p	-1.786	0.002	SKMES1_LUNG	LUSC
hsa-miR-143-3p	-1.782	0.003	EPLC272H_LUNG	LUSC
hsa-miR-143-3p	-1.802	0.003	CALU1_LUNG	LUSC
hsa-miR-143-3p	-1.772	0.003	RERFLCAI_LUNG	LUSC
hsa-miR-143-3p	-1.714	0.004	KNS62_LUNG	LUSC
hsa-miR-143-3p	-1.705	0.006	EBC1_LUNG	LUSC
hsa-miR-143-3p	-1.688	0.008	NCIH2170_LUNG	LUSC
hsa-miR-143-3p	-1.770	0.008	LK2_LUNG	LUSC
hsa-miR-143-3p	-1.823	0.009	HCC2814_LUNG	LUSC
hsa-miR-143-3p	-1.669	0.010	LUDLU1_LUNG	LUSC
hsa-miR-143-3p	-1.713	0.010	HCC15_LUNG	LUSC
hsa-miR-143-3p	-1.640	0.010	NCIH1703_LUNG	LUSC
hsa-miR-143-3p	-1.593	0.021	NCIH2882_LUNG	LUSC
hsa-miR-143-3p	-1.651	0.024	SQ1_LUNG	LUSC
hsa-miR-143-3p	-1.633	0.029	NCIH520_LUNG	LUSC
hsa-miR-143-3p	-1.602	0.042	HCC95_LUNG	LUSC
hsa-miR-143-3p	-1.614	0.065	LC1SQSF_LUNG	LUSC
hsa-miR-143-3p	-1.959	0.000	GSU_STOMACH	STAD
hsa-miR-143-3p	-1.982	0.000	HUG1N_STOMACH	STAD
hsa-miR-143-3p	-1.909	0.000	SNU719_STOMACH	STAD
hsa-miR-143-3p	-1.823	0.001	AGS_STOMACH	STAD
hsa-miR-143-3p	-1.868	0.001	SNU5_STOMACH	STAD

hsa-miR-143-3p	-1.741	0.001	SNU601_STOMACH	STAD
hsa-miR-143-3p	-1.768	0.001	OCUM1_STOMACH	STAD
hsa-miR-143-3p	-1.812	0.001	IM95_STOMACH	STAD
hsa-miR-143-3p	-1.781	0.002	MKN45_STOMACH	STAD
hsa-miR-143-3p	-1.768	0.003	NUGC3_STOMACH	STAD
hsa-miR-143-3p	-1.765	0.004	NCIN87_STOMACH	STAD
hsa-miR-143-3p	-1.746	0.005	KE39_STOMACH	STAD
hsa-miR-143-3p	-1.734	0.006	MKN1_STOMACH	STAD
hsa-miR-143-3p	-1.660	0.008	2313287_STOMACH	STAD
hsa-miR-143-3p	-1.628	0.012	KATOIII_STOMACH	STAD
hsa-miR-143-3p	-1.695	0.014	NCCSTCK140_STOMACH	STAD
hsa-miR-143-3p	-1.684	0.016	SNU216_STOMACH	STAD
hsa-miR-143-3p	-1.636	0.032	ECC10_STOMACH	STAD
hsa-miR-143-3p	-1.620	0.047	GSS_STOMACH	STAD
hsa-miR-143-3p	-1.583	0.075	HGC27_STOMACH	STAD
hsa-miR-143-3p	-1.527	0.085	MKN7_STOMACH	STAD
hsa-miR-143-5p	-1.680	0.006	GSU_STOMACH	STAD
hsa-miR-143-5p	-1.613	0.012	SNU719_STOMACH	STAD
hsa-miR-143-5p	-1.603	0.014	MKN45_STOMACH	STAD
hsa-miR-143-5p	-1.582	0.020	HUG1N_STOMACH	STAD
hsa-miR-143-5p	-1.524	0.039	IM95_STOMACH	STAD
hsa-miR-143-5p	-1.448	0.045	SNU601_STOMACH	STAD
hsa-miR-143-5p	-1.479	0.059	NCIN87_STOMACH	STAD
hsa-miR-143-5p	-1.476	0.062	MKN1_STOMACH	STAD
hsa-miR-145-3p	-1.695	0.018	UBLC1_URINARY_TRACT	BLCA
hsa-miR-145-3p	-1.587	0.097	RT112_URINARY_TRACT	BLCA
hsa-miR-145-3p	-1.787	0.001	HCC1806_BREAST	BRCA
hsa-miR-145-3p	-1.837	0.001	HCC1954_BREAST	BRCA
hsa-miR-145-3p	-1.772	0.003	MDAMB231_BREAST	BRCA
hsa-miR-145-3p	-1.807	0.005	HS578T_BREAST	BRCA
hsa-miR-145-3p	-1.805	0.006	HDQP1_BREAST	BRCA
hsa-miR-145-3p	-1.733	0.008	MX1_BREAST	BRCA
hsa-miR-145-3p	-1.827	0.008	SW527_BREAST	BRCA
hsa-miR-145-3p	-1.718	0.008	MACLS2_BREAST	BRCA
hsa-miR-145-3p	-1.750	0.009	CAMA1_BREAST	BRCA
hsa-miR-145-3p	-1.721	0.011	SUM185PE_BREAST	BRCA
hsa-miR-145-3p	-1.708	0.012	CAL51_BREAST	BRCA
hsa-miR-145-3p	-1.740	0.013	MDAMB361_BREAST	BRCA
hsa-miR-145-3p	-1.789	0.013	EVSAT_BREAST	BRCA
hsa-miR-145-3p	-1.714	0.015	KPL1_BREAST	BRCA
hsa-miR-145-3p	-1.655	0.017	MDAMB330_BREAST	BRCA
hsa-miR-145-3p	-1.647	0.017	CAL851_BREAST	BRCA
hsa-miR-145-3p	-1.722	0.019	HCC1143_BREAST	BRCA

hsa-miR-145-3p	-1.656	0.020	HCC1937_BREAST	BRCA
hsa-miR-145-3p	-1.599	0.020	HCC1419_BREAST	BRCA
hsa-miR-145-3p	-1.704	0.021	BT20_BREAST	BRCA
hsa-miR-145-3p	-1.634	0.024	HCC2218_BREAST	BRCA
hsa-miR-145-3p	-1.699	0.028	HCC1395_BREAST	BRCA
hsa-miR-145-3p	-1.754	0.033	LY2_BREAST	BRCA
hsa-miR-145-3p	-1.639	0.034	T47D_BREAST	BRCA
hsa-miR-145-3p	-1.838	0.037	MDAMB157_BREAST	BRCA
hsa-miR-145-3p	-1.631	0.040	MDAMB415_BREAST	BRCA
hsa-miR-145-3p	-1.611	0.042	SUM1315MO2_BREAST	BRCA
hsa-miR-145-3p	-1.613	0.043	MDAMB453_BREAST	BRCA
hsa-miR-145-3p	-1.612	0.045	BT483_BREAST	BRCA
hsa-miR-145-3p	-1.596	0.046	MDAMB436_BREAST	BRCA
hsa-miR-145-3p	-1.682	0.047	DU4475_BREAST	BRCA
hsa-miR-145-3p	-1.649	0.053	SKBR7_BREAST	BRCA
hsa-miR-145-3p	-1.579	0.058	ZR751_BREAST	BRCA
hsa-miR-145-3p	-1.564	0.061	HCC70_BREAST	BRCA
hsa-miR-145-3p	-1.631	0.064	CAL120_BREAST	BRCA
hsa-miR-145-3p	-1.552	0.069	HCC3153_BREAST	BRCA
hsa-miR-145-3p	-1.588	0.070	HCC2185_BREAST	BRCA
hsa-miR-145-3p	-1.565	0.077	UACC812_BREAST	BRCA
hsa-miR-145-3p	-1.575	0.085	MCF7_BREAST	BRCA
hsa-miR-145-3p	-1.526	0.089	HCC202_BREAST	BRCA
hsa-miR-145-3p	-1.592	0.097	SUM159PT_BREAST	BRCA
hsa-miR-145-3p	-1.581	0.097	AU565_BREAST	BRCA
hsa-miR-145-3p	-1.530	0.0.099401	MDAMB468_BREAST	BRCA
hsa-miR-145-3p	-1.824	0.001	COLO680N_OESOPHAGUS	ESCA
hsa-miR-145-3p	-1.907	0.001	TE1_OESOPHAGUS	ESCA
hsa-miR-145-3p	-1.769	0.010	TE10_OESOPHAGUS	ESCA
hsa-miR-145-3p	-1.662	0.017	TE4_OESOPHAGUS	ESCA
hsa-miR-145-3p	-1.657	0.018	KYSE30_OESOPHAGUS	ESCA
hsa-miR-145-3p	-1.711	0.019	TT_OESOPHAGUS	ESCA
hsa-miR-145-3p	-1.572	0.035	KYSE520_OESOPHAGUS	ESCA
hsa-miR-145-3p	-1.575	0.039	KYSE410_OESOPHAGUS	ESCA
hsa-miR-145-3p	-1.649	0.039	TE8_OESOPHAGUS	ESCA
hsa-miR-145-3p	-1.641	0.047	TE11_OESOPHAGUS	ESCA
hsa-miR-145-3p	-1.690	0.050	ECGI10_OESOPHAGUS	ESCA
hsa-miR-145-3p	-1.559	0.081	JHESOAD1_OESOPHAGUS	ESCA
hsa-miR-145-3p	-1.720	0.014	RERFLCAD2_LUNG	LUAD
hsa-miR-145-3p	-1.767	0.016	NCIH441_LUNG	LUAD
hsa-miR-145-3p	-1.727	0.016	LXF289_LUNG	LUAD
hsa-miR-145-3p	-1.707	0.016	NCIH2009_LUNG	LUAD
hsa-miR-145-3p	-1.686	0.019	NCIH2126_LUNG	LUAD

hsa-miR-145-3p	-1.754	0.026	NCIH3255_LUNG	LUAD
hsa-miR-145-3p	-1.689	0.028	NCIH1437_LUNG	LUAD
hsa-miR-145-3p	-1.784	0.032	HCC461_LUNG	LUAD
hsa-miR-145-3p	-1.656	0.039	NCIH1623_LUNG	LUAD
hsa-miR-145-3p	-1.699	0.040	NCIH2087_LUNG	LUAD
hsa-miR-145-3p	-1.571	0.045	NCIH1568_LUNG	LUAD
hsa-miR-145-3p	-1.615	0.047	HCC827_LUNG	LUAD
hsa-miR-145-3p	-1.607	0.050	NCIH1435_LUNG	LUAD
hsa-miR-145-3p	-1.725	0.053	NCIH2291_LUNG	LUAD
hsa-miR-145-3p	-1.643	0.055	NCIH2122_LUNG	LUAD
hsa-miR-145-3p	-1.643	0.059	NCIH2030_LUNG	LUAD
hsa-miR-145-3p	-1.706	0.066	NCIH3122_LUNG	LUAD
hsa-miR-145-3p	-1.631	0.075	NCIH1373_LUNG	LUAD
hsa-miR-145-3p	-1.647	0.079	NCIH1792_LUNG	LUAD
hsa-miR-145-3p	-1.565	0.084	HCC515_LUNG	LUAD
hsa-miR-145-3p	-1.681	0.084	NCIH1648_LUNG	LUAD
hsa-miR-145-3p	-1.509	0.089	NCIH1944_LUNG	LUAD
hsa-miR-145-3p	-1.554	0.092	HCC4006_LUNG	LUAD
hsa-miR-145-3p	-1.633	0.094	NCIH23_LUNG	LUAD
hsa-miR-145-3p	-1.505	0.095	NCIH854_LUNG	LUAD
hsa-miR-145-3p	-1.900	0.001	SKMES1_LUNG	LUSC
hsa-miR-145-3p	-1.845	0.001	KNS62_LUNG	LUSC
hsa-miR-145-3p	-1.877	0.002	LOUNH91_LUNG	LUSC
hsa-miR-145-3p	-1.963	0.002	CALU1_LUNG	LUSC
hsa-miR-145-3p	-1.859	0.002	EPLC272H_LUNG	LUSC
hsa-miR-145-3p	-1.801	0.004	RERFLCAI_LUNG	LUSC
hsa-miR-145-3p	-1.748	0.005	LUDLU1_LUNG	LUSC
hsa-miR-145-3p	-1.769	0.007	NCIH2882_LUNG	LUSC
hsa-miR-145-3p	-1.819	0.008	LK2_LUNG	LUSC
hsa-miR-145-3p	-1.663	0.009	NCIH2170_LUNG	LUSC
hsa-miR-145-3p	-1.622	0.011	EBC1_LUNG	LUSC
hsa-miR-145-3p	-1.654	0.013	NCIH1703_LUNG	LUSC
hsa-miR-145-3p	-1.729	0.013	HCC15_LUNG	LUSC
hsa-miR-145-3p	-1.648	0.025	HCC2814_LUNG	LUSC
hsa-miR-145-3p	-1.586	0.030	SQ1_LUNG	LUSC
hsa-miR-145-3p	-1.636	0.041	HCC95_LUNG	LUSC
hsa-miR-145-3p	-1.504	0.046	NCIH520_LUNG	LUSC
hsa-miR-145-3p	-1.527	0.061	LC1SQSF_LUNG	LUSC
hsa-miR-145-3p	-1.878	0.001	GSU_STOMACH	STAD
hsa-miR-145-3p	-1.869	0.001	SNU719_STOMACH	STAD
hsa-miR-145-3p	-1.777	0.002	SNU5_STOMACH	STAD
hsa-miR-145-3p	-1.740	0.003	NUGC3_STOMACH	STAD
hsa-miR-145-3p	-1.678	0.003	SNU601_STOMACH	STAD

hsa-miR-145-3p	-1.700	0.005	KE39_STOMACH	STAD
hsa-miR-145-3p	-1.644	0.010	IM95_STOMACH	STAD
hsa-miR-145-3p	-1.615	0.011	AGS_STOMACH	STAD
hsa-miR-145-3p	-1.612	0.013	KATOIII_STOMACH	STAD
hsa-miR-145-3p	-1.586	0.015	2313287_STOMACH	STAD
hsa-miR-145-3p	-1.698	0.015	NCCSTCK140_STOMACH	STAD
hsa-miR-145-3p	-1.582	0.018	OCUM1_STOMACH	STAD
hsa-miR-145-3p	-1.572	0.019	MKN45_STOMACH	STAD
hsa-miR-145-3p	-1.593	0.019	HUG1N_STOMACH	STAD
hsa-miR-145-3p	-1.577	0.023	NCIN87_STOMACH	STAD
hsa-miR-145-3p	-1.586	0.024	MKN1_STOMACH	STAD
hsa-miR-145-3p	-1.589	0.051	ECC10_STOMACH	STAD
hsa-miR-145-5p	-2.004	0.000	HS578T_BREAST	BRCA
hsa-miR-145-5p	-1.994	0.000	MDAMB415_BREAST	BRCA
hsa-miR-145-5p	-1.796	0.001	HCC1419_BREAST	BRCA
hsa-miR-145-5p	-1.812	0.002	MDAMB231_BREAST	BRCA
hsa-miR-145-5p	-1.893	0.002	SKBR5_BREAST	BRCA
hsa-miR-145-5p	-1.811	0.003	MACLS2_BREAST	BRCA
hsa-miR-145-5p	-1.801	0.003	CAMA1_BREAST	BRCA
hsa-miR-145-5p	-1.867	0.003	T47D_BREAST	BRCA
hsa-miR-145-5p	-1.832	0.004	MCF12A_BREAST	BRCA
hsa-miR-145-5p	-1.767	0.006	HCC1428_BREAST	BRCA
hsa-miR-145-5p	-1.958	0.006	184A1_BREAST	BRCA
hsa-miR-145-5p	-1.775	0.006	MDAMB453_BREAST	BRCA
hsa-miR-145-5p	-1.757	0.006	EFM19_BREAST	BRCA
hsa-miR-145-5p	-1.798	0.006	SW527_BREAST	BRCA
hsa-miR-145-5p	-1.778	0.008	CAL51_BREAST	BRCA
hsa-miR-145-5p	-1.745	0.009	ZR751_BREAST	BRCA
hsa-miR-145-5p	-1.710	0.009	MX1_BREAST	BRCA
hsa-miR-145-5p	-1.751	0.009	UACC812_BREAST	BRCA
hsa-miR-145-5p	-1.769	0.012	MDAMB361_BREAST	BRCA
hsa-miR-145-5p	-1.687	0.014	MB157_BREAST	BRCA
hsa-miR-145-5p	-1.860	0.016	SUM159PT_BREAST	BRCA
hsa-miR-145-5p	-1.697	0.016	KPL1_BREAST	BRCA
hsa-miR-145-5p	-1.671	0.018	SUM149PT_BREAST	BRCA
hsa-miR-145-5p	-1.681	0.018	HCC1937_BREAST	BRCA
hsa-miR-145-5p	-1.760	0.019	HCC3153_BREAST	BRCA
hsa-miR-145-5p	-1.742	0.021	EVSAT_BREAST	BRCA
hsa-miR-145-5p	-1.707	0.023	HDQP1_BREAST	BRCA
hsa-miR-145-5p	-1.757	0.024	ZR75B_BREAST	BRCA
hsa-miR-145-5p	-1.654	0.025	MDAMB436_BREAST	BRCA
hsa-miR-145-5p	-1.720	0.031	HCC1395_BREAST	BRCA
hsa-miR-145-5p	-1.603	0.033	HCC1806_BREAST	BRCA

hsa-miR-145-5p	-1.680	0.033	HCC38_BREAST	BRCA
hsa-miR-145-5p	-1.685	0.033	MCF7_BREAST	BRCA
hsa-miR-145-5p	-1.623	0.034	MDAMB468_BREAST	BRCA
hsa-miR-145-5p	-1.689	0.036	MDAMB175VII_BREAST	BRCA
hsa-miR-145-5p	-1.711	0.039	CAL120_BREAST	BRCA
hsa-miR-145-5p	-1.668	0.042	ZR7530_BREAST	BRCA
hsa-miR-145-5p	-1.608	0.043	HCC70_BREAST	BRCA
hsa-miR-145-5p	-1.572	0.044	MDAMB330_BREAST	BRCA
hsa-miR-145-5p	-1.590	0.045	SUM1315MO2_BREAST	BRCA
hsa-miR-145-5p	-1.610	0.052	SUM225CWN_BREAST	BRCA
hsa-miR-145-5p	-1.630	0.052	HCC202_BREAST	BRCA
hsa-miR-145-5p	-1.630	0.054	SKBR7_BREAST	BRCA
hsa-miR-145-5p	-1.612	0.054	BT20_BREAST	BRCA
hsa-miR-145-5p	-1.628	0.056	HCC1500_BREAST	BRCA
hsa-miR-145-5p	-1.578	0.061	BT483_BREAST	BRCA
hsa-miR-145-5p	-1.624	0.065	DU4475_BREAST	BRCA
hsa-miR-145-5p	-1.621	0.065	BT474_BREAST	BRCA
hsa-miR-145-5p	-1.553	0.075	SUM185PE_BREAST	BRCA
hsa-miR-145-5p	-1.648	0.078	AU565_BREAST	BRCA
hsa-miR-145-5p	-1.669	0.078	HCC2185_BREAST	BRCA
hsa-miR-145-5p	-1.580	0.080	CAL148_BREAST	BRCA
hsa-miR-145-5p	-1.584	0.082	SKBR3_BREAST	BRCA
hsa-miR-145-5p	-1.569	0.088	HCC1187_BREAST	BRCA
hsa-miR-145-5p	-1.499	0.088	CAL851_BREAST	BRCA
hsa-miR-145-5p	-1.521	0.091	HCC2218_BREAST	BRCA
hsa-miR-145-5p	-1.595	0.099	MDAMB157_BREAST	BRCA
hsa-miR-145-5p	-1.856	0.001	TE1_OESOPHAGUS	ESCA
hsa-miR-145-5p	-1.783	0.005	TE14_OESOPHAGUS	ESCA
hsa-miR-145-5p	-1.799	0.006	KYSE450_OESOPHAGUS	ESCA
hsa-miR-145-5p	-1.717	0.011	KYSE30_OESOPHAGUS	ESCA
hsa-miR-145-5p	-1.793	0.014	TE8_OESOPHAGUS	ESCA
hsa-miR-145-5p	-1.653	0.016	TE4_OESOPHAGUS	ESCA
hsa-miR-145-5p	-1.712	0.020	TE10_OESOPHAGUS	ESCA
hsa-miR-145-5p	-1.614	0.028	COLO680N_OESOPHAGUS	ESCA
hsa-miR-145-5p	-1.610	0.028	KYSE520_OESOPHAGUS	ESCA
hsa-miR-145-5p	-1.679	0.042	OE33_OESOPHAGUS	ESCA
hsa-miR-145-5p	-1.666	0.045	TE11_OESOPHAGUS	ESCA
hsa-miR-145-5p	-1.608	0.045	KYSE140_OESOPHAGUS	ESCA
hsa-miR-145-5p	-1.626	0.054	KYSE150_OESOPHAGUS	ESCA
hsa-miR-145-5p	-1.656	0.076	KYSE510_OESOPHAGUS	ESCA
hsa-miR-145-5p	-1.636	0.078	KYSE180_OESOPHAGUS	ESCA
hsa-miR-145-5p	-1.721	0.080	TE15_OESOPHAGUS	ESCA
hsa-miR-145-5p	-1.544	0.096	KYSE70_OESOPHAGUS	ESCA

hsa-miR-145-5p	-1.676	0.045	JHH1_LIVER	LIHC
hsa-miR-145-5p	-1.649	0.029	NCIH2126_LUNG	LUAD
hsa-miR-145-5p	-1.658	0.039	NCIH854_LUNG	LUAD
hsa-miR-145-5p	-1.601	0.039	NCIH1623_LUNG	LUAD
hsa-miR-145-5p	-1.629	0.040	NCIH1435_LUNG	LUAD
hsa-miR-145-5p	-1.551	0.043	NCIH1568_LUNG	LUAD
hsa-miR-145-5p	-1.568	0.046	RERFLCMS_LUNG	LUAD
hsa-miR-145-5p	-1.589	0.046	RERFLCAD2_LUNG	LUAD
hsa-miR-145-5p	-1.573	0.059	HCC827_LUNG	LUAD
hsa-miR-145-5p	-1.561	0.059	LXF289_LUNG	LUAD
hsa-miR-145-5p	-1.627	0.060	HCC4006_LUNG	LUAD
hsa-miR-145-5p	-1.543	0.091	NCIH2291_LUNG	LUAD
hsa-miR-145-5p	-1.563	0.092	NCIH1792_LUNG	LUAD
hsa-miR-145-5p	-1.959	0.001	SKMES1_LUNG	LUSC
hsa-miR-145-5p	-1.835	0.001	KNS62_LUNG	LUSC
hsa-miR-145-5p	-1.978	0.001	LOUNH91_LUNG	LUSC
hsa-miR-145-5p	-1.944	0.002	RERFLCAI_LUNG	LUSC
hsa-miR-145-5p	-1.777	0.003	EPLC272H_LUNG	LUSC
hsa-miR-145-5p	-1.843	0.003	EBC1_LUNG	LUSC
hsa-miR-145-5p	-1.724	0.006	NCIH2170_LUNG	LUSC
hsa-miR-145-5p	-1.721	0.006	CALU1_LUNG	LUSC
hsa-miR-145-5p	-1.719	0.007	NCIH1703_LUNG	LUSC
hsa-miR-145-5p	-1.775	0.008	NCIH2882_LUNG	LUSC
hsa-miR-145-5p	-1.786	0.008	LK2_LUNG	LUSC
hsa-miR-145-5p	-1.718	0.011	HCC15_LUNG	LUSC
hsa-miR-145-5p	-1.615	0.015	LUDLU1_LUNG	LUSC
hsa-miR-145-5p	-1.697	0.019	HCC2814_LUNG	LUSC
hsa-miR-145-5p	-1.636	0.036	HCC95_LUNG	LUSC
hsa-miR-145-5p	-1.543	0.045	SQ1_LUNG	LUSC
hsa-miR-145-5p	-1.641	0.046	NCIH520_LUNG	LUSC
hsa-miR-145-5p	-1.607	0.059	LC1SQSF_LUNG	LUSC
hsa-miR-145-5p	-1.822	0.001	GSU_STOMACH	STAD
hsa-miR-145-5p	-1.831	0.001	SNU5_STOMACH	STAD
hsa-miR-145-5p	-1.782	0.002	MKN45_STOMACH	STAD
hsa-miR-145-5p	-1.709	0.003	AGS_STOMACH	STAD
hsa-miR-145-5p	-1.741	0.003	SNU719_STOMACH	STAD
hsa-miR-145-5p	-1.699	0.005	IM95_STOMACH	STAD
hsa-miR-145-5p	-1.733	0.005	HUG1N_STOMACH	STAD
hsa-miR-145-5p	-1.718	0.006	NCIN87_STOMACH	STAD
hsa-miR-145-5p	-1.672	0.006	KE39_STOMACH	STAD
hsa-miR-145-5p	-1.706	0.006	MKN1_STOMACH	STAD
hsa-miR-145-5p	-1.616	0.007	SNU601_STOMACH	STAD
hsa-miR-145-5p	-1.666	0.008	OCUM1_STOMACH	STAD

hsa-miR-145-5p	-1.683	0.014	SNU216_STOMACH	STAD
hsa-miR-145-5p	-1.580	0.015	KATOIII_STOMACH	STAD
hsa-miR-145-5p	-1.541	0.022	2313287_STOMACH	STAD
hsa-miR-145-5p	-1.707	0.023	ECC10_STOMACH	STAD
hsa-miR-145-5p	-1.577	0.024	NUGC3_STOMACH	STAD
hsa-miR-145-5p	-1.602	0.024	NCCSTCK140_STOMACH	STAD
hsa-miR-145-5p	-1.573	0.038	GSS_STOMACH	STAD
hsa-miR-145-5p	-1.604	0.046	MKN7_STOMACH	STAD
hsa-miR-145-5p	-1.636	0.059	FU97_STOMACH	STAD

[$]FDR (false discovery rate) adjusted gene set enrichment *P*-value.

Supplementary Table 4. miRNA whose down-regulation potentially induce survival/growth of cancer cell lines screened by CRISPR

miRNA	Normalized Enrichment Score	FDR$	Cell line	Cancer type
hsa-miR-28-3p	-1.689	0.000	SNU216_STOMACH	STAD
hsa-miR-28-3p	-1.623	0.001	GSU_STOMACH	STAD
hsa-miR-28-3p	-1.598	0.001	MKN74_STOMACH	STAD
hsa-miR-28-3p	-1.634	0.001	SH10TC_STOMACH	STAD
hsa-miR-28-3p	-1.594	0.001	HS746T_STOMACH	STAD
hsa-miR-28-3p	-1.592	0.002	GSS_STOMACH	STAD
hsa-miR-28-3p	-1.586	0.002	LMSU_STOMACH	STAD
hsa-miR-28-3p	-1.604	0.002	HGC27_STOMACH	STAD
hsa-miR-28-3p	-1.576	0.002	AGS_STOMACH	STAD
hsa-miR-28-3p	-1.549	0.002	SNU719_STOMACH	STAD
hsa-miR-28-3p	-1.554	0.003	2313287_STOMACH	STAD
hsa-miR-28-3p	-1.555	0.003	MKN45_STOMACH	STAD
hsa-miR-28-3p	-1.499	0.004	SNU1_STOMACH	STAD
hsa-miR-28-3p	-1.514	0.004	KE39_STOMACH	STAD
hsa-miR-28-3p	-1.488	0.005	NUGC3_STOMACH	STAD
hsa-miR-28-3p	-1.477	0.005	GCIY_STOMACH	STAD
hsa-miR-28-3p	-1.483	0.006	SNU601_STOMACH	STAD
hsa-miR-28-3p	-1.506	0.008	NCIN87_STOMACH	STAD
hsa-miR-28-3p	-1.389	0.020	FU97_STOMACH	STAD
hsa-miR-28-5p	-1.661	0.000	GSU_STOMACH	STAD
hsa-miR-28-5p	-1.632	0.001	GSS_STOMACH	STAD
hsa-miR-28-5p	-1.595	0.001	SNU1_STOMACH	STAD
hsa-miR-28-5p	-1.623	0.001	SH10TC_STOMACH	STAD
hsa-miR-28-5p	-1.578	0.002	SNU216_STOMACH	STAD
hsa-miR-28-5p	-1.550	0.002	SNU719_STOMACH	STAD
hsa-miR-28-5p	-1.531	0.003	MKN74_STOMACH	STAD
hsa-miR-28-5p	-1.596	0.003	NCIN87_STOMACH	STAD
hsa-miR-28-5p	-1.537	0.003	HS746T_STOMACH	STAD
hsa-miR-28-5p	-1.567	0.003	HGC27_STOMACH	STAD
hsa-miR-28-5p	-1.520	0.003	2313287_STOMACH	STAD
hsa-miR-28-5p	-1.549	0.003	AGS_STOMACH	STAD
hsa-miR-28-5p	-1.538	0.004	MKN45_STOMACH	STAD
hsa-miR-28-5p	-1.514	0.004	SNU601_STOMACH	STAD
hsa-miR-28-5p	-1.494	0.005	NUGC3_STOMACH	STAD
hsa-miR-28-5p	-1.516	0.005	LMSU_STOMACH	STAD
hsa-miR-28-5p	-1.469	0.005	GCIY_STOMACH	STAD
hsa-miR-28-5p	-1.467	0.007	KE39_STOMACH	STAD
hsa-miR-28-5p	-1.411	0.017	FU97_STOMACH	STAD
hsa-miR-30a-3p	-1.598	0.002	KYSE410_OESOPHAGUS	ESCA

hsa-miR-30a-3p	-1.605	0.003	TE5_OESOPHAGUS	ESCA
hsa-miR-30a-3p	-1.555	0.006	KYSE180_OESOPHAGUS	ESCA
hsa-miR-30a-3p	-1.525	0.007	TE1_OESOPHAGUS	ESCA
hsa-miR-30a-3p	-1.510	0.009	KYSE70_OESOPHAGUS	ESCA
hsa-miR-30a-3p	-1.511	0.010	OE33_OESOPHAGUS	ESCA
hsa-miR-30a-3p	-1.517	0.018	KYSE150_OESOPHAGUS	ESCA
hsa-miR-30a-3p	-1.484	0.019	KYSE270_OESOPHAGUS	ESCA
hsa-miR-30a-3p	-1.479	0.020	KYSE510_OESOPHAGUS	ESCA
hsa-miR-30a-3p	-1.445	0.020	KYSE30_OESOPHAGUS	ESCA
hsa-miR-30a-3p	-1.462	0.021	OE21_OESOPHAGUS	ESCA
hsa-miR-30a-3p	-1.462	0.022	TE10_OESOPHAGUS	ESCA
hsa-miR-30a-3p	-1.443	0.022	KYSE140_OESOPHAGUS	ESCA
hsa-miR-30a-3p	-1.454	0.024	TE11_OESOPHAGUS	ESCA
hsa-miR-30a-3p	-1.463	0.034	TE6_OESOPHAGUS	ESCA
hsa-miR-30a-3p	-1.418	0.035	TE9_OESOPHAGUS	ESCA
hsa-miR-30a-3p	-1.398	0.040	KYSE450_OESOPHAGUS	ESCA
hsa-miR-30a-3p	-1.413	0.040	OACM51_OESOPHAGUS	ESCA
hsa-miR-30a-3p	-1.439	0.047	SKGT4_OESOPHAGUS	ESCA
hsa-miR-30a-3p	-1.439	0.048	TE4_OESOPHAGUS	ESCA
hsa-miR-30a-3p	-1.418	0.052	FLO1_OESOPHAGUS	ESCA
hsa-miR-30a-3p	-1.371	0.066	TE8_OESOPHAGUS	ESCA
hsa-miR-30a-3p	-1.618	0.008	JHH1_LIVER	LIHC
hsa-miR-30a-3p	-1.634	0.016	SKHEP1_LIVER	LIHC
hsa-miR-30a-3p	-1.565	0.017	SNU886_LIVER	LIHC
hsa-miR-30a-3p	-1.569	0.019	SNU398_LIVER	LIHC
hsa-miR-30a-3p	-1.555	0.023	HUH6_LIVER	LIHC
hsa-miR-30a-3p	-1.542	0.023	SNU449_LIVER	LIHC
hsa-miR-30a-3p	-1.524	0.035	HUH7_LIVER	LIHC
hsa-miR-30a-3p	-1.533	0.045	SNU182_LIVER	LIHC
hsa-miR-30a-3p	-1.543	0.048	JHH7_LIVER	LIHC
hsa-miR-30a-3p	-1.465	0.057	HLF_LIVER	LIHC
hsa-miR-30a-3p	-1.478	0.061	PLCPRF5_LIVER	LIHC
hsa-miR-30a-3p	-1.456	0.066	HUH1_LIVER	LIHC
hsa-miR-30a-3p	-1.443	0.067	JHH5_LIVER	LIHC
hsa-miR-30a-3p	-1.440	0.071	SNU761_LIVER	LIHC
hsa-miR-30a-3p	-1.440	0.099	JHH4_LIVER	LIHC
hsa-miR-30a-3p	-1.514	0.011	NCIH2126_LUNG	LUAD
hsa-miR-30a-3p	-1.528	0.012	NCIH2087_LUNG	LUAD
hsa-miR-30a-3p	-1.524	0.012	RERFLCAD1_LUNG	LUAD
hsa-miR-30a-3p	-1.507	0.013	NCIH441_LUNG	LUAD
hsa-miR-30a-3p	-1.502	0.013	NCIH838_LUNG	LUAD
hsa-miR-30a-3p	-1.519	0.013	NCIH23_LUNG	LUAD
hsa-miR-30a-3p	-1.493	0.015	NCIH2122_LUNG	LUAD

hsa-miR-30a-3p	-1.499	0.016	PC14_LUNG	LUAD
hsa-miR-30a-3p	-1.489	0.022	HCC461_LUNG	LUAD
hsa-miR-30a-3p	-1.487	0.022	HCC2450_LUNG	LUAD
hsa-miR-30a-3p	-1.478	0.023	MORCPR_LUNG	LUAD
hsa-miR-30a-3p	-1.494	0.025	A549_LUNG	LUAD
hsa-miR-30a-3p	-1.486	0.025	NCIH2030_LUNG	LUAD
hsa-miR-30a-3p	-1.518	0.025	HCC515_LUNG	LUAD
hsa-miR-30a-3p	-1.457	0.027	NCIH2023_LUNG	LUAD
hsa-miR-30a-3p	-1.445	0.029	NCIH1437_LUNG	LUAD
hsa-miR-30a-3p	-1.481	0.031	A427_LUNG	LUAD
hsa-miR-30a-3p	-1.493	0.032	HCC2429_LUNG	LUAD
hsa-miR-30a-3p	-1.497	0.036	NCIH1693_LUNG	LUAD
hsa-miR-30a-3p	-1.487	0.037	HOP62_LUNG	LUAD
hsa-miR-30a-3p	-1.452	0.038	HCC2935_LUNG	LUAD
hsa-miR-30a-3p	-1.455	0.039	NCIH1568_LUNG	LUAD
hsa-miR-30a-3p	-1.456	0.039	NCIH3122_LUNG	LUAD
hsa-miR-30a-3p	-1.461	0.039	EKVX_LUNG	LUAD
hsa-miR-30a-3p	-1.442	0.040	NCIH1944_LUNG	LUAD
hsa-miR-30a-3p	-1.424	0.040	ABC1_LUNG	LUAD
hsa-miR-30a-3p	-1.428	0.042	LXF289_LUNG	LUAD
hsa-miR-30a-3p	-1.418	0.047	HCC827_LUNG	LUAD
hsa-miR-30a-3p	-1.430	0.048	NCIH1793_LUNG	LUAD
hsa-miR-30a-3p	-1.420	0.049	NCIH1792_LUNG	LUAD
hsa-miR-30a-3p	-1.515	0.055	NCIH1573_LUNG	LUAD
hsa-miR-30a-3p	-1.407	0.064	NCIH1648_LUNG	LUAD
hsa-miR-30a-3p	-1.425	0.066	NCIH2291_LUNG	LUAD
hsa-miR-30a-3p	-1.761	0.000	HCC15_LUNG	LUSC
hsa-miR-30a-3p	-1.678	0.001	LK2_LUNG	LUSC
hsa-miR-30a-3p	-1.613	0.001	CALU1_LUNG	LUSC
hsa-miR-30a-3p	-1.595	0.001	KNS62_LUNG	LUSC
hsa-miR-30a-3p	-1.596	0.002	NCIH2170_LUNG	LUSC
hsa-miR-30a-3p	-1.596	0.002	HARA_LUNG	LUSC
hsa-miR-30a-3p	-1.609	0.002	NCIH520_LUNG	LUSC
hsa-miR-30a-3p	-1.574	0.002	NCIH1703_LUNG	LUSC
hsa-miR-30a-3p	-1.546	0.002	EPLC272H_LUNG	LUSC
hsa-miR-30a-3p	-1.555	0.002	LUDLU1_LUNG	LUSC
hsa-miR-30a-3p	-1.597	0.003	HCC95_LUNG	LUSC
hsa-miR-30a-3p	-1.551	0.003	SKMES1_LUNG	LUSC
hsa-miR-30a-3p	-1.518	0.004	RERFLCAI_LUNG	LUSC
hsa-miR-30a-3p	-1.665	0.000	KE39_STOMACH	STAD
hsa-miR-30a-3p	-1.780	0.000	SH10TC_STOMACH	STAD
hsa-miR-30a-3p	-1.657	0.000	AGS_STOMACH	STAD
hsa-miR-30a-3p	-1.727	0.000	GSS_STOMACH	STAD

hsa-miR-30a-3p	-1.701	0.000	MKN45_STOMACH	STAD
hsa-miR-30a-3p	-1.689	0.001	GSU_STOMACH	STAD
hsa-miR-30a-3p	-1.663	0.001	MKN74_STOMACH	STAD
hsa-miR-30a-3p	-1.634	0.001	SNU719_STOMACH	STAD
hsa-miR-30a-3p	-1.633	0.001	GCIY_STOMACH	STAD
hsa-miR-30a-3p	-1.623	0.001	SNU1_STOMACH	STAD
hsa-miR-30a-3p	-1.623	0.001	SNU216_STOMACH	STAD
hsa-miR-30a-3p	-1.618	0.001	HS746T_STOMACH	STAD
hsa-miR-30a-3p	-1.671	0.001	HGC27_STOMACH	STAD
hsa-miR-30a-3p	-1.586	0.001	SNU601_STOMACH	STAD
hsa-miR-30a-3p	-1.627	0.002	NCIN87_STOMACH	STAD
hsa-miR-30a-3p	-1.617	0.002	LMSU_STOMACH	STAD
hsa-miR-30a-3p	-1.589	0.003	2313287_STOMACH	STAD
hsa-miR-30a-3p	-1.554	0.003	NUGC3_STOMACH	STAD
hsa-miR-30a-3p	-1.564	0.003	FU97_STOMACH	STAD
hsa-miR-30a-5p	-1.635	0.004	RT112_URINARY_TRACT	BLCA
hsa-miR-30a-5p	-1.679	0.004	SCABER_URINARY_TRACT	BLCA
hsa-miR-30a-5p	-1.652	0.004	639V_URINARY_TRACT	BLCA
hsa-miR-30a-5p	-1.624	0.005	253J_URINARY_TRACT	BLCA
hsa-miR-30a-5p	-1.576	0.005	UMUC1_URINARY_TRACT	BLCA
hsa-miR-30a-5p	-1.585	0.005	CAL29_URINARY_TRACT	BLCA
hsa-miR-30a-5p	-1.690	0.007	HT1376_URINARY_TRACT	BLCA
hsa-miR-30a-5p	-1.607	0.008	RT11284_URINARY_TRACT	BLCA
hsa-miR-30a-5p	-1.619	0.009	HT1197_URINARY_TRACT	BLCA
hsa-miR-30a-5p	-1.616	0.009	5637_URINARY_TRACT	BLCA
hsa-miR-30a-5p	-1.584	0.010	UMUC11_URINARY_TRACT	BLCA
hsa-miR-30a-5p	-1.546	0.011	UMUC13_URINARY_TRACT	BLCA
hsa-miR-30a-5p	-1.563	0.014	UMUC14_URINARY_TRACT	BLCA
hsa-miR-30a-5p	-1.545	0.017	KMBC2_URINARY_TRACT	BLCA
hsa-miR-30a-5p	-1.528	0.018	KU1919_URINARY_TRACT	BLCA
hsa-miR-30a-5p	-1.515	0.023	UMUC3_URINARY_TRACT	BLCA
hsa-miR-30a-5p	-1.538	0.027	T24_URINARY_TRACT	BLCA
hsa-miR-30a-5p	-1.527	0.027	BC3C_URINARY_TRACT	BLCA
hsa-miR-30a-5p	-1.516	0.028	VMCUB1_URINARY_TRACT	BLCA
hsa-miR-30a-5p	-1.499	0.030	JMSU1_URINARY_TRACT	BLCA
hsa-miR-30a-5p	-1.506	0.040	TCCSUP_URINARY_TRACT	BLCA
hsa-miR-30a-5p	-1.498	0.043	BFTC905_URINARY_TRACT	BLCA
hsa-miR-30a-5p	-1.667	0.001	KYSE270_OESOPHAGUS	ESCA
hsa-miR-30a-5p	-1.613	0.001	KYSE410_OESOPHAGUS	ESCA
hsa-miR-30a-5p	-1.691	0.001	KYSE150_OESOPHAGUS	ESCA
hsa-miR-30a-5p	-1.611	0.003	KYSE450_OESOPHAGUS	ESCA
hsa-miR-30a-5p	-1.618	0.003	TE5_OESOPHAGUS	ESCA
hsa-miR-30a-5p	-1.619	0.003	OE21_OESOPHAGUS	ESCA

hsa-miR-30a-5p	-1.632	0.004	TE10_OESOPHAGUS	ESCA
hsa-miR-30a-5p	-1.556	0.004	OE33_OESOPHAGUS	ESCA
hsa-miR-30a-5p	-1.566	0.005	KYSE520_OESOPHAGUS	ESCA
hsa-miR-30a-5p	-1.579	0.005	TE11_OESOPHAGUS	ESCA
hsa-miR-30a-5p	-1.534	0.006	KYSE30_OESOPHAGUS	ESCA
hsa-miR-30a-5p	-1.537	0.007	TE1_OESOPHAGUS	ESCA
hsa-miR-30a-5p	-1.559	0.008	KYSE180_OESOPHAGUS	ESCA
hsa-miR-30a-5p	-1.523	0.008	KYSE70_OESOPHAGUS	ESCA
hsa-miR-30a-5p	-1.509	0.009	KYSE140_OESOPHAGUS	ESCA
hsa-miR-30a-5p	-1.534	0.009	TE9_OESOPHAGUS	ESCA
hsa-miR-30a-5p	-1.529	0.014	KYSE510_OESOPHAGUS	ESCA
hsa-miR-30a-5p	-1.501	0.016	TE8_OESOPHAGUS	ESCA
hsa-miR-30a-5p	-1.492	0.019	ESO26_OESOPHAGUS	ESCA
hsa-miR-30a-5p	-1.522	0.023	TE6_OESOPHAGUS	ESCA
hsa-miR-30a-5p	-1.467	0.024	OACM51_OESOPHAGUS	ESCA
hsa-miR-30a-5p	-1.508	0.028	TE4_OESOPHAGUS	ESCA
hsa-miR-30a-5p	-1.445	0.039	FLO1_OESOPHAGUS	ESCA
hsa-miR-30a-5p	-1.432	0.041	SKGT4_OESOPHAGUS	ESCA
hsa-miR-30a-5p	-1.616	0.002	MORCPR_LUNG	LUAD
hsa-miR-30a-5p	-1.661	0.003	NCIH441_LUNG	LUAD
hsa-miR-30a-5p	-1.638	0.003	NCIH2126_LUNG	LUAD
hsa-miR-30a-5p	-1.595	0.004	PC14_LUNG	LUAD
hsa-miR-30a-5p	-1.604	0.005	NCIH2122_LUNG	LUAD
hsa-miR-30a-5p	-1.566	0.006	NCIH2023_LUNG	LUAD
hsa-miR-30a-5p	-1.560	0.011	NCIH2087_LUNG	LUAD
hsa-miR-30a-5p	-1.531	0.013	RERFLCAD1_LUNG	LUAD
hsa-miR-30a-5p	-1.528	0.014	HCC461_LUNG	LUAD
hsa-miR-30a-5p	-1.491	0.014	NCIH838_LUNG	LUAD
hsa-miR-30a-5p	-1.487	0.026	NCIH1568_LUNG	LUAD
hsa-miR-30a-5p	-1.454	0.027	A549_LUNG	LUAD
hsa-miR-30a-5p	-1.481	0.028	NCIH1792_LUNG	LUAD
hsa-miR-30a-5p	-1.507	0.028	NCIH3122_LUNG	LUAD
hsa-miR-30a-5p	-1.471	0.029	HCC827_LUNG	LUAD
hsa-miR-30a-5p	-1.447	0.029	HCC2450_LUNG	LUAD
hsa-miR-30a-5p	-1.450	0.032	NCIH1437_LUNG	LUAD
hsa-miR-30a-5p	-1.485	0.033	HOP62_LUNG	LUAD
hsa-miR-30a-5p	-1.488	0.035	NCIH2291_LUNG	LUAD
hsa-miR-30a-5p	-1.445	0.036	HCC2935_LUNG	LUAD
hsa-miR-30a-5p	-1.494	0.036	HCC2429_LUNG	LUAD
hsa-miR-30a-5p	-1.434	0.038	NCIH1944_LUNG	LUAD
hsa-miR-30a-5p	-1.420	0.039	ABC1_LUNG	LUAD
hsa-miR-30a-5p	-1.437	0.039	LXF289_LUNG	LUAD
hsa-miR-30a-5p	-1.425	0.041	NCIH2030_LUNG	LUAD

hsa-miR-30a-5p	-1.458	0.042	NCIH1793_LUNG	LUAD
hsa-miR-30a-5p	-1.450	0.043	NCIH1648_LUNG	LUAD
hsa-miR-30a-5p	-1.407	0.044	NCIH23_LUNG	LUAD
hsa-miR-30a-5p	-1.502	0.044	EKVX_LUNG	LUAD
hsa-miR-30a-5p	-1.433	0.047	A427_LUNG	LUAD
hsa-miR-30a-5p	-1.427	0.049	HCC515_LUNG	LUAD
hsa-miR-30a-5p	-1.404	0.073	NCIH1693_LUNG	LUAD
hsa-miR-30a-5p	-1.432	0.084	NCIH1573_LUNG	LUAD
hsa-miR-30a-5p	-1.704	0.001	HCC15_LUNG	LUSC
hsa-miR-30a-5p	-1.673	0.001	HARA_LUNG	LUSC
hsa-miR-30a-5p	-1.585	0.001	LK2_LUNG	LUSC
hsa-miR-30a-5p	-1.643	0.001	HCC95_LUNG	LUSC
hsa-miR-30a-5p	-1.632	0.001	NCIH520_LUNG	LUSC
hsa-miR-30a-5p	-1.577	0.002	KNS62_LUNG	LUSC
hsa-miR-30a-5p	-1.580	0.002	LUDLU1_LUNG	LUSC
hsa-miR-30a-5p	-1.637	0.002	NCIH2170_LUNG	LUSC
hsa-miR-30a-5p	-1.560	0.002	EPLC272H_LUNG	LUSC
hsa-miR-30a-5p	-1.569	0.002	CALU1_LUNG	LUSC
hsa-miR-30a-5p	-1.589	0.002	SKMES1_LUNG	LUSC
hsa-miR-30a-5p	-1.546	0.003	RERFLCAI_LUNG	LUSC
hsa-miR-30a-5p	-1.513	0.004	NCIH1703_LUNG	LUSC
hsa-miR-30a-5p	-1.740	0.000	GSS_STOMACH	STAD
hsa-miR-30a-5p	-1.643	0.000	GSU_STOMACH	STAD
hsa-miR-30a-5p	-1.649	0.000	MKN74_STOMACH	STAD
hsa-miR-30a-5p	-1.623	0.001	HS746T_STOMACH	STAD
hsa-miR-30a-5p	-1.613	0.001	NUGC3_STOMACH	STAD
hsa-miR-30a-5p	-1.600	0.001	SNU601_STOMACH	STAD
hsa-miR-30a-5p	-1.591	0.002	SNU216_STOMACH	STAD
hsa-miR-30a-5p	-1.544	0.002	GCIY_STOMACH	STAD
hsa-miR-30a-5p	-1.543	0.002	SNU719_STOMACH	STAD
hsa-miR-30a-5p	-1.587	0.002	LMSU_STOMACH	STAD
hsa-miR-30a-5p	-1.579	0.002	MKN45_STOMACH	STAD
hsa-miR-30a-5p	-1.566	0.003	SH10TC_STOMACH	STAD
hsa-miR-30a-5p	-1.532	0.003	SNU1_STOMACH	STAD
hsa-miR-30a-5p	-1.526	0.003	2313287_STOMACH	STAD
hsa-miR-30a-5p	-1.531	0.003	AGS_STOMACH	STAD
hsa-miR-30a-5p	-1.548	0.004	HGC27_STOMACH	STAD
hsa-miR-30a-5p	-1.485	0.006	KE39_STOMACH	STAD
hsa-miR-30a-5p	-1.485	0.010	NCIN87_STOMACH	STAD
hsa-miR-30a-5p	-1.400	0.018	FU97_STOMACH	STAD
hsa-miR-139-3p	-1.471	0.022	TE10_OESOPHAGUS	ESCA
hsa-miR-139-3p	-1.371	0.054	OE33_OESOPHAGUS	ESCA
hsa-miR-139-3p	-1.353	0.065	KYSE410_OESOPHAGUS	ESCA

hsa-miR-139-3p	-1.333	0.065	TE1_OESOPHAGUS	ESCA
hsa-miR-139-3p	-1.377	0.075	TE6_OESOPHAGUS	ESCA
hsa-miR-139-3p	-1.473	0.071	SKHEP1_LIVER	LIHC
hsa-miR-139-5p	-1.772	0.001	T24_URINARY_TRACT	BLCA
hsa-miR-139-5p	-1.672	0.001	VMCUB1_URINARY_TRACT	BLCA
hsa-miR-139-5p	-1.687	0.003	UMUC13_URINARY_TRACT	BLCA
hsa-miR-139-5p	-1.653	0.003	253J_URINARY_TRACT	BLCA
hsa-miR-139-5p	-1.650	0.003	UMUC3_URINARY_TRACT	BLCA
hsa-miR-139-5p	-1.672	0.003	RT112_URINARY_TRACT	BLCA
hsa-miR-139-5p	-1.620	0.004	UMUC1_URINARY_TRACT	BLCA
hsa-miR-139-5p	-1.652	0.005	SCABER_URINARY_TRACT	BLCA
hsa-miR-139-5p	-1.617	0.005	639V_URINARY_TRACT	BLCA
hsa-miR-139-5p	-1.585	0.007	CAL29_URINARY_TRACT	BLCA
hsa-miR-139-5p	-1.610	0.008	HT1197_URINARY_TRACT	BLCA
hsa-miR-139-5p	-1.587	0.009	RT11284_URINARY_TRACT	BLCA
hsa-miR-139-5p	-1.568	0.015	KU1919_URINARY_TRACT	BLCA
hsa-miR-139-5p	-1.522	0.016	UMUC14_URINARY_TRACT	BLCA
hsa-miR-139-5p	-1.528	0.019	5637_URINARY_TRACT	BLCA
hsa-miR-139-5p	-1.549	0.021	KMBC2_URINARY_TRACT	BLCA
hsa-miR-139-5p	-1.489	0.032	UMUC11_URINARY_TRACT	BLCA
hsa-miR-139-5p	-1.507	0.035	JMSU1_URINARY_TRACT	BLCA
hsa-miR-139-5p	-1.475	0.037	BC3C_URINARY_TRACT	BLCA
hsa-miR-139-5p	-1.467	0.043	TCCSUP_URINARY_TRACT	BLCA
hsa-miR-139-5p	-1.513	0.066	BFTC905_URINARY_TRACT	BLCA
hsa-miR-139-5p	-1.454	0.074	HT1376_URINARY_TRACT	BLCA
hsa-miR-139-5p	-1.453	0.081	647V_URINARY_TRACT	BLCA
hsa-miR-139-5p	-1.850	0.000	DU4475_BREAST	BRCA
hsa-miR-139-5p	-1.846	0.000	HCC1954_BREAST	BRCA
hsa-miR-139-5p	-1.793	0.000	AU565_BREAST	BRCA
hsa-miR-139-5p	-1.781	0.000	KPL1_BREAST	BRCA
hsa-miR-139-5p	-1.746	0.000	MDAMB468_BREAST	BRCA
hsa-miR-139-5p	-1.671	0.000	ZR751_BREAST	BRCA
hsa-miR-139-5p	-1.704	0.000	EFM19_BREAST	BRCA
hsa-miR-139-5p	-1.758	0.000	SUM159PT_BREAST	BRCA
hsa-miR-139-5p	-1.702	0.000	MDAMB231_BREAST	BRCA
hsa-miR-139-5p	-1.694	0.001	HCC1428_BREAST	BRCA
hsa-miR-139-5p	-1.732	0.001	CAMA1_BREAST	BRCA
hsa-miR-139-5p	-1.808	0.001	HCC1419_BREAST	BRCA
hsa-miR-139-5p	-1.731	0.001	MCF7_BREAST	BRCA
hsa-miR-139-5p	-1.676	0.001	HS578T_BREAST	BRCA
hsa-miR-139-5p	-1.705	0.001	HCC1806_BREAST	BRCA
hsa-miR-139-5p	-1.666	0.001	JIMT1_BREAST	BRCA
hsa-miR-139-5p	-1.673	0.001	MDAMB436_BREAST	BRCA

hsa-miR-139-5p	-1.628	0.001	HCC1937_BREAST	BRCA
hsa-miR-139-5p	-1.632	0.002	HMC18_BREAST	BRCA
hsa-miR-139-5p	-1.670	0.002	MDAMB453_BREAST	BRCA
hsa-miR-139-5p	-1.644	0.002	HCC1143_BREAST	BRCA
hsa-miR-139-5p	-1.635	0.002	MDAMB157_BREAST	BRCA
hsa-miR-139-5p	-1.630	0.002	HCC1395_BREAST	BRCA
hsa-miR-139-5p	-1.639	0.002	CAL51_BREAST	BRCA
hsa-miR-139-5p	-1.624	0.003	SKBR3_BREAST	BRCA
hsa-miR-139-5p	-1.654	0.003	HCC202_BREAST	BRCA
hsa-miR-139-5p	-1.632	0.003	MDAMB415_BREAST	BRCA
hsa-miR-139-5p	-1.629	0.003	BT549_BREAST	BRCA
hsa-miR-139-5p	-1.724	0.001	KYSE270_OESOPHAGUS	ESCA
hsa-miR-139-5p	-1.643	0.001	KYSE410_OESOPHAGUS	ESCA
hsa-miR-139-5p	-1.645	0.001	TE1_OESOPHAGUS	ESCA
hsa-miR-139-5p	-1.727	0.001	TE5_OESOPHAGUS	ESCA
hsa-miR-139-5p	-1.604	0.002	KYSE450_OESOPHAGUS	ESCA
hsa-miR-139-5p	-1.589	0.002	KYSE30_OESOPHAGUS	ESCA
hsa-miR-139-5p	-1.629	0.002	KYSE520_OESOPHAGUS	ESCA
hsa-miR-139-5p	-1.650	0.003	KYSE150_OESOPHAGUS	ESCA
hsa-miR-139-5p	-1.632	0.003	TE10_OESOPHAGUS	ESCA
hsa-miR-139-5p	-1.610	0.003	KYSE140_OESOPHAGUS	ESCA
hsa-miR-139-5p	-1.562	0.004	OE33_OESOPHAGUS	ESCA
hsa-miR-139-5p	-1.588	0.004	KYSE70_OESOPHAGUS	ESCA
hsa-miR-139-5p	-1.584	0.004	SKGT4_OESOPHAGUS	ESCA
hsa-miR-139-5p	-1.610	0.005	TE11_OESOPHAGUS	ESCA
hsa-miR-139-5p	-1.579	0.006	OACM51_OESOPHAGUS	ESCA
hsa-miR-139-5p	-1.601	0.007	TE4_OESOPHAGUS	ESCA
hsa-miR-139-5p	-1.563	0.008	TE8_OESOPHAGUS	ESCA
hsa-miR-139-5p	-1.519	0.010	TE9_OESOPHAGUS	ESCA
hsa-miR-139-5p	-1.538	0.012	ESO26_OESOPHAGUS	ESCA
hsa-miR-139-5p	-1.563	0.013	KYSE510_OESOPHAGUS	ESCA
hsa-miR-139-5p	-1.502	0.014	KYSE180_OESOPHAGUS	ESCA
hsa-miR-139-5p	-1.517	0.018	TE6_OESOPHAGUS	ESCA
hsa-miR-139-5p	-1.532	0.019	FLO1_OESOPHAGUS	ESCA
hsa-miR-139-5p	-1.415	0.033	OE21_OESOPHAGUS	ESCA
hsa-miR-139-5p	-1.577	0.018	PLCPRF5_LIVER	LIHC
hsa-miR-139-5p	-1.592	0.019	SNU449_LIVER	LIHC
hsa-miR-139-5p	-1.565	0.022	SNU886_LIVER	LIHC
hsa-miR-139-5p	-1.583	0.024	SKHEP1_LIVER	LIHC
hsa-miR-139-5p	-1.491	0.034	SNU398_LIVER	LIHC
hsa-miR-139-5p	-1.453	0.050	JHH1_LIVER	LIHC
hsa-miR-139-5p	-1.492	0.056	HUH6_LIVER	LIHC
hsa-miR-139-5p	-1.476	0.064	JHH4_LIVER	LIHC

hsa-miR-139-5p	-1.469	0.065	HLF_LIVER	LIHC
hsa-miR-139-5p	-1.454	0.069	SNU761_LIVER	LIHC
hsa-miR-139-5p	-1.485	0.070	JHH7_LIVER	LIHC
hsa-miR-139-5p	-1.439	0.088	SNU182_LIVER	LIHC
hsa-miR-139-5p	-1.422	0.092	HUH1_LIVER	LIHC
hsa-miR-139-5p	-1.441	0.099	HUH7_LIVER	LIHC
hsa-miR-139-5p	-1.716	0.000	MORCPR_LUNG	LUAD
hsa-miR-139-5p	-1.712	0.002	NCIH2087_LUNG	LUAD
hsa-miR-139-5p	-1.617	0.002	PC14_LUNG	LUAD
hsa-miR-139-5p	-1.670	0.002	NCIH441_LUNG	LUAD
hsa-miR-139-5p	-1.747	0.002	NCIH2122_LUNG	LUAD
hsa-miR-139-5p	-1.685	0.002	NCIH2030_LUNG	LUAD
hsa-miR-139-5p	-1.686	0.003	RERFLCAD1_LUNG	LUAD
hsa-miR-139-5p	-1.726	0.003	NCIH3122_LUNG	LUAD
hsa-miR-139-5p	-1.631	0.003	NCIH23_LUNG	LUAD
hsa-miR-139-5p	-1.622	0.004	NCIH1437_LUNG	LUAD
hsa-miR-139-5p	-1.642	0.004	NCIH2126_LUNG	LUAD
hsa-miR-139-5p	-1.660	0.004	HCC515_LUNG	LUAD
hsa-miR-139-5p	-1.681	0.005	A427_LUNG	LUAD
hsa-miR-139-5p	-1.612	0.005	NCIH838_LUNG	LUAD
hsa-miR-139-5p	-1.650	0.006	HCC2429_LUNG	LUAD
hsa-miR-139-5p	-1.585	0.008	ABC1_LUNG	LUAD
hsa-miR-139-5p	-1.675	0.008	A549_LUNG	LUAD
hsa-miR-139-5p	-1.574	0.008	NCIH2023_LUNG	LUAD
hsa-miR-139-5p	-1.582	0.008	HCC461_LUNG	LUAD
hsa-miR-139-5p	-1.641	0.008	HOP62_LUNG	LUAD
hsa-miR-139-5p	-1.619	0.009	NCIH1648_LUNG	LUAD
hsa-miR-139-5p	-1.619	0.009	NCIH1568_LUNG	LUAD
hsa-miR-139-5p	-1.627	0.009	NCIH1944_LUNG	LUAD
hsa-miR-139-5p	-1.587	0.010	HCC2450_LUNG	LUAD
hsa-miR-139-5p	-1.789	0.011	NCIH1573_LUNG	LUAD
hsa-miR-139-5p	-1.599	0.014	NCIH1792_LUNG	LUAD
hsa-miR-139-5p	-1.585	0.015	NCIH1793_LUNG	LUAD
hsa-miR-139-5p	-1.567	0.015	LXF289_LUNG	LUAD
hsa-miR-139-5p	-1.578	0.023	NCIH1693_LUNG	LUAD
hsa-miR-139-5p	-1.563	0.026	EKVX_LUNG	LUAD
hsa-miR-139-5p	-1.518	0.030	HCC827_LUNG	LUAD
hsa-miR-139-5p	-1.546	0.032	HCC2935_LUNG	LUAD
hsa-miR-139-5p	-1.556	0.036	NCIH2291_LUNG	LUAD
hsa-miR-139-5p	-1.743	0.000	HCC15_LUNG	LUSC
hsa-miR-139-5p	-1.621	0.001	LK2_LUNG	LUSC
hsa-miR-139-5p	-1.632	0.001	NCIH1703_LUNG	LUSC
hsa-miR-139-5p	-1.598	0.001	RERFLCAI_LUNG	LUSC

hsa-miR-139-5p	-1.565	0.002	NCIH2170_LUNG	LUSC
hsa-miR-139-5p	-1.575	0.002	EPLC272H_LUNG	LUSC
hsa-miR-139-5p	-1.560	0.002	KNS62_LUNG	LUSC
hsa-miR-139-5p	-1.538	0.002	HARA_LUNG	LUSC
hsa-miR-139-5p	-1.571	0.003	NCIH520_LUNG	LUSC
hsa-miR-139-5p	-1.540	0.003	SKMES1_LUNG	LUSC
hsa-miR-139-5p	-1.518	0.003	LUDLU1_LUNG	LUSC
hsa-miR-139-5p	-1.529	0.004	CALU1_LUNG	LUSC
hsa-miR-139-5p	-1.524	0.005	HCC95_LUNG	LUSC
hsa-miR-139-5p	-1.796	0.000	SH10TC_STOMACH	STAD
hsa-miR-139-5p	-1.656	0.000	AGS_STOMACH	STAD
hsa-miR-139-5p	-1.682	0.000	KE39_STOMACH	STAD
hsa-miR-139-5p	-1.711	0.000	GSS_STOMACH	STAD
hsa-miR-139-5p	-1.741	0.000	MKN45_STOMACH	STAD
hsa-miR-139-5p	-1.631	0.001	MKN74_STOMACH	STAD
hsa-miR-139-5p	-1.642	0.001	SNU216_STOMACH	STAD
hsa-miR-139-5p	-1.618	0.001	GCIY_STOMACH	STAD
hsa-miR-139-5p	-1.650	0.001	SNU601_STOMACH	STAD
hsa-miR-139-5p	-1.633	0.001	SNU719_STOMACH	STAD
hsa-miR-139-5p	-1.692	0.001	GSU_STOMACH	STAD
hsa-miR-139-5p	-1.660	0.001	HS746T_STOMACH	STAD
hsa-miR-139-5p	-1.643	0.001	SNU1_STOMACH	STAD
hsa-miR-139-5p	-1.648	0.001	HGC27_STOMACH	STAD
hsa-miR-139-5p	-1.616	0.001	NUGC3_STOMACH	STAD
hsa-miR-139-5p	-1.618	0.002	NCIN87_STOMACH	STAD
hsa-miR-139-5p	-1.570	0.003	2313287_STOMACH	STAD
hsa-miR-139-5p	-1.621	0.003	LMSU_STOMACH	STAD
hsa-miR-139-5p	-1.483	0.007	FU97_STOMACH	STAD
hsa-miR-143-3p	-1.434	0.080	SCABER_URINARY_TRACT	BLCA
hsa-miR-143-3p	-1.955	0.000	MCF7_BREAST	BRCA
hsa-miR-143-3p	-1.864	0.000	HCC1954_BREAST	BRCA
hsa-miR-143-3p	-1.856	0.000	HCC1143_BREAST	BRCA
hsa-miR-143-3p	-1.831	0.000	DU4475_BREAST	BRCA
hsa-miR-143-3p	-1.823	0.000	KPL1_BREAST	BRCA
hsa-miR-143-3p	-1.805	0.000	CAL51_BREAST	BRCA
hsa-miR-143-3p	-1.791	0.000	MDAMB468_BREAST	BRCA
hsa-miR-143-3p	-1.790	0.000	SUM159PT_BREAST	BRCA
hsa-miR-143-3p	-1.789	0.000	HCC202_BREAST	BRCA
hsa-miR-143-3p	-1.780	0.000	MDAMB453_BREAST	BRCA
hsa-miR-143-3p	-1.771	0.000	HS578T_BREAST	BRCA
hsa-miR-143-3p	-1.770	0.000	MDAMB415_BREAST	BRCA
hsa-miR-143-3p	-1.770	0.000	HCC1428_BREAST	BRCA
hsa-miR-143-3p	-1.766	0.000	HCC1937_BREAST	BRCA

hsa-miR-143-3p	-1.746	0.000	HMC18_BREAST	BRCA
hsa-miR-143-3p	-1.727	0.000	EFM19_BREAST	BRCA
hsa-miR-143-3p	-1.725	0.000	ZR751_BREAST	BRCA
hsa-miR-143-3p	-1.788	0.000	AU565_BREAST	BRCA
hsa-miR-143-3p	-1.782	0.000	HCC1419_BREAST	BRCA
hsa-miR-143-3p	-1.759	0.000	MDAMB436_BREAST	BRCA
hsa-miR-143-3p	-1.727	0.001	SKBR3_BREAST	BRCA
hsa-miR-143-3p	-1.728	0.001	MDAMB231_BREAST	BRCA
hsa-miR-143-3p	-1.702	0.001	MDAMB157_BREAST	BRCA
hsa-miR-143-3p	-1.713	0.001	HCC1395_BREAST	BRCA
hsa-miR-143-3p	-1.720	0.001	HCC1806_BREAST	BRCA
hsa-miR-143-3p	-1.683	0.001	CAMA1_BREAST	BRCA
hsa-miR-143-3p	-1.697	0.003	BT549_BREAST	BRCA
hsa-miR-143-3p	-1.620	0.003	JIMT1_BREAST	BRCA
hsa-miR-143-3p	-1.663	0.001	KYSE410_OESOPHAGUS	ESCA
hsa-miR-143-3p	-1.684	0.001	TE1_OESOPHAGUS	ESCA
hsa-miR-143-3p	-1.685	0.001	TE5_OESOPHAGUS	ESCA
hsa-miR-143-3p	-1.628	0.001	KYSE30_OESOPHAGUS	ESCA
hsa-miR-143-3p	-1.687	0.001	KYSE150_OESOPHAGUS	ESCA
hsa-miR-143-3p	-1.684	0.002	KYSE270_OESOPHAGUS	ESCA
hsa-miR-143-3p	-1.614	0.002	OE21_OESOPHAGUS	ESCA
hsa-miR-143-3p	-1.641	0.002	OE33_OESOPHAGUS	ESCA
hsa-miR-143-3p	-1.565	0.003	KYSE450_OESOPHAGUS	ESCA
hsa-miR-143-3p	-1.602	0.004	TE10_OESOPHAGUS	ESCA
hsa-miR-143-3p	-1.611	0.004	KYSE140_OESOPHAGUS	ESCA
hsa-miR-143-3p	-1.572	0.005	OACM51_OESOPHAGUS	ESCA
hsa-miR-143-3p	-1.571	0.005	KYSE520_OESOPHAGUS	ESCA
hsa-miR-143-3p	-1.622	0.005	TE6_OESOPHAGUS	ESCA
hsa-miR-143-3p	-1.547	0.007	SKGT4_OESOPHAGUS	ESCA
hsa-miR-143-3p	-1.581	0.008	TE4_OESOPHAGUS	ESCA
hsa-miR-143-3p	-1.530	0.008	KYSE70_OESOPHAGUS	ESCA
hsa-miR-143-3p	-1.568	0.009	TE9_OESOPHAGUS	ESCA
hsa-miR-143-3p	-1.578	0.010	FLO1_OESOPHAGUS	ESCA
hsa-miR-143-3p	-1.517	0.013	KYSE180_OESOPHAGUS	ESCA
hsa-miR-143-3p	-1.506	0.014	TE11_OESOPHAGUS	ESCA
hsa-miR-143-3p	-1.543	0.015	ESO26_OESOPHAGUS	ESCA
hsa-miR-143-3p	-1.508	0.018	KYSE510_OESOPHAGUS	ESCA
hsa-miR-143-3p	-1.585	0.019	TE8_OESOPHAGUS	ESCA
hsa-miR-143-3p	-1.502	0.028	HCC2935_LUNG	LUAD
hsa-miR-143-3p	-1.448	0.041	NCIH3122_LUNG	LUAD
hsa-miR-143-3p	-1.398	0.044	NCIH2087_LUNG	LUAD
hsa-miR-143-3p	-1.423	0.045	HCC2429_LUNG	LUAD
hsa-miR-143-3p	-1.396	0.054	LXF289_LUNG	LUAD

hsa-miR-143-3p	-1.382	0.055	NCIH23_LUNG	LUAD
hsa-miR-143-3p	-1.395	0.057	NCIH2291_LUNG	LUAD
hsa-miR-143-3p	-1.386	0.057	EKVX_LUNG	LUAD
hsa-miR-143-3p	-1.380	0.059	NCIH441_LUNG	LUAD
hsa-miR-143-3p	-1.382	0.060	NCIH2126_LUNG	LUAD
hsa-miR-143-3p	-1.359	0.061	PC14_LUNG	LUAD
hsa-miR-143-3p	-1.383	0.064	HCC515_LUNG	LUAD
hsa-miR-143-3p	-1.360	0.067	NCIH1437_LUNG	LUAD
hsa-miR-143-3p	-1.368	0.067	HCC2450_LUNG	LUAD
hsa-miR-143-3p	-1.343	0.078	HCC461_LUNG	LUAD
hsa-miR-143-3p	-1.342	0.083	NCIH2030_LUNG	LUAD
hsa-miR-143-3p	-1.350	0.090	NCIH1648_LUNG	LUAD
hsa-miR-143-3p	-1.348	0.091	A549_LUNG	LUAD
hsa-miR-143-3p	-1.322	0.092	ABC1_LUNG	LUAD
hsa-miR-143-3p	-1.323	0.092	NCIH838_LUNG	LUAD
hsa-miR-143-3p	-1.323	0.095	NCIH2023_LUNG	LUAD
hsa-miR-143-3p	-1.828	0.000	HCC15_LUNG	LUSC
hsa-miR-143-3p	-1.692	0.001	LK2_LUNG	LUSC
hsa-miR-143-3p	-1.616	0.001	KNS62_LUNG	LUSC
hsa-miR-143-3p	-1.621	0.002	NCIH2170_LUNG	LUSC
hsa-miR-143-3p	-1.571	0.002	EPLC272H_LUNG	LUSC
hsa-miR-143-3p	-1.551	0.002	HARA_LUNG	LUSC
hsa-miR-143-3p	-1.558	0.002	RERFLCAI_LUNG	LUSC
hsa-miR-143-3p	-1.584	0.002	NCIH1703_LUNG	LUSC
hsa-miR-143-3p	-1.580	0.002	SKMES1_LUNG	LUSC
hsa-miR-143-3p	-1.543	0.003	LUDLU1_LUNG	LUSC
hsa-miR-143-3p	-1.584	0.003	HCC95_LUNG	LUSC
hsa-miR-143-3p	-1.539	0.003	NCIH520_LUNG	LUSC
hsa-miR-143-3p	-1.538	0.004	CALU1_LUNG	LUSC
hsa-miR-143-3p	-1.673	0.000	AGS_STOMACH	STAD
hsa-miR-143-3p	-1.620	0.000	KE39_STOMACH	STAD
hsa-miR-143-3p	-1.711	0.000	SH10TC_STOMACH	STAD
hsa-miR-143-3p	-1.659	0.000	GSU_STOMACH	STAD
hsa-miR-143-3p	-1.692	0.000	MKN45_STOMACH	STAD
hsa-miR-143-3p	-1.742	0.000	GSS_STOMACH	STAD
hsa-miR-143-3p	-1.615	0.001	GCIY_STOMACH	STAD
hsa-miR-143-3p	-1.663	0.001	SNU216_STOMACH	STAD
hsa-miR-143-3p	-1.698	0.001	HGC27_STOMACH	STAD
hsa-miR-143-3p	-1.635	0.001	SNU601_STOMACH	STAD
hsa-miR-143-3p	-1.675	0.001	NCIN87_STOMACH	STAD
hsa-miR-143-3p	-1.612	0.001	MKN74_STOMACH	STAD
hsa-miR-143-3p	-1.604	0.001	SNU719_STOMACH	STAD
hsa-miR-143-3p	-1.662	0.001	HS746T_STOMACH	STAD

hsa-miR-143-3p	-1.598	0.001	SNU1_STOMACH	STAD
hsa-miR-143-3p	-1.567	0.003	2313287_STOMACH	STAD
hsa-miR-143-3p	-1.558	0.003	NUGC3_STOMACH	STAD
hsa-miR-143-3p	-1.594	0.003	LMSU_STOMACH	STAD
hsa-miR-143-3p	-1.565	0.004	FU97_STOMACH	STAD
hsa-miR-143-5p	-1.465	0.021	KYSE270_OESOPHAGUS	ESCA
hsa-miR-143-5p	-1.388	0.035	TE1_OESOPHAGUS	ESCA
hsa-miR-143-5p	-1.406	0.036	KYSE410_OESOPHAGUS	ESCA
hsa-miR-143-5p	-1.391	0.047	TE5_OESOPHAGUS	ESCA
hsa-miR-143-5p	-1.376	0.050	KYSE70_OESOPHAGUS	ESCA
hsa-miR-143-5p	-1.385	0.050	OE33_OESOPHAGUS	ESCA
hsa-miR-143-5p	-1.414	0.053	TE8_OESOPHAGUS	ESCA
hsa-miR-143-5p	-1.385	0.074	FLO1_OESOPHAGUS	ESCA
hsa-miR-143-5p	-1.324	0.075	KYSE30_OESOPHAGUS	ESCA
hsa-miR-143-5p	-1.336	0.077	TE9_OESOPHAGUS	ESCA
hsa-miR-143-5p	-1.379	0.079	TE6_OESOPHAGUS	ESCA
hsa-miR-143-5p	-1.333	0.085	OACM51_OESOPHAGUS	ESCA
hsa-miR-143-5p	-1.308	0.096	OE21_OESOPHAGUS	ESCA
hsa-miR-143-5p	-1.336	0.099	KYSE180_OESOPHAGUS	ESCA
hsa-miR-143-5p	-1.671	0.001	GSS_STOMACH	STAD
hsa-miR-143-5p	-1.555	0.002	GCIY_STOMACH	STAD
hsa-miR-143-5p	-1.543	0.002	GSU_STOMACH	STAD
hsa-miR-143-5p	-1.540	0.002	MKN74_STOMACH	STAD
hsa-miR-143-5p	-1.569	0.003	SH10TC_STOMACH	STAD
hsa-miR-143-5p	-1.541	0.003	HS746T_STOMACH	STAD
hsa-miR-143-5p	-1.537	0.003	AGS_STOMACH	STAD
hsa-miR-143-5p	-1.536	0.004	MKN45_STOMACH	STAD
hsa-miR-143-5p	-1.520	0.004	SNU216_STOMACH	STAD
hsa-miR-143-5p	-1.462	0.005	NUGC3_STOMACH	STAD
hsa-miR-143-5p	-1.485	0.006	KE39_STOMACH	STAD
hsa-miR-143-5p	-1.491	0.006	LMSU_STOMACH	STAD
hsa-miR-143-5p	-1.477	0.006	SNU719_STOMACH	STAD
hsa-miR-143-5p	-1.502	0.006	HGC27_STOMACH	STAD
hsa-miR-143-5p	-1.468	0.006	SNU1_STOMACH	STAD
hsa-miR-143-5p	-1.440	0.009	2313287_STOMACH	STAD
hsa-miR-143-5p	-1.434	0.012	SNU601_STOMACH	STAD
hsa-miR-143-5p	-1.450	0.013	NCIN87_STOMACH	STAD
hsa-miR-143-5p	-1.349	0.030	FU97_STOMACH	STAD
hsa-miR-145-3p	-1.648	0.003	RT112_URINARY_TRACT	BLCA
hsa-miR-145-3p	-1.648	0.003	RT112_URINARY_TRACT	BLCA
hsa-miR-145-3p	-1.597	0.007	SCABER_URINARY_TRACT	BLCA
hsa-miR-145-3p	-1.597	0.007	SCABER_URINARY_TRACT	BLCA
hsa-miR-145-3p	-1.587	0.009	UMUC13_URINARY_TRACT	BLCA

hsa-miR-145-3p	-1.587	0.009	UMUC13_URINARY_TRACT	BLCA
hsa-miR-145-3p	-1.560	0.014	RT11284_URINARY_TRACT	BLCA
hsa-miR-145-3p	-1.560	0.014	RT11284_URINARY_TRACT	BLCA
hsa-miR-145-3p	-1.528	0.014	639V_URINARY_TRACT	BLCA
hsa-miR-145-3p	-1.528	0.014	639V_URINARY_TRACT	BLCA
hsa-miR-145-3p	-1.522	0.019	UMUC14_URINARY_TRACT	BLCA
hsa-miR-145-3p	-1.522	0.019	UMUC14_URINARY_TRACT	BLCA
hsa-miR-145-3p	-1.531	0.023	253J_URINARY_TRACT	BLCA
hsa-miR-145-3p	-1.531	0.023	253J_URINARY_TRACT	BLCA
hsa-miR-145-3p	-1.503	0.026	5637_URINARY_TRACT	BLCA
hsa-miR-145-3p	-1.503	0.026	5637_URINARY_TRACT	BLCA
hsa-miR-145-3p	-1.505	0.030	UMUC11_URINARY_TRACT	BLCA
hsa-miR-145-3p	-1.505	0.030	UMUC11_URINARY_TRACT	BLCA
hsa-miR-145-3p	-1.559	0.031	HT1376_URINARY_TRACT	BLCA
hsa-miR-145-3p	-1.559	0.031	HT1376_URINARY_TRACT	BLCA
hsa-miR-145-3p	-1.466	0.034	UMUC1_URINARY_TRACT	BLCA
hsa-miR-145-3p	-1.466	0.034	UMUC1_URINARY_TRACT	BLCA
hsa-miR-145-3p	-1.460	0.035	KU1919_URINARY_TRACT	BLCA
hsa-miR-145-3p	-1.460	0.035	KU1919_URINARY_TRACT	BLCA
hsa-miR-145-3p	-1.498	0.036	TCCSUP_URINARY_TRACT	BLCA
hsa-miR-145-3p	-1.498	0.036	TCCSUP_URINARY_TRACT	BLCA
hsa-miR-145-3p	-1.493	0.037	BFTC905_URINARY_TRACT	BLCA
hsa-miR-145-3p	-1.493	0.037	BFTC905_URINARY_TRACT	BLCA
hsa-miR-145-3p	-1.459	0.039	CAL29_URINARY_TRACT	BLCA
hsa-miR-145-3p	-1.459	0.039	CAL29_URINARY_TRACT	BLCA
hsa-miR-145-3p	-1.465	0.040	KMBC2_URINARY_TRACT	BLCA
hsa-miR-145-3p	-1.465	0.040	KMBC2_URINARY_TRACT	BLCA
hsa-miR-145-3p	-1.485	0.048	T24_URINARY_TRACT	BLCA
hsa-miR-145-3p	-1.485	0.048	T24_URINARY_TRACT	BLCA
hsa-miR-145-3p	-1.463	0.057	VMCUB1_URINARY_TRACT	BLCA
hsa-miR-145-3p	-1.463	0.057	VMCUB1_URINARY_TRACT	BLCA
hsa-miR-145-3p	-1.451	0.069	647V_URINARY_TRACT	BLCA
hsa-miR-145-3p	-1.451	0.069	647V_URINARY_TRACT	BLCA
hsa-miR-145-3p	-1.434	0.072	UMUC3_URINARY_TRACT	BLCA
hsa-miR-145-3p	-1.434	0.072	UMUC3_URINARY_TRACT	BLCA
hsa-miR-145-3p	-1.412	0.080	JMSU1_URINARY_TRACT	BLCA
hsa-miR-145-3p	-1.412	0.080	JMSU1_URINARY_TRACT	BLCA
hsa-miR-145-3p	-1.801	0.000	HCC1954_BREAST	BRCA
hsa-miR-145-3p	-1.740	0.000	KPL1_BREAST	BRCA
hsa-miR-145-3p	-1.801	0.000	HCC1954_BREAST	BRCA
hsa-miR-145-3p	-1.740	0.000	KPL1_BREAST	BRCA
hsa-miR-145-3p	-1.693	0.000	EFM19_BREAST	BRCA
hsa-miR-145-3p	-1.693	0.000	EFM19_BREAST	BRCA

hsa-miR-145-3p	-1.747	0.000	AU565_BREAST	BRCA
hsa-miR-145-3p	-1.747	0.000	AU565_BREAST	BRCA
hsa-miR-145-3p	-1.740	0.000	SUM159PT_BREAST	BRCA
hsa-miR-145-3p	-1.740	0.000	SUM159PT_BREAST	BRCA
hsa-miR-145-3p	-1.698	0.000	CAL51_BREAST	BRCA
hsa-miR-145-3p	-1.698	0.000	CAL51_BREAST	BRCA
hsa-miR-145-3p	-1.705	0.000	HCC1395_BREAST	BRCA
hsa-miR-145-3p	-1.705	0.000	HCC1395_BREAST	BRCA
hsa-miR-145-3p	-1.710	0.001	HCC1143_BREAST	BRCA
hsa-miR-145-3p	-1.710	0.001	HCC1143_BREAST	BRCA
hsa-miR-145-3p	-1.690	0.001	HS578T_BREAST	BRCA
hsa-miR-145-3p	-1.690	0.001	HS578T_BREAST	BRCA
hsa-miR-145-3p	-1.664	0.001	MDAMB468_BREAST	BRCA
hsa-miR-145-3p	-1.664	0.001	MDAMB468_BREAST	BRCA
hsa-miR-145-3p	-1.653	0.001	ZR751_BREAST	BRCA
hsa-miR-145-3p	-1.653	0.001	ZR751_BREAST	BRCA
hsa-miR-145-3p	-1.730	0.001	MCF7_BREAST	BRCA
hsa-miR-145-3p	-1.730	0.001	MCF7_BREAST	BRCA
hsa-miR-145-3p	-1.706	0.001	HCC1428_BREAST	BRCA
hsa-miR-145-3p	-1.706	0.001	HCC1428_BREAST	BRCA
hsa-miR-145-3p	-1.762	0.001	MDAMB436_BREAST	BRCA
hsa-miR-145-3p	-1.762	0.001	MDAMB436_BREAST	BRCA
hsa-miR-145-3p	-1.673	0.001	HCC1937_BREAST	BRCA
hsa-miR-145-3p	-1.673	0.001	HCC1937_BREAST	BRCA
hsa-miR-145-3p	-1.681	0.001	HMC18_BREAST	BRCA
hsa-miR-145-3p	-1.681	0.001	HMC18_BREAST	BRCA
hsa-miR-145-3p	-1.698	0.001	CAMA1_BREAST	BRCA
hsa-miR-145-3p	-1.698	0.001	CAMA1_BREAST	BRCA
hsa-miR-145-3p	-1.648	0.001	MDAMB231_BREAST	BRCA
hsa-miR-145-3p	-1.648	0.001	MDAMB231_BREAST	BRCA
hsa-miR-145-3p	-1.663	0.002	HCC1419_BREAST	BRCA
hsa-miR-145-3p	-1.663	0.002	HCC1419_BREAST	BRCA
hsa-miR-145-3p	-1.649	0.002	MDAMB453_BREAST	BRCA
hsa-miR-145-3p	-1.649	0.002	MDAMB453_BREAST	BRCA
hsa-miR-145-3p	-1.668	0.002	HCC202_BREAST	BRCA
hsa-miR-145-3p	-1.668	0.002	HCC202_BREAST	BRCA
hsa-miR-145-3p	-1.639	0.003	MDAMB157_BREAST	BRCA
hsa-miR-145-3p	-1.639	0.003	MDAMB157_BREAST	BRCA
hsa-miR-145-3p	-1.601	0.003	SKBR3_BREAST	BRCA
hsa-miR-145-3p	-1.601	0.003	SKBR3_BREAST	BRCA
hsa-miR-145-3p	-1.654	0.003	MDAMB415_BREAST	BRCA
hsa-miR-145-3p	-1.654	0.003	MDAMB415_BREAST	BRCA
hsa-miR-145-3p	-1.686	0.004	DU4475_BREAST	BRCA

hsa-miR-145-3p	-1.686	0.004	DU4475_BREAST	BRCA
hsa-miR-145-3p	-1.631	0.004	BT549_BREAST	BRCA
hsa-miR-145-3p	-1.631	0.004	BT549_BREAST	BRCA
hsa-miR-145-3p	-1.589	0.004	JIMT1_BREAST	BRCA
hsa-miR-145-3p	-1.589	0.004	JIMT1_BREAST	BRCA
hsa-miR-145-3p	-1.555	0.007	HCC1806_BREAST	BRCA
hsa-miR-145-3p	-1.555	0.007	HCC1806_BREAST	BRCA
hsa-miR-145-3p	-1.712	0.001	KYSE150_OESOPHAGUS	ESCA
hsa-miR-145-3p	-1.712	0.001	KYSE150_OESOPHAGUS	ESCA
hsa-miR-145-3p	-1.651	0.001	KYSE30_OESOPHAGUS	ESCA
hsa-miR-145-3p	-1.651	0.001	KYSE30_OESOPHAGUS	ESCA
hsa-miR-145-3p	-1.589	0.002	KYSE450_OESOPHAGUS	ESCA
hsa-miR-145-3p	-1.589	0.002	KYSE450_OESOPHAGUS	ESCA
hsa-miR-145-3p	-1.629	0.003	KYSE520_OESOPHAGUS	ESCA
hsa-miR-145-3p	-1.629	0.003	KYSE520_OESOPHAGUS	ESCA
hsa-miR-145-3p	-1.635	0.004	FLO1_OESOPHAGUS	ESCA
hsa-miR-145-3p	-1.635	0.004	FLO1_OESOPHAGUS	ESCA
hsa-miR-145-3p	-1.653	0.004	KYSE140_OESOPHAGUS	ESCA
hsa-miR-145-3p	-1.653	0.004	KYSE140_OESOPHAGUS	ESCA
hsa-miR-145-3p	-1.575	0.004	KYSE70_OESOPHAGUS	ESCA
hsa-miR-145-3p	-1.575	0.004	KYSE70_OESOPHAGUS	ESCA
hsa-miR-145-3p	-1.582	0.004	TE5_OESOPHAGUS	ESCA
hsa-miR-145-3p	-1.582	0.004	TE5_OESOPHAGUS	ESCA
hsa-miR-145-3p	-1.607	0.004	KYSE270_OESOPHAGUS	ESCA
hsa-miR-145-3p	-1.607	0.004	KYSE270_OESOPHAGUS	ESCA
hsa-miR-145-3p	-1.564	0.004	TE1_OESOPHAGUS	ESCA
hsa-miR-145-3p	-1.564	0.004	TE1_OESOPHAGUS	ESCA
hsa-miR-145-3p	-1.559	0.005	OE21_OESOPHAGUS	ESCA
hsa-miR-145-3p	-1.559	0.005	OE21_OESOPHAGUS	ESCA
hsa-miR-145-3p	-1.550	0.005	KYSE410_OESOPHAGUS	ESCA
hsa-miR-145-3p	-1.550	0.005	KYSE410_OESOPHAGUS	ESCA
hsa-miR-145-3p	-1.573	0.005	TE11_OESOPHAGUS	ESCA
hsa-miR-145-3p	-1.573	0.005	TE11_OESOPHAGUS	ESCA
hsa-miR-145-3p	-1.630	0.006	TE6_OESOPHAGUS	ESCA
hsa-miR-145-3p	-1.630	0.006	TE6_OESOPHAGUS	ESCA
hsa-miR-145-3p	-1.557	0.007	SKGT4_OESOPHAGUS	ESCA
hsa-miR-145-3p	-1.557	0.007	SKGT4_OESOPHAGUS	ESCA
hsa-miR-145-3p	-1.511	0.016	TE8_OESOPHAGUS	ESCA
hsa-miR-145-3p	-1.511	0.016	TE8_OESOPHAGUS	ESCA
hsa-miR-145-3p	-1.508	0.016	KYSE510_OESOPHAGUS	ESCA
hsa-miR-145-3p	-1.508	0.016	KYSE510_OESOPHAGUS	ESCA
hsa-miR-145-3p	-1.471	0.018	TE9_OESOPHAGUS	ESCA
hsa-miR-145-3p	-1.471	0.018	TE9_OESOPHAGUS	ESCA

hsa-miR-145-3p	-1.489	0.019	TE10_OESOPHAGUS	ESCA
hsa-miR-145-3p	-1.489	0.019	TE10_OESOPHAGUS	ESCA
hsa-miR-145-3p	-1.454	0.022	OE33_OESOPHAGUS	ESCA
hsa-miR-145-3p	-1.454	0.022	OE33_OESOPHAGUS	ESCA
hsa-miR-145-3p	-1.483	0.022	OACM51_OESOPHAGUS	ESCA
hsa-miR-145-3p	-1.483	0.022	OACM51_OESOPHAGUS	ESCA
hsa-miR-145-3p	-1.449	0.023	KYSE180_OESOPHAGUS	ESCA
hsa-miR-145-3p	-1.449	0.023	KYSE180_OESOPHAGUS	ESCA
hsa-miR-145-3p	-1.467	0.024	ESO26_OESOPHAGUS	ESCA
hsa-miR-145-3p	-1.467	0.024	ESO26_OESOPHAGUS	ESCA
hsa-miR-145-3p	-1.494	0.024	TE4_OESOPHAGUS	ESCA
hsa-miR-145-3p	-1.494	0.024	TE4_OESOPHAGUS	ESCA
hsa-miR-145-3p	-1.641	0.004	NCIH2122_LUNG	LUAD
hsa-miR-145-3p	-1.641	0.004	NCIH2122_LUNG	LUAD
hsa-miR-145-3p	-1.637	0.005	NCIH2087_LUNG	LUAD
hsa-miR-145-3p	-1.637	0.005	NCIH2087_LUNG	LUAD
hsa-miR-145-3p	-1.581	0.008	NCIH23_LUNG	LUAD
hsa-miR-145-3p	-1.581	0.008	NCIH23_LUNG	LUAD
hsa-miR-145-3p	-1.512	0.010	NCIH2126_LUNG	LUAD
hsa-miR-145-3p	-1.512	0.010	NCIH2126_LUNG	LUAD
hsa-miR-145-3p	-1.577	0.011	NCIH441_LUNG	LUAD
hsa-miR-145-3p	-1.577	0.011	NCIH441_LUNG	LUAD
hsa-miR-145-3p	-1.565	0.014	RERFLCAD1_LUNG	LUAD
hsa-miR-145-3p	-1.565	0.014	RERFLCAD1_LUNG	LUAD
hsa-miR-145-3p	-1.504	0.014	NCIH838_LUNG	LUAD
hsa-miR-145-3p	-1.504	0.014	NCIH838_LUNG	LUAD
hsa-miR-145-3p	-1.481	0.014	PC14_LUNG	LUAD
hsa-miR-145-3p	-1.481	0.014	PC14_LUNG	LUAD
hsa-miR-145-3p	-1.551	0.016	NCIH1568_LUNG	LUAD
hsa-miR-145-3p	-1.551	0.016	NCIH1568_LUNG	LUAD
hsa-miR-145-3p	-1.553	0.016	A549_LUNG	LUAD
hsa-miR-145-3p	-1.553	0.016	A549_LUNG	LUAD
hsa-miR-145-3p	-1.560	0.016	NCIH1648_LUNG	LUAD
hsa-miR-145-3p	-1.560	0.016	NCIH1648_LUNG	LUAD
hsa-miR-145-3p	-1.558	0.020	A427_LUNG	LUAD
hsa-miR-145-3p	-1.558	0.020	A427_LUNG	LUAD
hsa-miR-145-3p	-1.471	0.021	HCC461_LUNG	LUAD
hsa-miR-145-3p	-1.471	0.021	HCC461_LUNG	LUAD
hsa-miR-145-3p	-1.502	0.022	MORCPR_LUNG	LUAD
hsa-miR-145-3p	-1.502	0.022	MORCPR_LUNG	LUAD
hsa-miR-145-3p	-1.518	0.024	HCC2935_LUNG	LUAD
hsa-miR-145-3p	-1.518	0.024	HCC2935_LUNG	LUAD
hsa-miR-145-3p	-1.456	0.025	NCIH2023_LUNG	LUAD

hsa-miR-145-3p	-1.456	0.025	NCIH2023_LUNG	LUAD
hsa-miR-145-3p	-1.445	0.027	NCIH1437_LUNG	LUAD
hsa-miR-145-3p	-1.445	0.027	NCIH1437_LUNG	LUAD
hsa-miR-145-3p	-1.491	0.027	LXF289_LUNG	LUAD
hsa-miR-145-3p	-1.491	0.027	LXF289_LUNG	LUAD
hsa-miR-145-3p	-1.528	0.027	NCIH2030_LUNG	LUAD
hsa-miR-145-3p	-1.528	0.027	NCIH2030_LUNG	LUAD
hsa-miR-145-3p	-1.460	0.028	HCC2450_LUNG	LUAD
hsa-miR-145-3p	-1.460	0.028	HCC2450_LUNG	LUAD
hsa-miR-145-3p	-1.483	0.029	NCIH1944_LUNG	LUAD
hsa-miR-145-3p	-1.483	0.029	NCIH1944_LUNG	LUAD
hsa-miR-145-3p	-1.517	0.031	HOP62_LUNG	LUAD
hsa-miR-145-3p	-1.517	0.031	HOP62_LUNG	LUAD
hsa-miR-145-3p	-1.527	0.032	NCIH2291_LUNG	LUAD
hsa-miR-145-3p	-1.527	0.032	NCIH2291_LUNG	LUAD
hsa-miR-145-3p	-1.449	0.035	ABC1_LUNG	LUAD
hsa-miR-145-3p	-1.449	0.035	ABC1_LUNG	LUAD
hsa-miR-145-3p	-1.480	0.035	EKVX_LUNG	LUAD
hsa-miR-145-3p	-1.480	0.035	EKVX_LUNG	LUAD
hsa-miR-145-3p	-1.456	0.036	HCC2429_LUNG	LUAD
hsa-miR-145-3p	-1.456	0.036	HCC2429_LUNG	LUAD
hsa-miR-145-3p	-1.452	0.037	NCIH1793_LUNG	LUAD
hsa-miR-145-3p	-1.452	0.037	NCIH1793_LUNG	LUAD
hsa-miR-145-3p	-1.444	0.038	HCC827_LUNG	LUAD
hsa-miR-145-3p	-1.444	0.038	HCC827_LUNG	LUAD
hsa-miR-145-3p	-1.471	0.039	HCC515_LUNG	LUAD
hsa-miR-145-3p	-1.471	0.039	HCC515_LUNG	LUAD
hsa-miR-145-3p	-1.450	0.039	NCIH1792_LUNG	LUAD
hsa-miR-145-3p	-1.450	0.039	NCIH1792_LUNG	LUAD
hsa-miR-145-3p	-1.462	0.043	NCIH3122_LUNG	LUAD
hsa-miR-145-3p	-1.462	0.043	NCIH3122_LUNG	LUAD
hsa-miR-145-3p	-1.467	0.046	NCIH1693_LUNG	LUAD
hsa-miR-145-3p	-1.467	0.046	NCIH1693_LUNG	LUAD
hsa-miR-145-3p	-1.403	0.099	NCIH1573_LUNG	LUAD
hsa-miR-145-3p	-1.403	0.099	NCIH1573_LUNG	LUAD
hsa-miR-145-3p	-1.910	0.000	HCC15_LUNG	LUSC
hsa-miR-145-3p	-1.752	0.000	LK2_LUNG	LUSC
hsa-miR-145-3p	-1.716	0.000	SKMES1_LUNG	LUSC
hsa-miR-145-3p	-1.910	0.000	HCC15_LUNG	LUSC
hsa-miR-145-3p	-1.752	0.000	LK2_LUNG	LUSC
hsa-miR-145-3p	-1.716	0.000	SKMES1_LUNG	LUSC
hsa-miR-145-3p	-1.654	0.000	RERFLCAI_LUNG	LUSC
hsa-miR-145-3p	-1.654	0.000	RERFLCAI_LUNG	LUSC

hsa-miR-145-3p	-1.713	0.000	NCIH1703_LUNG	LUSC
hsa-miR-145-3p	-1.713	0.000	NCIH1703_LUNG	LUSC
hsa-miR-145-3p	-1.669	0.000	EPLC272H_LUNG	LUSC
hsa-miR-145-3p	-1.669	0.000	EPLC272H_LUNG	LUSC
hsa-miR-145-3p	-1.686	0.000	NCIH520_LUNG	LUSC
hsa-miR-145-3p	-1.686	0.000	NCIH520_LUNG	LUSC
hsa-miR-145-3p	-1.734	0.000	HARA_LUNG	LUSC
hsa-miR-145-3p	-1.734	0.000	HARA_LUNG	LUSC
hsa-miR-145-3p	-1.683	0.001	KNS62_LUNG	LUSC
hsa-miR-145-3p	-1.683	0.001	KNS62_LUNG	LUSC
hsa-miR-145-3p	-1.683	0.001	HCC95_LUNG	LUSC
hsa-miR-145-3p	-1.683	0.001	HCC95_LUNG	LUSC
hsa-miR-145-3p	-1.650	0.001	LUDLU1_LUNG	LUSC
hsa-miR-145-3p	-1.650	0.001	LUDLU1_LUNG	LUSC
hsa-miR-145-3p	-1.669	0.001	CALU1_LUNG	LUSC
hsa-miR-145-3p	-1.669	0.001	CALU1_LUNG	LUSC
hsa-miR-145-3p	-1.658	0.001	NCIH2170_LUNG	LUSC
hsa-miR-145-3p	-1.658	0.001	NCIH2170_LUNG	LUSC
hsa-miR-145-3p	-1.662	0.000	MKN74_STOMACH	STAD
hsa-miR-145-3p	-1.662	0.000	MKN74_STOMACH	STAD
hsa-miR-145-3p	-1.621	0.001	GSU_STOMACH	STAD
hsa-miR-145-3p	-1.621	0.001	GSU_STOMACH	STAD
hsa-miR-145-3p	-1.660	0.001	MKN45_STOMACH	STAD
hsa-miR-145-3p	-1.660	0.001	MKN45_STOMACH	STAD
hsa-miR-145-3p	-1.630	0.001	SNU719_STOMACH	STAD
hsa-miR-145-3p	-1.630	0.001	SNU719_STOMACH	STAD
hsa-miR-145-3p	-1.666	0.001	GSS_STOMACH	STAD
hsa-miR-145-3p	-1.666	0.001	GSS_STOMACH	STAD
hsa-miR-145-3p	-1.596	0.001	HS746T_STOMACH	STAD
hsa-miR-145-3p	-1.596	0.001	HS746T_STOMACH	STAD
hsa-miR-145-3p	-1.590	0.001	SNU216_STOMACH	STAD
hsa-miR-145-3p	-1.590	0.001	SNU216_STOMACH	STAD
hsa-miR-145-3p	-1.608	0.002	SH10TC_STOMACH	STAD
hsa-miR-145-3p	-1.608	0.002	SH10TC_STOMACH	STAD
hsa-miR-145-3p	-1.546	0.002	GCIY_STOMACH	STAD
hsa-miR-145-3p	-1.546	0.002	GCIY_STOMACH	STAD
hsa-miR-145-3p	-1.569	0.002	SNU601_STOMACH	STAD
hsa-miR-145-3p	-1.569	0.002	SNU601_STOMACH	STAD
hsa-miR-145-3p	-1.546	0.002	KE39_STOMACH	STAD
hsa-miR-145-3p	-1.546	0.002	KE39_STOMACH	STAD
hsa-miR-145-3p	-1.588	0.002	LMSU_STOMACH	STAD
hsa-miR-145-3p	-1.588	0.002	LMSU_STOMACH	STAD
hsa-miR-145-3p	-1.590	0.002	HGC27_STOMACH	STAD

hsa-miR-145-3p	-1.590	0.002	HGC27_STOMACH	STAD
hsa-miR-145-3p	-1.600	0.003	NCIN87_STOMACH	STAD
hsa-miR-145-3p	-1.600	0.003	NCIN87_STOMACH	STAD
hsa-miR-145-3p	-1.546	0.003	AGS_STOMACH	STAD
hsa-miR-145-3p	-1.546	0.003	AGS_STOMACH	STAD
hsa-miR-145-3p	-1.496	0.004	2313287_STOMACH	STAD
hsa-miR-145-3p	-1.496	0.004	2313287_STOMACH	STAD
hsa-miR-145-3p	-1.501	0.004	SNU1_STOMACH	STAD
hsa-miR-145-3p	-1.501	0.004	SNU1_STOMACH	STAD
hsa-miR-145-3p	-1.501	0.005	NUGC3_STOMACH	STAD
hsa-miR-145-3p	-1.501	0.005	NUGC3_STOMACH	STAD
hsa-miR-145-3p	-1.479	0.007	FU97_STOMACH	STAD
hsa-miR-145-3p	-1.479	0.007	FU97_STOMACH	STAD
hsa-miR-145-5p	-1.376	0.099	639V_URINARY_TRACT	BLCA
hsa-miR-145-5p	-1.376	0.099	639V_URINARY_TRACT	BLCA
hsa-miR-145-5p	-1.753	0.000	AU565_BREAST	BRCA
hsa-miR-145-5p	-1.753	0.000	AU565_BREAST	BRCA
hsa-miR-145-5p	-1.742	0.000	MCF7_BREAST	BRCA
hsa-miR-145-5p	-1.742	0.000	MCF7_BREAST	BRCA
hsa-miR-145-5p	-1.731	0.000	HCC1954_BREAST	BRCA
hsa-miR-145-5p	-1.731	0.000	HCC1954_BREAST	BRCA
hsa-miR-145-5p	-1.722	0.000	HCC202_BREAST	BRCA
hsa-miR-145-5p	-1.722	0.000	HCC202_BREAST	BRCA
hsa-miR-145-5p	-1.638	0.001	KPL1_BREAST	BRCA
hsa-miR-145-5p	-1.638	0.001	KPL1_BREAST	BRCA
hsa-miR-145-5p	-1.648	0.002	SUM159PT_BREAST	BRCA
hsa-miR-145-5p	-1.648	0.002	SUM159PT_BREAST	BRCA
hsa-miR-145-5p	-1.642	0.002	HCC1143_BREAST	BRCA
hsa-miR-145-5p	-1.642	0.002	HCC1143_BREAST	BRCA
hsa-miR-145-5p	-1.652	0.002	MDAMB231_BREAST	BRCA
hsa-miR-145-5p	-1.652	0.002	MDAMB231_BREAST	BRCA
hsa-miR-145-5p	-1.632	0.002	CAL51_BREAST	BRCA
hsa-miR-145-5p	-1.632	0.002	CAL51_BREAST	BRCA
hsa-miR-145-5p	-1.655	0.002	EFM19_BREAST	BRCA
hsa-miR-145-5p	-1.655	0.002	EFM19_BREAST	BRCA
hsa-miR-145-5p	-1.665	0.002	MDAMB453_BREAST	BRCA
hsa-miR-145-5p	-1.665	0.002	MDAMB453_BREAST	BRCA
hsa-miR-145-5p	-1.608	0.002	HS578T_BREAST	BRCA
hsa-miR-145-5p	-1.608	0.002	HS578T_BREAST	BRCA
hsa-miR-145-5p	-1.648	0.002	BT549_BREAST	BRCA
hsa-miR-145-5p	-1.648	0.002	BT549_BREAST	BRCA
hsa-miR-145-5p	-1.586	0.003	HMC18_BREAST	BRCA
hsa-miR-145-5p	-1.586	0.003	HMC18_BREAST	BRCA

hsa-miR-145-5p	-1.605	0.003	HCC1937_BREAST	BRCA
hsa-miR-145-5p	-1.605	0.003	HCC1937_BREAST	BRCA
hsa-miR-145-5p	-1.588	0.003	MDAMB157_BREAST	BRCA
hsa-miR-145-5p	-1.588	0.003	MDAMB157_BREAST	BRCA
hsa-miR-145-5p	-1.622	0.003	HCC1419_BREAST	BRCA
hsa-miR-145-5p	-1.622	0.003	HCC1419_BREAST	BRCA
hsa-miR-145-5p	-1.616	0.003	MDAMB436_BREAST	BRCA
hsa-miR-145-5p	-1.616	0.003	MDAMB436_BREAST	BRCA
hsa-miR-145-5p	-1.580	0.004	SKBR3_BREAST	BRCA
hsa-miR-145-5p	-1.580	0.004	SKBR3_BREAST	BRCA
hsa-miR-145-5p	-1.670	0.004	DU4475_BREAST	BRCA
hsa-miR-145-5p	-1.670	0.004	DU4475_BREAST	BRCA
hsa-miR-145-5p	-1.580	0.005	CAMA1_BREAST	BRCA
hsa-miR-145-5p	-1.580	0.005	CAMA1_BREAST	BRCA
hsa-miR-145-5p	-1.565	0.005	HCC1395_BREAST	BRCA
hsa-miR-145-5p	-1.565	0.005	HCC1395_BREAST	BRCA
hsa-miR-145-5p	-1.581	0.006	MDAMB468_BREAST	BRCA
hsa-miR-145-5p	-1.581	0.006	MDAMB468_BREAST	BRCA
hsa-miR-145-5p	-1.566	0.007	MDAMB415_BREAST	BRCA
hsa-miR-145-5p	-1.566	0.007	MDAMB415_BREAST	BRCA
hsa-miR-145-5p	-1.543	0.009	JIMT1_BREAST	BRCA
hsa-miR-145-5p	-1.543	0.009	JIMT1_BREAST	BRCA
hsa-miR-145-5p	-1.542	0.010	HCC1428_BREAST	BRCA
hsa-miR-145-5p	-1.542	0.010	HCC1428_BREAST	BRCA
hsa-miR-145-5p	-1.500	0.014	ZR751_BREAST	BRCA
hsa-miR-145-5p	-1.500	0.014	ZR751_BREAST	BRCA
hsa-miR-145-5p	-1.487	0.020	HCC1806_BREAST	BRCA
hsa-miR-145-5p	-1.487	0.020	HCC1806_BREAST	BRCA
hsa-miR-145-5p	-1.593	0.003	OE21_OESOPHAGUS	ESCA
hsa-miR-145-5p	-1.593	0.003	OE21_OESOPHAGUS	ESCA
hsa-miR-145-5p	-1.566	0.006	KYSE270_OESOPHAGUS	ESCA
hsa-miR-145-5p	-1.566	0.006	KYSE270_OESOPHAGUS	ESCA
hsa-miR-145-5p	-1.505	0.008	KYSE410_OESOPHAGUS	ESCA
hsa-miR-145-5p	-1.505	0.008	KYSE410_OESOPHAGUS	ESCA
hsa-miR-145-5p	-1.515	0.009	KYSE140_OESOPHAGUS	ESCA
hsa-miR-145-5p	-1.515	0.009	KYSE140_OESOPHAGUS	ESCA
hsa-miR-145-5p	-1.503	0.009	KYSE30_OESOPHAGUS	ESCA
hsa-miR-145-5p	-1.503	0.009	KYSE30_OESOPHAGUS	ESCA
hsa-miR-145-5p	-1.476	0.012	TE1_OESOPHAGUS	ESCA
hsa-miR-145-5p	-1.476	0.012	TE1_OESOPHAGUS	ESCA
hsa-miR-145-5p	-1.518	0.012	TE5_OESOPHAGUS	ESCA
hsa-miR-145-5p	-1.518	0.012	TE5_OESOPHAGUS	ESCA
hsa-miR-145-5p	-1.484	0.014	OE33_OESOPHAGUS	ESCA

hsa-miR-145-5p	-1.484	0.014	OE33_OESOPHAGUS	ESCA
hsa-miR-145-5p	-1.524	0.018	FLO1_OESOPHAGUS	ESCA
hsa-miR-145-5p	-1.524	0.018	FLO1_OESOPHAGUS	ESCA
hsa-miR-145-5p	-1.486	0.018	KYSE520_OESOPHAGUS	ESCA
hsa-miR-145-5p	-1.486	0.018	KYSE520_OESOPHAGUS	ESCA
hsa-miR-145-5p	-1.465	0.022	KYSE180_OESOPHAGUS	ESCA
hsa-miR-145-5p	-1.465	0.022	KYSE180_OESOPHAGUS	ESCA
hsa-miR-145-5p	-1.457	0.029	TE8_OESOPHAGUS	ESCA
hsa-miR-145-5p	-1.457	0.029	TE8_OESOPHAGUS	ESCA
hsa-miR-145-5p	-1.423	0.034	TE10_OESOPHAGUS	ESCA
hsa-miR-145-5p	-1.423	0.034	TE10_OESOPHAGUS	ESCA
hsa-miR-145-5p	-1.421	0.037	ESO26_OESOPHAGUS	ESCA
hsa-miR-145-5p	-1.421	0.037	ESO26_OESOPHAGUS	ESCA
hsa-miR-145-5p	-1.412	0.039	KYSE450_OESOPHAGUS	ESCA
hsa-miR-145-5p	-1.412	0.039	KYSE450_OESOPHAGUS	ESCA
hsa-miR-145-5p	-1.408	0.039	OACM51_OESOPHAGUS	ESCA
hsa-miR-145-5p	-1.408	0.039	OACM51_OESOPHAGUS	ESCA
hsa-miR-145-5p	-1.438	0.042	SKGT4_OESOPHAGUS	ESCA
hsa-miR-145-5p	-1.438	0.042	SKGT4_OESOPHAGUS	ESCA
hsa-miR-145-5p	-1.444	0.046	KYSE150_OESOPHAGUS	ESCA
hsa-miR-145-5p	-1.444	0.046	KYSE150_OESOPHAGUS	ESCA
hsa-miR-145-5p	-1.364	0.052	KYSE70_OESOPHAGUS	ESCA
hsa-miR-145-5p	-1.364	0.052	KYSE70_OESOPHAGUS	ESCA
hsa-miR-145-5p	-1.366	0.060	TE9_OESOPHAGUS	ESCA
hsa-miR-145-5p	-1.366	0.060	TE9_OESOPHAGUS	ESCA
hsa-miR-145-5p	-1.401	0.066	TE4_OESOPHAGUS	ESCA
hsa-miR-145-5p	-1.401	0.066	TE4_OESOPHAGUS	ESCA
hsa-miR-145-5p	-1.389	0.068	KYSE510_OESOPHAGUS	ESCA
hsa-miR-145-5p	-1.389	0.068	KYSE510_OESOPHAGUS	ESCA
hsa-miR-145-5p	-1.400	0.071	TE6_OESOPHAGUS	ESCA
hsa-miR-145-5p	-1.400	0.071	TE6_OESOPHAGUS	ESCA
hsa-miR-145-5p	-1.355	0.087	TE11_OESOPHAGUS	ESCA
hsa-miR-145-5p	-1.355	0.087	TE11_OESOPHAGUS	ESCA
hsa-miR-145-5p	-1.511	0.044	SKHEP1_LIVER	LIHC
hsa-miR-145-5p	-1.511	0.044	SKHEP1_LIVER	LIHC
hsa-miR-145-5p	-1.482	0.054	SNU886_LIVER	LIHC
hsa-miR-145-5p	-1.482	0.054	SNU886_LIVER	LIHC
hsa-miR-145-5p	-1.459	0.054	JHH1_LIVER	LIHC
hsa-miR-145-5p	-1.459	0.054	JHH1_LIVER	LIHC
hsa-miR-145-5p	-1.479	0.056	SNU182_LIVER	LIHC
hsa-miR-145-5p	-1.479	0.056	SNU182_LIVER	LIHC
hsa-miR-145-5p	-1.441	0.026	NCIH2122_LUNG	LUAD
hsa-miR-145-5p	-1.441	0.026	NCIH2122_LUNG	LUAD

hsa-miR-145-5p	-1.433	0.032	HCC2450_LUNG	LUAD
hsa-miR-145-5p	-1.433	0.032	HCC2450_LUNG	LUAD
hsa-miR-145-5p	-1.435	0.035	RERFLCAD1_LUNG	LUAD
hsa-miR-145-5p	-1.435	0.035	RERFLCAD1_LUNG	LUAD
hsa-miR-145-5p	-1.434	0.040	HCC827_LUNG	LUAD
hsa-miR-145-5p	-1.434	0.040	HCC827_LUNG	LUAD
hsa-miR-145-5p	-1.412	0.045	NCIH441_LUNG	LUAD
hsa-miR-145-5p	-1.412	0.045	NCIH441_LUNG	LUAD
hsa-miR-145-5p	-1.399	0.045	NCIH2087_LUNG	LUAD
hsa-miR-145-5p	-1.399	0.045	NCIH2087_LUNG	LUAD
hsa-miR-145-5p	-1.381	0.052	NCIH838_LUNG	LUAD
hsa-miR-145-5p	-1.381	0.052	NCIH838_LUNG	LUAD
hsa-miR-145-5p	-1.404	0.052	HCC2429_LUNG	LUAD
hsa-miR-145-5p	-1.404	0.052	HCC2429_LUNG	LUAD
hsa-miR-145-5p	-1.497	0.052	NCIH1573_LUNG	LUAD
hsa-miR-145-5p	-1.497	0.052	NCIH1573_LUNG	LUAD
hsa-miR-145-5p	-1.410	0.052	NCIH3122_LUNG	LUAD
hsa-miR-145-5p	-1.410	0.052	NCIH3122_LUNG	LUAD
hsa-miR-145-5p	-1.365	0.057	NCIH2023_LUNG	LUAD
hsa-miR-145-5p	-1.365	0.057	NCIH2023_LUNG	LUAD
hsa-miR-145-5p	-1.401	0.058	NCIH1648_LUNG	LUAD
hsa-miR-145-5p	-1.401	0.058	NCIH1648_LUNG	LUAD
hsa-miR-145-5p	-1.391	0.059	A549_LUNG	LUAD
hsa-miR-145-5p	-1.391	0.059	A549_LUNG	LUAD
hsa-miR-145-5p	-1.380	0.060	NCIH2291_LUNG	LUAD
hsa-miR-145-5p	-1.380	0.060	NCIH2291_LUNG	LUAD
hsa-miR-145-5p	-1.378	0.061	HOP62_LUNG	LUAD
hsa-miR-145-5p	-1.378	0.061	HOP62_LUNG	LUAD
hsa-miR-145-5p	-1.407	0.063	NCIH1568_LUNG	LUAD
hsa-miR-145-5p	-1.407	0.063	NCIH1568_LUNG	LUAD
hsa-miR-145-5p	-1.352	0.065	PC14_LUNG	LUAD
hsa-miR-145-5p	-1.352	0.065	PC14_LUNG	LUAD
hsa-miR-145-5p	-1.355	0.065	NCIH2126_LUNG	LUAD
hsa-miR-145-5p	-1.355	0.065	NCIH2126_LUNG	LUAD
hsa-miR-145-5p	-1.401	0.066	NCIH1693_LUNG	LUAD
hsa-miR-145-5p	-1.401	0.066	NCIH1693_LUNG	LUAD
hsa-miR-145-5p	-1.353	0.071	HCC461_LUNG	LUAD
hsa-miR-145-5p	-1.353	0.071	HCC461_LUNG	LUAD
hsa-miR-145-5p	-1.363	0.072	NCIH2030_LUNG	LUAD
hsa-miR-145-5p	-1.363	0.072	NCIH2030_LUNG	LUAD
hsa-miR-145-5p	-1.356	0.074	EKVX_LUNG	LUAD
hsa-miR-145-5p	-1.356	0.074	EKVX_LUNG	LUAD
hsa-miR-145-5p	-1.370	0.075	NCIH1792_LUNG	LUAD

hsa-miR-145-5p	-1.370	0.075	NCIH1792_LUNG	LUAD
hsa-miR-145-5p	-1.338	0.076	LXF289_LUNG	LUAD
hsa-miR-145-5p	-1.338	0.076	LXF289_LUNG	LUAD
hsa-miR-145-5p	-1.335	0.077	NCIH23_LUNG	LUAD
hsa-miR-145-5p	-1.335	0.077	NCIH23_LUNG	LUAD
hsa-miR-145-5p	-1.317	0.093	ABC1_LUNG	LUAD
hsa-miR-145-5p	-1.317	0.093	ABC1_LUNG	LUAD
hsa-miR-145-5p	-1.338	0.098	MORCPR_LUNG	LUAD
hsa-miR-145-5p	-1.338	0.098	MORCPR_LUNG	LUAD
hsa-miR-145-5p	-1.781	0.000	HCC15_LUNG	LUSC
hsa-miR-145-5p	-1.781	0.000	HCC15_LUNG	LUSC
hsa-miR-145-5p	-1.614	0.001	LK2_LUNG	LUSC
hsa-miR-145-5p	-1.614	0.001	LK2_LUNG	LUSC
hsa-miR-145-5p	-1.614	0.001	HARA_LUNG	LUSC
hsa-miR-145-5p	-1.614	0.001	HARA_LUNG	LUSC
hsa-miR-145-5p	-1.587	0.001	RERFLCAI_LUNG	LUSC
hsa-miR-145-5p	-1.587	0.001	RERFLCAI_LUNG	LUSC
hsa-miR-145-5p	-1.604	0.001	NCIH1703_LUNG	LUSC
hsa-miR-145-5p	-1.604	0.001	NCIH1703_LUNG	LUSC
hsa-miR-145-5p	-1.627	0.001	NCIH520_LUNG	LUSC
hsa-miR-145-5p	-1.627	0.001	NCIH520_LUNG	LUSC
hsa-miR-145-5p	-1.606	0.002	NCIH2170_LUNG	LUSC
hsa-miR-145-5p	-1.606	0.002	NCIH2170_LUNG	LUSC
hsa-miR-145-5p	-1.582	0.002	KNS62_LUNG	LUSC
hsa-miR-145-5p	-1.582	0.002	KNS62_LUNG	LUSC
hsa-miR-145-5p	-1.580	0.002	EPLC272H_LUNG	LUSC
hsa-miR-145-5p	-1.580	0.002	EPLC272H_LUNG	LUSC
hsa-miR-145-5p	-1.600	0.002	SKMES1_LUNG	LUSC
hsa-miR-145-5p	-1.600	0.002	SKMES1_LUNG	LUSC
hsa-miR-145-5p	-1.598	0.003	HCC95_LUNG	LUSC
hsa-miR-145-5p	-1.598	0.003	HCC95_LUNG	LUSC
hsa-miR-145-5p	-1.576	0.003	CALU1_LUNG	LUSC
hsa-miR-145-5p	-1.576	0.003	CALU1_LUNG	LUSC
hsa-miR-145-5p	-1.524	0.003	LUDLU1_LUNG	LUSC
hsa-miR-145-5p	-1.524	0.003	LUDLU1_LUNG	LUSC
hsa-miR-145-5p	-1.589	0.001	GSU_STOMACH	STAD
hsa-miR-145-5p	-1.589	0.001	GSU_STOMACH	STAD
hsa-miR-145-5p	-1.623	0.001	GSS_STOMACH	STAD
hsa-miR-145-5p	-1.623	0.001	GSS_STOMACH	STAD
hsa-miR-145-5p	-1.543	0.002	SNU719_STOMACH	STAD
hsa-miR-145-5p	-1.543	0.002	SNU719_STOMACH	STAD
hsa-miR-145-5p	-1.552	0.002	MKN74_STOMACH	STAD
hsa-miR-145-5p	-1.552	0.002	MKN74_STOMACH	STAD

hsa-miR-145-5p	-1.533	0.003	HS746T_STOMACH	STAD
hsa-miR-145-5p	-1.533	0.003	HS746T_STOMACH	STAD
hsa-miR-145-5p	-1.555	0.003	SH10TC_STOMACH	STAD
hsa-miR-145-5p	-1.555	0.003	SH10TC_STOMACH	STAD
hsa-miR-145-5p	-1.554	0.003	MKN45_STOMACH	STAD
hsa-miR-145-5p	-1.554	0.003	MKN45_STOMACH	STAD
hsa-miR-145-5p	-1.500	0.004	GCIY_STOMACH	STAD
hsa-miR-145-5p	-1.500	0.004	GCIY_STOMACH	STAD
hsa-miR-145-5p	-1.494	0.004	SNU1_STOMACH	STAD
hsa-miR-145-5p	-1.494	0.004	SNU1_STOMACH	STAD
hsa-miR-145-5p	-1.494	0.004	NUGC3_STOMACH	STAD
hsa-miR-145-5p	-1.494	0.004	NUGC3_STOMACH	STAD
hsa-miR-145-5p	-1.508	0.005	SNU216_STOMACH	STAD
hsa-miR-145-5p	-1.508	0.005	SNU216_STOMACH	STAD
hsa-miR-145-5p	-1.514	0.006	HGC27_STOMACH	STAD
hsa-miR-145-5p	-1.514	0.006	HGC27_STOMACH	STAD
hsa-miR-145-5p	-1.498	0.006	LMSU_STOMACH	STAD
hsa-miR-145-5p	-1.498	0.006	LMSU_STOMACH	STAD
hsa-miR-145-5p	-1.492	0.006	SNU601_STOMACH	STAD
hsa-miR-145-5p	-1.492	0.006	SNU601_STOMACH	STAD
hsa-miR-145-5p	-1.474	0.007	KE39_STOMACH	STAD
hsa-miR-145-5p	-1.474	0.007	KE39_STOMACH	STAD
hsa-miR-145-5p	-1.462	0.008	AGS_STOMACH	STAD
hsa-miR-145-5p	-1.462	0.008	AGS_STOMACH	STAD
hsa-miR-145-5p	-1.479	0.010	NCIN87_STOMACH	STAD
hsa-miR-145-5p	-1.479	0.010	NCIN87_STOMACH	STAD
hsa-miR-145-5p	-1.427	0.010	2313287_STOMACH	STAD
hsa-miR-145-5p	-1.427	0.010	2313287_STOMACH	STAD
hsa-miR-145-5p	-1.432	0.013	FU97_STOMACH	STAD
hsa-miR-145-5p	-1.432	0.013	FU97_STOMACH	STAD
hsa-miR-145-5p	-1.604	0.008	SNU1077_ENDOMETRIUM	UCEC
hsa-miR-145-5p	-1.604	0.008	SNU1077_ENDOMETRIUM	UCEC
hsa-miR-145-5p	-1.477	0.060	SNU685_ENDOMETRIUM	UCEC
hsa-miR-145-5p	-1.477	0.060	SNU685_ENDOMETRIUM	UCEC
hsa-miR-145-5p	-1.445	0.081	HEC265_ENDOMETRIUM	UCEC
hsa-miR-145-5p	-1.445	0.081	HEC265_ENDOMETRIUM	UCEC

[$]FDR (false discovery rate) adjusted gene set enrichment *P*-value.

Supplementary Table 5. Twenty-six miRNA whose two strands had differential expression in five or more cancer types compared to corresponding normal tissue samples

miRNA ID (5p)	Fold-change (log2)	Adjusted $P^{\$}$	miRNA ID (3p)	logFC	Adjusted $P^{\$}$	Cancer type
miR-106b-5p	1.4350646	2.41E-06	miR-106b-3p	1.4382706	1.21E-06	ESCA
miR-106b-5p	1.0221583	5.23E-42	miR-106b-3p	1.5462451	1.64E-74	KIRC
miR-106b-5p	1.0679386	1.91E-18	miR-106b-3p	1.3818918	7.66E-19	KIRP
miR-106b-5p	1.0625192	1.69E-22	miR-106b-3p	0.7513792	2.53E-10	LIHC
miR-106b-5p	0.8991272	6.30E-09	miR-106b-3p	0.9885712	3.45E-10	STAD
miR-106b-5p	1.6389015	2.99E-22	miR-106b-3p	1.4904366	6.53E-15	UCEC
miR-126-5p	-1.1544107	3.17E-45	miR-126-3p	-1.0783017	1.40E-33	BRCA
miR-126-5p	-0.6529955	0.0160385	miR-126-3p	-0.8518603	0.0036757	CHOL
miR-126-5p	0.7422276	1.13E-13	miR-126-3p	0.6042148	4.85E-08	KIRC
miR-126-5p	-1.6898659	1.04E-30	miR-126-3p	-2.1340603	9.63E-43	KIRP
miR-126-5p	-2.1135026	2.40E-101	miR-126-3p	-1.7111729	1.95E-47	LUSC
miR-126-5p	-1.3303148	3.45E-17	miR-126-3p	-1.3458899	7.02E-22	UCEC
miR-130b-5p	1.2085548	2.31E-11	miR-130b-3p	1.1022325	1.86E-08	KIRP
miR-130b-5p	0.7827497	3.36E-05	miR-130b-3p	1.2775847	2.97E-11	LIHC
miR-130b-5p	2.5948807	9.30E-45	miR-130b-3p	2.4905608	2.36E-41	LUSC
miR-130b-5p	0.8380271	1.83E-08	miR-130b-3p	0.7136073	9.03E-09	PRAD
miR-130b-5p	2.473476	1.95E-19	miR-130b-3p	2.6463079	2.45E-20	UCEC
miR-139-5p	-2.7219732	4.30E-15	miR-139-3p	-2.3939159	1.07E-12	CHOL
miR-139-5p	-2.2780028	3.26E-22	miR-139-3p	-1.4776257	3.05E-06	ESCA
miR-139-5p	-1.4006569	3.86E-21	miR-139-3p	-1.849103	6.32E-40	LIHC
miR-139-5p	-3.0810298	4.94E-64	miR-139-3p	-4.5920619	2.50E-165	LUAD
miR-139-5p	-0.688115	5.70E-12	miR-139-3p	-0.8303581	6.28E-12	PRAD
miR-141-5p	1.828579	2.20E-07	miR-141-3p	3.0507435	1.12E-12	BLCA
miR-141-5p	1.943777	7.16E-47	miR-141-3p	2.5562106	1.95E-72	BRCA
miR-141-5p	4.8374939	0.0004586	miR-141-3p	4.8715279	0.000277	CHOL
miR-141-5p	2.6026206	7.31E-10	miR-141-3p	2.4583065	9.74E-10	KICH
miR-141-5p	2.2569015	6.93E-16	miR-141-3p	2.0255875	1.32E-11	LUAD
miR-141-5p	1.4971115	2.26E-17	miR-141-3p	1.9946509	7.59E-23	LUSC
miR-141-5p	0.6981136	6.54E-12	miR-141-3p	1.3284283	2.98E-21	PRAD
miR-141-5p	1.1577189	4.44E-07	miR-141-3p	0.5963416	0.012619	STAD
miR-141-5p	2.8680957	6.44E-21	miR-141-3p	3.1811041	1.35E-22	UCEC
miR-142-5p	1.399369	2.94E-19	miR-142-3p	1.7217861	5.83E-24	BRCA
miR-142-5p	0.9540906	0.008899	miR-142-3p	0.8133504	0.0353184	ESCA
miR-142-5p	1.6170961	7.43E-27	miR-142-3p	1.7766809	2.04E-25	KIRC
miR-142-5p	1.0077766	3.43E-05	miR-142-3p	1.1194058	4.06E-06	KIRP
miR-142-5p	-0.8937416	3.95E-09	miR-142-3p	-0.8153151	4.21E-08	LIHC
miR-142-5p	-0.8060994	1.53E-05	miR-142-3p	-0.800265	2.14E-05	THCA
miR-142-5p	1.2331704	8.67E-05	miR-142-3p	1.3227917	9.22E-06	UCEC
miR-143-5p	-1.2257256	1.97E-07	miR-143-3p	-4.0592735	3.55E-63	BLCA

miR-143-5p	-0.9739678	1.95E-15	miR-143-3p	-0.9460254	9.42E-18	BRCA
miR-143-5p	0.628565	0.0456907	miR-143-3p	0.9448065	0.0014099	CHOL
miR-143-5p	-0.9128993	0.0058017	miR-143-3p	-1.6049979	5.58E-06	ESCA
miR-143-5p	-1.1622572	5.89E-11	miR-143-3p	-1.1430923	3.24E-12	KICH
miR-143-5p	1.6831397	2.67E-08	miR-143-3p	-2.4568708	3.37E-33	LUAD
miR-143-5p	-1.3876455	3.37E-25	miR-143-3p	-1.3163486	2.96E-30	PRAD
miR-143-5p	-1.2986048	5.15E-11	miR-143-3p	-2.0131213	1.91E-22	STAD
miR-143-5p	-2.2334884	2.88E-22	miR-143-3p	-2.8955385	4.24E-36	UCEC
miR-145-5p	-2.4226049	4.32E-21	miR-145-3p	-3.0344581	4.41E-39	BLCA
miR-145-5p	-2.4689628	8.54E-204	miR-145-3p	-1.571854	4.00E-91	BRCA
miR-145-5p	-2.2788997	1.01E-10	miR-145-3p	-2.2584042	5.80E-12	ESCA
miR-145-5p	-1.7767284	6.94E-21	miR-145-3p	-1.4272329	1.88E-16	KICH
miR-145-5p	-2.4464747	9.85E-33	miR-145-3p	-1.8413137	6.22E-19	KIRP
miR-145-5p	-2.467371	3.77E-52	miR-145-3p	-1.4629622	5.45E-20	LUAD
miR-145-5p	-1.1745728	6.01E-24	miR-145-3p	-1.5265778	2.07E-39	LUSC
miR-145-5p	-2.23486	2.32E-28	miR-145-3p	-2.005058	8.59E-27	STAD
miR-145-5p	-3.1816276	3.29E-44	miR-145-3p	-3.0884792	3.21E-39	UCEC
miR-146b-5p	-0.653015	0.0459325	miR-146b-3p	-1.8959574	7.14E-09	CHOL
miR-146b-5p	1.1733348	0.0004183	miR-146b-3p	0.7163255	0.0174965	ESCA
miR-146b-5p	1.6800621	3.86E-17	miR-146b-3p	1.5140806	1.89E-16	KIRC
miR-146b-5p	1.6676679	1.49E-09	miR-146b-3p	1.3014284	1.40E-06	KIRP
miR-146b-5p	1.2631638	4.25E-08	miR-146b-3p	-0.7924303	6.97E-06	LIHC
miR-146b-5p	1.1051362	8.34E-17	miR-146b-3p	0.7824689	1.91E-11	PRAD
miR-146b-5p	1.7476373	3.93E-20	miR-146b-3p	1.2872691	4.88E-14	STAD
miR-146b-5p	5.6809938	1.12E-54	miR-146b-3p	5.9730772	3.92E-58	THCA
miR-146b-5p	0.9463475	0.0002882	miR-146b-3p	1.0499711	0.0006219	UCEC
miR-148a-5p	0.859175	1.67E-10	miR-148a-3p	0.9252903	5.36E-14	BRCA
miR-148a-5p	-2.331486	5.95E-16	miR-148a-3p	-2.6629686	3.59E-23	CHOL
miR-148a-5p	-1.272038	7.92E-05	miR-148a-3p	-2.7372454	1.09E-28	ESCA
miR-148a-5p	1.075091	9.73E-05	miR-148a-3p	1.6868996	1.94E-11	LUAD
miR-148a-5p	1.4419623	4.68E-05	miR-148a-3p	0.6894383	0.0319126	UCEC
miR-17-5p	2.0586917	3.25E-14	miR-17-3p	1.0750923	1.18E-07	BLCA
miR-17-5p	0.9642854	9.69E-13	miR-17-3p	1.0491084	4.21E-16	BRCA
miR-17-5p	1.41247	3.66E-06	miR-17-3p	0.9260682	0.0005838	ESCA
miR-17-5p	0.7881893	1.12E-08	miR-17-3p	0.8545704	1.68E-11	KIRP
miR-17-5p	1.2774386	2.86E-13	miR-17-3p	0.9008288	5.61E-09	LIHC
miR-17-5p	1.7031728	2.06E-11	miR-17-3p	0.5920956	0.0052149	LUAD
miR-17-5p	1.4299623	1.54E-34	miR-17-3p	0.6124223	2.27E-12	PRAD
miR-17-5p	1.4472059	1.13E-11	miR-17-3p	0.9897953	1.23E-08	STAD
miR-17-5p	1.8174999	5.34E-19	miR-17-3p	1.0544915	1.76E-07	UCEC
miR-181a-5p	0.8865552	2.51E-05	miR-181a-3p	0.9251543	0.0001101	BLCA
miR-181a-5p	0.8037265	1.30E-12	miR-181a-3p	1.2558223	3.31E-27	BRCA
miR-181a-5p	1.0483608	8.80E-05	miR-181a-3p	1.5007161	1.55E-07	ESCA

miR-181a-5p	-0.7099349	3.65E-07	miR-181a-3p	-1.0619982	5.89E-11	KICH
miR-181a-5p	1.1625399	7.25E-29	miR-181a-3p	1.1442988	5.99E-33	KIRC
miR-181a-5p	-1.178369	4.65E-13	miR-181a-3p	0.6958924	0.0001931	LUAD
miR-181a-5p	-1.4569803	4.86E-43	miR-181a-3p	-1.1982362	8.52E-28	LUSC
miR-181a-5p	0.9658209	1.56E-10	miR-181a-3p	1.2845534	9.63E-17	STAD
miR-181a-5p	1.4319724	2.44E-42	miR-181a-3p	1.0456578	2.22E-21	THCA
miR-181a-5p	0.8710543	9.20E-05	miR-181a-3p	1.1693008	0.0004042	UCEC
miR-181c-5p	0.8288558	1.58E-10	miR-181c-3p	1.4252994	3.65E-29	BRCA
miR-181c-5p	2.0498388	1.77E-07	miR-181c-3p	1.660257	1.21E-05	CHOL
miR-181c-5p	-1.1163229	4.18E-10	miR-181c-3p	-1.2820734	1.28E-13	KICH
miR-181c-5p	1.0184114	4.72E-12	miR-181c-3p	0.6747307	6.12E-07	KIRC
miR-181c-5p	1.3158439	3.30E-10	miR-181c-3p	1.5465457	1.51E-13	KIRP
miR-181c-5p	0.6513472	0.0268373	miR-181c-3p	2.0013724	6.40E-09	UCEC
miR-199a-5p	-2.2025102	1.09E-12	miR-199a-3p	-2.1691149	1.67E-14	KICH
miR-199a-5p	-1.3079078	3.03E-22	miR-199a-3p	-1.3115758	4.52E-25	KIRC
miR-199a-5p	-1.7609211	8.56E-14	miR-199a-3p	-2.0417865	9.26E-19	KIRP
miR-199a-5p	-0.8043185	0.0002509	miR-199a-3p	-1.2562813	8.21E-11	LIHC
miR-199a-5p	1.1522196	9.24E-07	miR-199a-3p	1.0957725	5.58E-07	LUAD
miR-199a-5p	-1.2898736	5.22E-07	miR-199a-3p	-1.3486616	9.95E-10	UCEC
miR-200a-5p	1.926278	1.69E-06	miR-200a-3p	2.5614452	2.85E-08	BLCA
miR-200a-5p	2.5253695	2.05E-07	miR-200a-3p	2.7897192	6.46E-09	CHOL
miR-200a-5p	-1.2381889	4.27E-30	miR-200a-3p	-1.2015973	5.15E-24	KIRC
miR-200a-5p	1.7837488	4.93E-21	miR-200a-3p	1.3976865	1.45E-12	LUSC
miR-200a-5p	1.2639341	7.79E-08	miR-200a-3p	0.9358246	0.0001931	STAD
miR-200a-5p	0.9156059	1.48E-06	miR-200a-3p	0.7496937	8.45E-05	THCA
miR-200a-5p	3.908819	2.51E-32	miR-200a-3p	3.6853797	2.15E-26	UCEC
miR-20a-5p	-0.6008683	0.0287428	miR-20a-3p	-0.8289563	0.0066451	CHOL
miR-20a-5p	1.1949631	5.30E-05	miR-20a-3p	0.8103433	0.0085838	ESCA
miR-20a-5p	0.8646827	6.07E-06	miR-20a-3p	1.6826978	6.14E-21	LUSC
miR-20a-5p	1.5943794	4.12E-32	miR-20a-3p	1.478421	8.38E-22	PRAD
miR-20a-5p	1.3145501	7.19E-10	miR-20a-3p	1.1514326	0.0005076	UCEC
miR-21-5p	1.4750957	3.97E-11	miR-21-3p	0.9412046	0.0005542	BLCA
miR-21-5p	2.2939377	7.90E-101	miR-21-3p	2.1692083	2.10E-62	BRCA
miR-21-5p	2.5788096	5.95E-16	miR-21-3p	0.6981861	0.0432839	CHOL
miR-21-5p	1.3859223	8.68E-08	miR-21-3p	1.3550912	1.14E-05	ESCA
miR-21-5p	2.2911864	4.18E-66	miR-21-3p	1.5975907	1.98E-32	KIRC
miR-21-5p	3.3064889	1.74E-61	miR-21-3p	2.3103081	8.20E-27	KIRP
miR-21-5p	2.7442033	6.50E-30	miR-21-3p	2.2884061	9.65E-22	LUAD
miR-21-5p	0.7848591	8.55E-10	miR-21-3p	0.6216604	1.06E-05	LUSC
miR-21-5p	1.8356011	2.14E-37	miR-21-3p	0.9988783	5.19E-09	STAD
miR-21-5p	1.0559764	3.47E-07	miR-21-3p	1.6901792	2.52E-09	UCEC
miR-27b-5p	-0.9142111	3.21E-07	miR-27b-3p	-1.0849946	1.07E-10	BLCA
miR-27b-5p	-0.838005	1.09E-07	miR-27b-3p	-0.8386381	5.07E-09	KICH

miR-27b-5p	-0.654704	3.91E-17	miR-27b-3p	-0.6934731	3.91E-17	KIRC
miR-27b-5p	0.7131826	8.34E-17	miR-27b-3p	0.6468819	1.71E-15	THCA
miR-27b-5p	-1.455241	9.32E-20	miR-27b-3p	-1.4244126	1.36E-18	UCEC
miR-28-5p	-1.2550279	9.62E-23	miR-28-3p	-1.785152	2.62E-38	BLCA
miR-28-5p	-0.8267367	4.05E-05	miR-28-3p	-1.3802502	9.65E-13	ESCA
miR-28-5p	0.7153271	1.21E-12	miR-28-3p	0.9131786	8.66E-13	KIRP
miR-28-5p	-0.9565799	7.36E-19	miR-28-3p	-1.0580646	8.57E-19	STAD
miR-28-5p	-1.2924294	6.60E-22	miR-28-3p	-1.3049041	4.79E-21	UCEC
miR-29c-5p	-1.5636362	8.02E-15	miR-29c-3p	-1.8147061	1.02E-10	ESCA
miR-29c-5p	-0.950019	4.25E-13	miR-29c-3p	-1.3586316	7.54E-30	KIRP
miR-29c-5p	-0.6144722	3.58E-07	miR-29c-3p	-0.8476849	2.51E-10	LIHC
miR-29c-5p	-0.8520088	2.73E-16	miR-29c-3p	-1.4260156	1.86E-21	LUSC
miR-29c-5p	-1.3399625	8.59E-27	miR-29c-3p	-1.6186078	1.73E-27	STAD
miR-29c-5p	-0.9529398	4.34E-09	miR-29c-3p	-1.4945576	1.56E-12	UCEC
miR-30a-5p	-1.8395418	4.41E-17	miR-30a-3p	-3.0696195	4.23E-52	BLCA
miR-30a-5p	-1.9120846	6.41E-12	miR-30a-3p	-2.0374773	6.18E-14	ESCA
miR-30a-5p	-0.7460319	1.59E-16	miR-30a-3p	-0.9077712	2.04E-21	KIRC
miR-30a-5p	-1.4671107	3.46E-12	miR-30a-3p	-2.6850065	3.76E-48	LUAD
miR-30a-5p	-3.0376136	8.57E-119	miR-30a-3p	-2.8107847	2.50E-80	LUSC
miR-30a-5p	-1.3098225	4.84E-15	miR-30a-3p	-1.8435354	1.32E-25	STAD
miR-324-5p	1.7212211	1.76E-09	miR-324-3p	1.0564731	0.0002427	BLCA
miR-324-5p	0.7497825	0.0028384	miR-324-3p	0.6205604	0.0475336	ESCA
miR-324-5p	0.8499693	1.25E-10	miR-324-3p	0.7616481	1.60E-08	LIHC
miR-324-5p	1.0841074	3.53E-14	miR-324-3p	0.7815604	5.27E-07	LUSC
miR-324-5p	1.5580778	1.01E-13	miR-324-3p	2.0027054	1.65E-14	UCEC
miR-378a-5p	-0.9881905	1.90E-06	miR-378a-3p	-1.6249396	1.03E-11	BLCA
miR-378a-5p	-2.5699553	7.51E-176	miR-378a-3p	-2.1625627	2.08E-85	BRCA
miR-378a-5p	-2.6199628	2.48E-13	miR-378a-3p	-2.9267298	5.95E-16	CHOL
miR-378a-5p	-2.3493663	5.84E-54	miR-378a-3p	-2.7203019	5.90E-62	LUAD
miR-378a-5p	-0.909329	3.78E-19	miR-378a-3p	-0.8490751	3.01E-14	PRAD
miR-378a-5p	-1.1285713	3.26E-17	miR-378a-3p	-1.1288917	3.80E-12	STAD
miR-425-5p	1.8926374	3.46E-12	miR-425-3p	1.2746575	2.43E-06	BLCA
miR-425-5p	0.7604611	1.81E-08	miR-425-3p	0.9335463	4.06E-19	BRCA
miR-425-5p	0.7333947	2.59E-05	miR-425-3p	0.5999791	9.34E-06	KIRP
miR-425-5p	1.3369204	5.09E-23	miR-425-3p	-0.7747563	1.21E-16	PRAD
miR-425-5p	2.8982325	1.78E-24	miR-425-3p	1.7206028	3.93E-15	UCEC
miR-708-5p	2.2754987	1.30E-08	miR-708-3p	1.8339948	1.52E-06	BLCA
miR-708-5p	0.780295	3.32E-11	miR-708-3p	0.6082149	1.48E-07	BRCA
miR-708-5p	1.5656254	0.0101809	miR-708-3p	0.9559648	0.0278204	ESCA
miR-708-5p	0.8469081	1.38E-06	miR-708-3p	1.0006363	8.91E-10	KIRC
miR-708-5p	3.4986897	1.95E-47	miR-708-3p	2.8262668	1.35E-35	LUSC
miR-93-5p	1.8255399	2.23E-12	miR-93-3p	1.6285818	1.74E-11	BLCA
miR-93-5p	1.1785645	1.42E-25	miR-93-3p	0.8188716	1.44E-12	BRCA

miR-93-5p	0.762727	0.0056077	miR-93-3p	0.8746458	0.0100853	CHOL
miR-93-5p	1.4705525	1.43E-06	miR-93-3p	1.5081936	7.69E-07	ESCA
miR-93-5p	1.5570948	1.40E-21	miR-93-3p	0.6384779	2.22E-07	KIRP
miR-93-5p	1.7911258	2.22E-34	miR-93-3p	0.7863038	1.23E-11	LIHC
miR-93-5p	1.2700334	9.28E-14	miR-93-3p	1.7591761	1.18E-30	LUSC
miR-93-5p	1.6897189	6.51E-48	miR-93-3p	0.9911991	1.61E-15	PRAD
miR-93-5p	1.3319593	2.38E-12	miR-93-3p	0.7718685	4.40E-06	STAD
miR-93-5p	2.1758801	1.77E-28	miR-93-3p	2.0254167	1.87E-14	UCEC

$^{\$}$Listed miRNA have at least a 1.5 fold-change with adjusted *P*-value <0.05. *P*-values were adjusted using the Benjamini-Hochberg (1995)[1] multiple testing correction method.

Supplementary Table 6. Pathways significantly enriched with potential targets of miRNA-5p/3p pairs

5p/3p pair	KEGG Pathway	Enrichment *P (*FDR)[$]	miR-5p/-3p - pathway average correlation	Cancer type	Score[#]
miR-130b	Adherens junction	0.042	-0.245	LUSC	0.300
miR-130b	Adherens junction	0.011	-0.146	UCEC	0.444
miR-130b	AGE-RAGE signaling pathway in diabetic complications	0.046	-0.264	LUSC	0.250
miR-130b	AGE-RAGE signaling pathway in diabetic complications	0.004	-0.153	UCEC	0.083
miR-130b	Aldosterone synthesis and secretion	0.010	-0.249	LUSC	0.083
miR-130b	Aldosterone synthesis and secretion	0.007	-0.134	UCEC	0.200
miR-130b	Aldosterone-regulated sodium reabsorption	0.019	-0.118	UCEC	0.167
miR-130b	Amphetamine addiction	0.007	-0.141	UCEC	0.222
miR-130b	cAMP signaling pathway	0.026	-0.109	UCEC	0.267
miR-130b	Cell adhesion molecules (CAMs)	0.002	-0.346	LUSC	0.158
miR-130b	cGMP-PKG signaling pathway	0.005	-0.253	LUSC	0.150
miR-130b	cGMP-PKG signaling pathway	0.000	-0.143	UCEC	0.045
miR-130b	Cholinergic synapse	0.014	-0.139	UCEC	0.455
miR-130b	Circadian entrainment	0.007	-0.146	UCEC	0.455
miR-130b	Dilated cardiomyopathy	0.011	-0.108	UCEC	0.000
miR-130b	Dopaminergic synapse	0.036	-0.144	UCEC	0.455
miR-130b	EGFR tyrosine kinase inhibitor resistance	0.046	-0.098	UCEC	0.375
miR-130b	Endocrine resistance	0.007	-0.118	UCEC	0.000
miR-130b	Endocytosis	0.007	-0.147	UCEC	0.000
miR-130b	Estrogen signaling pathway	0.046	-0.121	UCEC	0.000
miR-130b	Focal adhesion	0.000	-0.103	UCEC	0.111
miR-130b	FoxO signaling pathway	0.002	-0.191	UCEC	0.000
miR-130b	GABAergic synapse	0.025	-0.160	UCEC	0.444
miR-130b	Gap junction	0.025	-0.127	UCEC	0.333
miR-130b	GnRH signaling pathway	0.012	-0.129	UCEC	0.100
miR-130b	Hematopoietic cell lineage	0.013	-0.331	LUSC	0.231
miR-130b	HTLV-I infection	0.000	-0.293	LUSC	0.467
miR-130b	HTLV-I infection	0.000	-0.143	UCEC	0.240
miR-130b	Inflammatory mediator regulation of TRP channels	0.007	-0.160	UCEC	0.273
miR-130b	Leishmaniasis	0.002	-0.309	LUSC	0.077
miR-130b	Longevity regulating pathway	0.036	-0.126	UCEC	0.000
miR-130b	Longevity regulating pathway - multiple species	0.014	-0.129	UCEC	0.125
miR-130b	MAPK signaling pathway	0.007	-0.164	UCEC	0.250
miR-130b	MicroRNAs in cancer	0.037	-0.266	LUSC	0.125
miR-130b	MicroRNAs in cancer	0.007	-0.108	UCEC	0.071
miR-130b	Morphine addiction	0.002	-0.137	UCEC	0.250

miR-130b	Osteoclast differentiation	0.005	-0.268	LUSC	0.059
miR-130b	Oxytocin signaling pathway	0.005	-0.253	LUSC	0.263
miR-130b	Oxytocin signaling pathway	0.000	-0.138	UCEC	0.053
miR-130b	Pathways in cancer	0.013	-0.299	LUSC	0.176
miR-130b	Pathways in cancer	0.007	-0.157	UCEC	0.111
miR-130b	Pertussis	0.046	-0.304	LUSC	0.200
miR-130b	PI3K-Akt signaling pathway	0.005	-0.123	UCEC	0.040
miR-130b	Proteoglycans in cancer	0.000	-0.126	UCEC	0.273
miR-130b	Rap1 signaling pathway	0.000	-0.251	LUSC	0.259
miR-130b	Rap1 signaling pathway	0.021	-0.115	UCEC	0.000
miR-130b	Ras signaling pathway	0.003	-0.123	UCEC	0.250
miR-130b	Regulation of actin cytoskeleton	0.046	-0.099	UCEC	0.267
miR-130b	Regulation of lipolysis in adipocytes	0.025	-0.112	UCEC	0.000
miR-130b	Renin secretion	0.046	-0.135	UCEC	0.286
miR-130b	Retrograde endocannabinoid signaling	0.048	-0.147	UCEC	0.444
miR-130b	Rheumatoid arthritis	0.000	-0.317	LUSC	0.313
miR-130b	Staphylococcus aureus infection	0.000	-0.370	LUSC	0.000
miR-130b	Tight junction	0.002	-0.174	LUSC	0.263
miR-130b	TNF signaling pathway	0.001	-0.246	LUSC	0.000
miR-130b	Transcriptional misregulation in cancer	0.012	-0.152	UCEC	0.133
miR-130b	Vascular smooth muscle contraction	0.005	-0.266	LUSC	0.125
miR-130b	Vascular smooth muscle contraction	0.000	-0.130	UCEC	0.105
miR-130b	Viral myocarditis	0.003	-0.311	LUSC	0.000
miR-139	Cell cycle	0.040	-0.415	LIHC	0.143
miR-139	Cell cycle	0.000	-0.166	ESCA	0.300
miR-139	Chronic myeloid leukemia	0.006	-0.208	CHOL	0.000
miR-139	DNA replication	0.000	-0.166	ESCA	0.286
miR-139	Endocrine resistance	0.011	-0.202	CHOL	0.091
miR-139	ErbB signaling pathway	0.037	-0.180	CHOL	0.111
miR-139	Focal adhesion	0.011	-0.170	CHOL	0.118
miR-139	Hepatitis B	0.037	-0.217	CHOL	0.000
miR-139	Melanoma	0.037	-0.216	CHOL	0.125
miR-139	MicroRNAs in cancer	0.002	-0.237	CHOL	0.125
miR-139	Pathways in cancer	0.000	-0.201	CHOL	0.065
miR-139	Prostate cancer	0.037	-0.215	CHOL	0.000
miR-139	Small cell lung cancer	0.011	-0.180	CHOL	0.000
miR-141	Adherens junction	0.028	-0.268	LUSC	0.333
miR-141	Adherens junction	0.001	-0.197	UCEC	0.200
miR-141	AGE-RAGE signaling pathway in diabetic complications	0.014	-0.229	UCEC	0.222
miR-141	AGE-RAGE signaling pathway in diabetic complications	0.027	-0.185	LUAD	0.125
miR-141	Aldosterone synthesis and secretion	0.040	-0.210	UCEC	0.143
miR-141	Aldosterone synthesis and secretion	0.045	-0.206	BLCA	0.167

miR-141	Amphetamine addiction	0.007	-0.284	STAD	0.286
miR-141	Amphetamine addiction	0.024	-0.154	BLCA	0.167
miR-141	Apoptosis	0.050	-0.260	LUSC	0.250
miR-141	Arrhythmogenic right ventricular cardiomyopathy (ARVC)	0.000	-0.402	PRAD	0.111
miR-141	Axon guidance	0.001	-0.275	BLCA	0.231
miR-141	Axon guidance	0.043	-0.174	UCEC	0.364
miR-141	Calcium signaling pathway	0.013	-0.297	BLCA	0.364
miR-141	Calcium signaling pathway	0.022	-0.218	UCEC	0.417
miR-141	cAMP signaling pathway	0.044	-0.404	STAD	0.100
miR-141	cAMP signaling pathway	0.024	-0.239	BLCA	0.091
miR-141	cAMP signaling pathway	0.040	-0.186	UCEC	0.333
miR-141	Cell adhesion molecules (CAMs)	0.016	-0.371	LUSC	0.214
miR-141	Cell adhesion molecules (CAMs)	0.020	-0.339	PRAD	0.222
miR-141	Cell adhesion molecules (CAMs)	0.002	-0.228	LUAD	0.000
miR-141	cGMP-PKG signaling pathway	0.006	-0.522	STAD	0.455
miR-141	cGMP-PKG signaling pathway	0.047	-0.270	LUSC	0.286
miR-141	cGMP-PKG signaling pathway	0.000	-0.177	UCEC	0.300
miR-141	Chagas disease (American trypanosomiasis)	0.050	-0.281	LUSC	0.000
miR-141	Cholinergic synapse	0.006	-0.291	BLCA	0.333
miR-141	Chronic myeloid leukemia	0.030	-0.291	BLCA	0.333
miR-141	Circadian entrainment	0.006	-0.428	STAD	0.111
miR-141	Circadian entrainment	0.009	-0.248	BLCA	0.000
miR-141	Circadian entrainment	0.005	-0.199	UCEC	0.300
miR-141	Cocaine addiction	0.038	-0.227	STAD	0.000
miR-141	Complement and coagulation cascades	0.015	-0.295	CHOL	0.250
miR-141	Dilated cardiomyopathy	0.006	-0.403	PRAD	0.000
miR-141	Dopaminergic synapse	0.011	-0.374	STAD	0.444
miR-141	ECM-receptor interaction	0.004	-0.359	PRAD	0.125
miR-141	Endocrine and other factor-regulated calcium reabsorption	0.021	-0.408	PRAD	0.000
miR-141	Endocrine and other factor-regulated calcium reabsorption	0.006	-0.261	BLCA	0.000
miR-141	Endocrine and other factor-regulated calcium reabsorption	0.014	-0.181	UCEC	0.167
miR-141	Epstein-Barr virus infection	0.025	-0.206	LUAD	0.083
miR-141	Estrogen signaling pathway	0.011	-0.297	BLCA	0.000
miR-141	Focal adhesion	0.000	-0.403	PRAD	0.063
miR-141	Focal adhesion	0.000	-0.370	BLCA	0.048
miR-141	Focal adhesion	0.000	-0.258	UCEC	0.056
miR-141	FoxO signaling pathway	0.000	-0.334	BLCA	0.083
miR-141	FoxO signaling pathway	0.009	-0.205	UCEC	0.000
miR-141	Gap junction	0.006	-0.279	BLCA	0.000
miR-141	Gap junction	0.007	-0.223	UCEC	0.222
miR-141	Glucagon signaling pathway	0.000	-0.229	BLCA	0.091

miR-141	Glutamatergic synapse	0.023	-0.262	BLCA	0.250
miR-141	Hedgehog signaling pathway	0.014	-0.204	UCEC	0.333
miR-141	Hematopoietic cell lineage	0.016	-0.306	LUSC	0.000
miR-141	Hippo signaling pathway	0.021	-0.399	PRAD	0.444
miR-141	HTLV-I infection	0.012	-0.343	LUSC	0.286
miR-141	HTLV-I infection	0.000	-0.340	BLCA	0.111
miR-141	HTLV-I infection	0.000	-0.209	LUAD	0.105
miR-141	HTLV-I infection	0.000	-0.208	UCEC	0.000
miR-141	Hypertrophic cardiomyopathy (HCM)	0.004	-0.403	PRAD	0.000
miR-141	Inflammatory bowel disease (IBD)	0.039	-0.191	LUAD	0.167
miR-141	Inflammatory mediator regulation of TRP channels	0.032	-0.181	UCEC	0.250
miR-141	Insulin secretion	0.049	-0.384	PRAD	0.000
miR-141	Leishmaniasis	0.001	-0.312	LUSC	0.250
miR-141	Leishmaniasis	0.023	-0.214	LUAD	0.286
miR-141	Longevity regulating pathway	0.027	-0.217	BLCA	0.000
miR-141	Longevity regulating pathway - multiple species	0.005	-0.231	BLCA	0.286
miR-141	Long-term potentiation	0.006	-0.222	BLCA	0.429
miR-141	Malaria	0.047	-0.235	LUAD	0.200
miR-141	MAPK signaling pathway	0.000	-0.361	BLCA	0.000
miR-141	MAPK signaling pathway	0.011	-0.229	UCEC	0.250
miR-141	Melanoma	0.027	-0.407	BLCA	0.167
miR-141	MicroRNAs in cancer	0.008	-0.395	PRAD	0.400
miR-141	MicroRNAs in cancer	0.000	-0.352	BLCA	0.385
miR-141	MicroRNAs in cancer	0.047	-0.293	LUSC	0.462
miR-141	MicroRNAs in cancer	0.007	-0.250	UCEC	0.417
miR-141	Neurotrophin signaling pathway	0.032	-0.171	UCEC	0.000
miR-141	Oocyte meiosis	0.030	-0.217	BLCA	0.000
miR-141	Osteoclast differentiation	0.004	-0.335	LUSC	0.200
miR-141	Osteoclast differentiation	0.015	-0.194	LUAD	0.200
miR-141	Oxytocin signaling pathway	0.038	-0.419	STAD	0.000
miR-141	Oxytocin signaling pathway	0.016	-0.261	LUSC	0.267
miR-141	Oxytocin signaling pathway	0.000	-0.240	BLCA	0.158
miR-141	Oxytocin signaling pathway	0.005	-0.195	UCEC	0.231
miR-141	Pancreatic secretion	0.021	-0.374	PRAD	0.429
miR-141	Pathways in cancer	0.021	-0.381	PRAD	0.250
miR-141	Pathways in cancer	0.000	-0.288	BLCA	0.000
miR-141	Pathways in cancer	0.014	-0.226	UCEC	0.143
miR-141	Pertussis	0.004	-0.296	LUSC	0.000
miR-141	Phagosome	0.050	-0.353	LUSC	0.000
miR-141	Phagosome	0.015	-0.221	LUAD	0.091
miR-141	Phospholipase D signaling pathway	0.009	-0.370	BLCA	0.100
miR-141	Phospholipase D signaling pathway	0.006	-0.236	UCEC	0.250

miR-141	PI3K-Akt signaling pathway	0.008	-0.386	BLCA	0.000
miR-141	PI3K-Akt signaling pathway	0.000	-0.337	PRAD	0.000
miR-141	PI3K-Akt signaling pathway	0.007	-0.239	UCEC	0.150
miR-141	Platelet activation	0.050	-0.317	LUSC	0.273
miR-141	Platelet activation	0.000	-0.286	BLCA	0.250
miR-141	Platelet activation	0.006	-0.228	UCEC	0.182
miR-141	Progesterone-mediated oocyte maturation	0.030	-0.228	BLCA	0.143
miR-141	Proteoglycans in cancer	0.044	-0.456	STAD	0.100
miR-141	Proteoglycans in cancer	0.019	-0.410	PRAD	0.182
miR-141	Proteoglycans in cancer	0.000	-0.285	BLCA	0.000
miR-141	Proteoglycans in cancer	0.044	-0.225	LUAD	0.091
miR-141	Proteoglycans in cancer	0.002	-0.191	UCEC	0.063
miR-141	Rap1 signaling pathway	0.002	-0.387	BLCA	0.214
miR-141	Rap1 signaling pathway	0.021	-0.383	PRAD	0.364
miR-141	Rap1 signaling pathway	0.004	-0.342	LUSC	0.200
miR-141	Rap1 signaling pathway	0.027	-0.229	LUAD	0.250
miR-141	Rap1 signaling pathway	0.003	-0.222	UCEC	0.375
miR-141	Ras signaling pathway	0.030	-0.392	PRAD	0.182
miR-141	Ras signaling pathway	0.004	-0.355	BLCA	0.143
miR-141	Ras signaling pathway	0.000	-0.213	UCEC	0.050
miR-141	Regulation of actin cytoskeleton	0.001	-0.411	PRAD	0.143
miR-141	Regulation of actin cytoskeleton	0.000	-0.375	BLCA	0.125
miR-141	Regulation of actin cytoskeleton	0.014	-0.224	UCEC	0.071
miR-141	Renin secretion	0.014	-0.213	UCEC	0.143
miR-141	Retrograde endocannabinoid signaling	0.012	-0.238	BLCA	0.125
miR-141	Rheumatoid arthritis	0.012	-0.354	LUSC	0.364
miR-141	Rheumatoid arthritis	0.042	-0.220	LUAD	0.000
miR-141	Salivary secretion	0.020	-0.176	UCEC	0.125
miR-141	Serotonergic synapse	0.006	-0.365	STAD	0.222
miR-141	Serotonergic synapse	0.022	-0.230	BLCA	0.125
miR-141	Sphingolipid signaling pathway	0.050	-0.274	LUSC	0.182
miR-141	Staphylococcus aureus infection	0.000	-0.385	LUSC	0.231
miR-141	Th17 cell differentiation	0.030	-0.165	LUAD	0.250
miR-141	Tight junction	0.006	-0.521	STAD	0.100
miR-141	Tight junction	0.029	-0.291	LUSC	0.231
miR-141	Tight junction	0.002	-0.204	LUAD	0.167
miR-141	TNF signaling pathway	0.037	-0.195	LUSC	0.182
miR-141	Toxoplasmosis	0.050	-0.351	LUSC	0.091
miR-141	Toxoplasmosis	0.023	-0.212	LUAD	0.444
miR-141	Transcriptional misregulation in cancer	0.047	-0.204	UCEC	0.455
miR-141	Vascular smooth muscle contraction	0.007	-0.550	STAD	0.111
miR-141	Vascular smooth muscle contraction	0.021	-0.442	PRAD	0.125
miR-141	Vascular smooth muscle contraction	0.001	-0.258	BLCA	0.182

miR-141	Vascular smooth muscle contraction	0.050	-0.238	LUSC	0.182
miR-141	Vascular smooth muscle contraction	0.000	-0.223	UCEC	0.143
miR-141	Viral myocarditis	0.010	-0.352	LUSC	0.111
miR-141	Viral myocarditis	0.027	-0.279	LUAD	0.167
miR-141	Wnt signaling pathway	0.020	-0.427	PRAD	0.111
miR-141	Wnt signaling pathway	0.009	-0.260	BLCA	0.400
miR-141	Wnt signaling pathway	0.006	-0.188	UCEC	0.333
miR-143	Cell cycle	0.000	-0.239	STAD	0.235
miR-143	Cell cycle	0.000	-0.142	ESCA	0.421
miR-143	Complement and coagulation cascades	0.000	-0.134	CHOL	0.188
miR-143	Fatty acid metabolism	0.002	-0.156	CHOL	0.400
miR-143	Hepatitis B	0.028	-0.133	ESCA	0.222
miR-143	MicroRNAs in cancer	0.001	-0.182	STAD	0.273
miR-143	Oocyte meiosis	0.049	-0.113	KICH	0.182
miR-143	p53 signaling pathway	0.000	-0.222	STAD	0.200
miR-143	p53 signaling pathway	0.000	-0.117	ESCA	0.250
miR-143	Progesterone-mediated oocyte maturation	0.007	-0.269	STAD	0.250
miR-143	Rap1 signaling pathway	0.024	-0.139	KICH	0.118
miR-145	Biosynthesis of amino acids	0.009	-0.116	LUAD	0.286
miR-145	Cell cycle	0.000	-0.361	STAD	0.118
miR-145	Cell cycle	0.000	-0.300	LUAD	0.077
miR-145	Cell cycle	0.000	-0.230	LUSC	0.200
miR-145	Cell cycle	0.000	-0.172	ESCA	0.053
miR-145	Cell cycle	0.000	-0.097	BLCA	0.067
miR-145	Cell cycle	0.000	-0.077	UCEC	0.143
miR-145	Cysteine and methionine metabolism	0.014	-0.158	LUSC	0.143
miR-145	Fanconi anemia pathway	0.017	-0.174	ESCA	0.000
miR-145	Hepatitis B	0.024	-0.265	STAD	0.429
miR-145	MicroRNAs in cancer	0.026	-0.290	STAD	0.000
miR-145	MicroRNAs in cancer	0.049	-0.139	UCEC	0.182
miR-145	Oocyte meiosis	0.046	-0.418	STAD	0.000
miR-145	Oocyte meiosis	0.021	-0.233	ESCA	0.143
miR-145	p53 signaling pathway	0.001	-0.324	STAD	0.286
miR-145	p53 signaling pathway	0.030	-0.280	LUAD	0.333
miR-145	p53 signaling pathway	0.009	-0.185	LUSC	0.111
miR-145	p53 signaling pathway	0.000	-0.172	ESCA	0.111
miR-145	Pancreatic cancer	0.024	-0.249	STAD	0.200
miR-145	Pathways in cancer	0.024	-0.286	STAD	0.167
miR-145	Pathways in cancer	0.022	-0.133	ESCA	0.231
miR-145	Progesterone-mediated oocyte maturation	0.024	-0.415	STAD	0.000
miR-145	Progesterone-mediated oocyte maturation	0.007	-0.233	ESCA	0.143
miR-145	Small cell lung cancer	0.022	-0.316	STAD	0.000
miR-145	Small cell lung cancer	0.004	-0.135	ESCA	0.000

miR-145	Viral carcinogenesis	0.000	-0.127	ESCA	0.333
miR-146b	cAMP signaling pathway	0.040	-0.126	STAD	0.273
miR-146b	cGMP-PKG signaling pathway	0.001	-0.148	STAD	0.308
miR-146b	Circadian entrainment	0.004	-0.152	STAD	0.333
miR-146b	Oxytocin signaling pathway	0.007	-0.153	STAD	0.091
miR-146b	Vascular smooth muscle contraction	0.001	-0.146	STAD	0.000
miR-148a	AGE-RAGE signaling pathway in diabetic complications	0.009	-0.162	CHOL	0.250
miR-148a	Arrhythmogenic right ventricular cardiomyopathy (ARVC)	0.009	-0.245	CHOL	0.200
miR-148a	Axon guidance	0.034	-0.200	CHOL	0.467
miR-148a	cGMP-PKG signaling pathway	0.002	-0.096	UCEC	0.357
miR-148a	Chronic myeloid leukemia	0.009	-0.206	CHOL	0.400
miR-148a	Dilated cardiomyopathy	0.027	-0.254	CHOL	0.300
miR-148a	EGFR tyrosine kinase inhibitor resistance	0.037	-0.242	CHOL	0.333
miR-148a	HTLV-I infection	0.001	-0.080	UCEC	0.474
miR-148a	Hypertrophic cardiomyopathy (HCM)	0.009	-0.247	CHOL	0.455
miR-148a	Insulin secretion	0.009	-0.161	CHOL	0.182
miR-148a	Longevity regulating pathway - multiple species	0.020	-0.130	UCEC	0.286
miR-148a	Melanoma	0.049	-0.231	CHOL	0.375
miR-148a	Ovarian steroidogenesis	0.021	-0.146	UCEC	0.000
miR-148a	Pancreatic secretion	0.037	-0.168	CHOL	0.100
miR-148a	PI3K-Akt signaling pathway	0.000	-0.193	CHOL	0.194
miR-148a	Protein digestion and absorption	0.000	-0.169	CHOL	0.143
miR-148a	Rap1 signaling pathway	0.034	-0.198	CHOL	0.118
miR-148a	Regulation of actin cytoskeleton	0.009	-0.221	CHOL	0.368
miR-148a	Renal cell carcinoma	0.014	-0.195	CHOL	0.222
miR-148a	Small cell lung cancer	0.001	-0.178	CHOL	0.231
miR-148a	Thyroid hormone signaling pathway	0.048	-0.145	CHOL	0.091
miR-148a	Thyroid hormone synthesis	0.009	-0.197	CHOL	0.100
miR-17	Adherens junction	0.011	-0.147	UCEC	0.100
miR-17	Adrenergic signaling in cardiomyocytes	0.017	-0.315	PRAD	0.083
miR-17	Adrenergic signaling in cardiomyocytes	0.015	-0.261	ESCA	0.077
miR-17	Adrenergic signaling in cardiomyocytes	0.003	-0.181	BLCA	0.133
miR-17	AGE-RAGE signaling pathway in diabetic complications	0.001	-0.193	BLCA	0.154
miR-17	AGE-RAGE signaling pathway in diabetic complications	0.002	-0.167	LUAD	0.077
miR-17	AGE-RAGE signaling pathway in diabetic complications	0.000	-0.156	UCEC	0.438
miR-17	Aldosterone synthesis and secretion	0.048	-0.385	STAD	0.125
miR-17	Aldosterone synthesis and secretion	0.025	-0.277	PRAD	0.250
miR-17	Aldosterone synthesis and secretion	0.017	-0.132	BLCA	0.000
miR-17	Aldosterone synthesis and secretion	0.019	-0.125	UCEC	0.200
miR-17	Aldosterone-regulated sodium reabsorption	0.002	-0.293	PRAD	0.429

miR-17	Aldosterone-regulated sodium reabsorption	0.039	-0.136	UCEC	0.000
miR-17	Amphetamine addiction	0.049	-0.163	BLCA	0.143
miR-17	Arrhythmogenic right ventricular cardiomyopathy (ARVC))	0.000	-0.346	PRAD	0.091
miR-17	Axon guidance	0.000	-0.303	PRAD	0.235
miR-17	Axon guidance	0.000	-0.189	BLCA	0.000
miR-17	Calcium signaling pathway	0.014	-0.231	BLCA	0.333
miR-17	Calcium signaling pathway	0.046	-0.134	UCEC	0.200
miR-17	cAMP signaling pathway	0.025	-0.371	STAD	0.000
miR-17	cAMP signaling pathway	0.022	-0.317	PRAD	0.071
miR-17	cAMP signaling pathway	0.018	-0.244	ESCA	0.000
miR-17	cAMP signaling pathway	0.003	-0.205	BLCA	0.222
miR-17	Carbon metabolism	0.018	-0.136	LIHC	0.400
miR-17	Cell adhesion molecules (CAMs)	0.005	-0.202	LUAD	0.067
miR-17	cGMP-PKG signaling pathway	0.000	-0.503	STAD	0.105
miR-17	cGMP-PKG signaling pathway	0.000	-0.343	PRAD	0.100
miR-17	cGMP-PKG signaling pathway	0.000	-0.311	ESCA	0.000
miR-17	cGMP-PKG signaling pathway	0.000	-0.243	BLCA	0.074
miR-17	cGMP-PKG signaling pathway	0.000	-0.154	UCEC	0.167
miR-17	Chemokine signaling pathway	0.035	-0.212	BLCA	0.143
miR-17	Cholinergic synapse	0.002	-0.173	BLCA	0.154
miR-17	Cholinergic synapse	0.003	-0.141	UCEC	0.214
miR-17	Circadian entrainment	0.000	-0.385	STAD	0.071
miR-17	Circadian entrainment	0.000	-0.169	BLCA	0.154
miR-17	Circadian entrainment	0.008	-0.163	UCEC	0.167
miR-17	Circadian rhythm	0.007	-0.196	BLCA	0.000
miR-17	Dilated cardiomyopathy	0.000	-0.335	PRAD	0.167
miR-17	Dilated cardiomyopathy	0.023	-0.274	ESCA	0.222
miR-17	Dilated cardiomyopathy	0.031	-0.135	UCEC	0.100
miR-17	Dopaminergic synapse	0.034	-0.393	STAD	0.091
miR-17	Dopaminergic synapse	0.035	-0.166	BLCA	0.091
miR-17	Drug metabolism - cytochrome P450	0.034	-0.272	LIHC	0.429
miR-17	EGFR tyrosine kinase inhibitor resistance	0.025	-0.322	PRAD	0.250
miR-17	EGFR tyrosine kinase inhibitor resistance	0.041	-0.122	UCEC	0.222
miR-17	Endocrine and other factor-regulated calcium reabsorption	0.035	-0.163	BLCA	0.167
miR-17	Endocrine resistance	0.017	-0.206	BLCA	0.000
miR-17	Endocrine resistance	0.020	-0.129	UCEC	0.182
miR-17	Estrogen signaling pathway	0.048	-0.383	STAD	0.222
miR-17	Estrogen signaling pathway	0.001	-0.189	BLCA	0.000
miR-17	Focal adhesion	0.000	-0.330	PRAD	0.045
miR-17	Focal adhesion	0.012	-0.293	ESCA	0.125
miR-17	Focal adhesion	0.000	-0.252	BLCA	0.133
miR-17	Focal adhesion	0.000	-0.144	UCEC	0.111

miR-17	FoxO signaling pathway	0.000	-0.238	BLCA	0.059
miR-17	FoxO signaling pathway	0.001	-0.189	UCEC	0.059
miR-17	FoxO signaling pathway	0.034	-0.145	LIHC	0.300
miR-17	GABAergic synapse	0.031	-0.397	STAD	0.000
miR-17	Gap junction	0.008	-0.226	BLCA	0.200
miR-17	Gap junction	0.027	-0.161	UCEC	0.100
miR-17	Gastric acid secretion	0.019	-0.318	PRAD	0.375
miR-17	Glucagon signaling pathway	0.003	-0.147	BLCA	0.083
miR-17	GnRH signaling pathway	0.031	-0.176	BLCA	0.000
miR-17	GnRH signaling pathway	0.014	-0.138	UCEC	0.091
miR-17	Hedgehog signaling pathway	0.025	-0.301	PRAD	0.167
miR-17	Hedgehog signaling pathway	0.035	-0.260	BLCA	0.167
miR-17	HIF-1 signaling pathway	0.028	-0.266	PRAD	0.111
miR-17	Hippo signaling pathway	0.042	-0.221	BLCA	0.000
miR-17	HTLV-I infection	0.048	-0.240	ESCA	0.125
miR-17	HTLV-I infection	0.000	-0.221	BLCA	0.042
miR-17	HTLV-I infection	0.000	-0.168	LUAD	0.000
miR-17	HTLV-I infection	0.009	-0.133	UCEC	0.045
miR-17	Hypertrophic cardiomyopathy (HCM)	0.001	-0.332	PRAD	0.091
miR-17	Hypertrophic cardiomyopathy (HCM)	0.048	-0.271	ESCA	0.250
miR-17	Inflammatory mediator regulation of TRP channels	0.042	-0.133	BLCA	0.222
miR-17	Insulin resistance	0.031	-0.190	BLCA	0.000
miR-17	Insulin signaling pathway	0.049	-0.194	BLCA	0.182
miR-17	Jak-STAT signaling pathway	0.047	-0.199	BLCA	0.167
miR-17	Leishmaniasis	0.002	-0.175	LUAD	0.182
miR-17	Leukocyte transendothelial migration	0.008	-0.264	BLCA	0.083
miR-17	Longevity regulating pathway - multiple species	0.014	-0.192	BLCA	0.375
miR-17	Long-term potentiation	0.005	-0.153	BLCA	0.111
miR-17	MAPK signaling pathway	0.048	-0.374	STAD	0.125
miR-17	MAPK signaling pathway	0.004	-0.251	ESCA	0.000
miR-17	MAPK signaling pathway	0.000	-0.236	BLCA	0.038
miR-17	MAPK signaling pathway	0.014	-0.157	UCEC	0.095
miR-17	Melanogenesis	0.025	-0.318	PRAD	0.111
miR-17	MicroRNAs in cancer	0.017	-0.284	PRAD	0.000
miR-17	MicroRNAs in cancer	0.000	-0.192	BLCA	0.211
miR-17	MicroRNAs in cancer	0.013	-0.139	UCEC	0.333
miR-17	Morphine addiction	0.032	-0.140	UCEC	0.200
miR-17	Osteoclast differentiation	0.000	-0.168	LUAD	0.053
miR-17	Oxytocin signaling pathway	0.001	-0.416	STAD	0.063
miR-17	Oxytocin signaling pathway	0.018	-0.328	ESCA	0.154
miR-17	Oxytocin signaling pathway	0.049	-0.320	PRAD	0.182
miR-17	Oxytocin signaling pathway	0.000	-0.223	BLCA	0.120

miR-17	Oxytocin signaling pathway	0.009	-0.153	UCEC	0.000
miR-17	Pancreatic secretion	0.007	-0.320	PRAD	0.200
miR-17	Pathways in cancer	0.003	-0.307	PRAD	0.080
miR-17	Pathways in cancer	0.000	-0.212	BLCA	0.086
miR-17	Pathways in cancer	0.004	-0.150	UCEC	0.226
miR-17	Pathways in cancer	0.019	-0.139	LUAD	0.231
miR-17	Pathways in cancer	0.017	-0.127	KIRP	0.000
miR-17	Phagosome	0.001	-0.225	LUAD	0.118
miR-17	Phospholipase D signaling pathway	0.030	-0.231	BLCA	0.250
miR-17	PI3K-Akt signaling pathway	0.000	-0.293	PRAD	0.000
miR-17	PI3K-Akt signaling pathway	0.007	-0.249	BLCA	0.042
miR-17	PI3K-Akt signaling pathway	0.000	-0.174	UCEC	0.129
miR-17	Platelet activation	0.048	-0.311	ESCA	0.100
miR-17	Platelet activation	0.000	-0.175	BLCA	0.125
miR-17	PPAR signaling pathway	0.041	-0.274	PRAD	0.286
miR-17	PPAR signaling pathway	0.046	-0.139	LUAD	0.000
miR-17	Propanoate metabolism	0.000	-0.140	LIHC	0.250
miR-17	Prostate cancer	0.027	-0.250	BLCA	0.111
miR-17	Proteoglycans in cancer	0.012	-0.449	STAD	0.125
miR-17	Proteoglycans in cancer	0.000	-0.328	PRAD	0.053
miR-17	Proteoglycans in cancer	0.004	-0.285	ESCA	0.000
miR-17	Proteoglycans in cancer	0.000	-0.222	BLCA	0.130
miR-17	Proteoglycans in cancer	0.011	-0.207	LUAD	0.000
miR-17	Proteoglycans in cancer	0.000	-0.125	UCEC	0.000
miR-17	Rap1 signaling pathway	0.034	-0.417	STAD	0.000
miR-17	Rap1 signaling pathway	0.001	-0.316	PRAD	0.000
miR-17	Rap1 signaling pathway	0.005	-0.234	BLCA	0.111
miR-17	Rap1 signaling pathway	0.023	-0.229	ESCA	0.000
miR-17	Rap1 signaling pathway	0.018	-0.152	LUAD	0.118
miR-17	Rap1 signaling pathway	0.020	-0.149	UCEC	0.167
miR-17	Ras signaling pathway	0.005	-0.317	PRAD	0.000
miR-17	Ras signaling pathway	0.035	-0.234	BLCA	0.250
miR-17	Ras signaling pathway	0.003	-0.144	UCEC	0.045
miR-17	Regulation of actin cytoskeleton	0.007	-0.345	PRAD	0.063
miR-17	Regulation of actin cytoskeleton	0.016	-0.292	ESCA	0.000
miR-17	Regulation of actin cytoskeleton	0.001	-0.273	BLCA	0.000
miR-17	Renin secretion	0.048	-0.469	STAD	0.143
miR-17	Renin secretion	0.039	-0.171	UCEC	0.250
miR-17	Renin secretion	0.042	-0.162	BLCA	0.143
miR-17	Retinol metabolism	0.028	-0.262	LIHC	0.429
miR-17	Retrograde endocannabinoid signaling	0.019	-0.161	BLCA	0.100
miR-17	Rheumatoid arthritis	0.002	-0.172	LUAD	0.417
miR-17	Salivary secretion	0.040	-0.340	PRAD	0.375

miR-17	Signaling pathways regulating pluripotency of stem cells	0.019	-0.131	LUAD	0.385
miR-17	Sphingolipid signaling pathway	0.008	-0.162	BLCA	0.000
miR-17	Staphylococcus aureus infection	0.001	-0.208	LUAD	0.100
miR-17	TGF-beta signaling pathway	0.019	-0.232	BLCA	0.111
miR-17	TGF-beta signaling pathway	0.011	-0.179	LUAD	0.200
miR-17	Th17 cell differentiation	0.008	-0.188	LUAD	0.250
miR-17	Thyroid hormone signaling pathway	0.043	-0.222	BLCA	0.200
miR-17	Thyroid hormone synthesis	0.047	-0.321	PRAD	0.429
miR-17	Tight junction	0.025	-0.512	STAD	0.000
miR-17	Tight junction	0.008	-0.174	LUAD	0.214
miR-17	TNF signaling pathway	0.005	-0.178	BLCA	0.083
miR-17	TNF signaling pathway	0.008	-0.093	LUAD	0.250
miR-17	Toxoplasmosis	0.037	-0.182	LUAD	0.182
miR-17	Transcriptional misregulation in cancer	0.050	-0.186	LUAD	0.000
miR-17	Vascular smooth muscle contraction	0.000	-0.538	STAD	0.200
miR-17	Vascular smooth muscle contraction	0.018	-0.391	ESCA	0.000
miR-17	Vascular smooth muscle contraction	0.001	-0.352	PRAD	0.231
miR-17	Vascular smooth muscle contraction	0.000	-0.265	BLCA	0.059
miR-17	Vascular smooth muscle contraction	0.000	-0.182	UCEC	0.211
miR-17	Viral myocarditis	0.005	-0.221	LUAD	0.111
miR-17	Wnt signaling pathway	0.012	-0.196	BLCA	0.000
miR-17	Wnt signaling pathway	0.039	-0.108	UCEC	0.077
miR-181c	Adipocytokine signaling pathway	0.028	-0.313	CHOL	0.444
miR-181c	Arginine and proline metabolism	0.038	-0.253	CHOL	0.429
miR-181c	Propanoate metabolism	0.007	-0.280	CHOL	0.143
miR-181c	Valine, leucine and isoleucine degradation	0.004	-0.205	CHOL	0.333
miR-199a	Adrenergic signaling in cardiomyocytes	0.026	-0.168	KICH	0.000
miR-199a	AMPK signaling pathway	0.009	-0.240	KICH	0.000
miR-199a	Calcium signaling pathway	0.009	-0.189	KICH	0.214
miR-199a	Glucagon signaling pathway	0.026	-0.198	KICH	0.222
miR-199a	HIF-1 signaling pathway	0.028	-0.112	UCEC	0.400
miR-199a	Melanoma	0.011	-0.252	KICH	0.375
miR-199a	Pathways in cancer	0.033	-0.225	KICH	0.200
miR-199a	Phospholipase D signaling pathway	0.009	-0.159	KICH	0.083
miR-199a	Pyrimidine metabolism	0.030	-0.140	LIHC	0.200
miR-199a	Rap1 signaling pathway	0.000	-0.216	KICH	0.238
miR-199a	Ras signaling pathway	0.009	-0.265	KICH	0.188
miR-199a	Vibrio cholerae infection	0.009	-0.216	KICH	0.143
miR-200a	AGE-RAGE signaling pathway in diabetic complications	0.023	-0.167	UCEC	0.250
miR-200a	Axon guidance	0.046	-0.187	BLCA	0.222
miR-200a	Calcium signaling pathway	0.025	-0.293	BLCA	0.200
miR-200a	cAMP signaling pathway	0.019	-0.253	BLCA	0.182

miR-200a	cGMP-PKG signaling pathway	0.002	-0.531	STAD	0.364
miR-200a	cGMP-PKG signaling pathway	0.000	-0.361	BLCA	0.250
miR-200a	cGMP-PKG signaling pathway	0.000	-0.173	UCEC	0.118
miR-200a	Cholinergic synapse	0.041	-0.234	BLCA	0.000
miR-200a	Circadian entrainment	0.023	-0.397	STAD	0.286
miR-200a	Dilated cardiomyopathy	0.036	-0.136	UCEC	0.000
miR-200a	Endocrine and other factor-regulated calcium reabsorption	0.025	-0.210	BLCA	0.400
miR-200a	Endocrine and other factor-regulated calcium reabsorption	0.037	-0.158	UCEC	0.400
miR-200a	FoxO signaling pathway	0.034	-0.365	BLCA	0.375
miR-200a	FoxO signaling pathway	0.012	-0.100	UCEC	0.300
miR-200a	Glucagon signaling pathway	0.034	-0.205	BLCA	0.286
miR-200a	Hippo signaling pathway	0.024	-0.145	UCEC	0.400
miR-200a	HTLV-I infection	0.001	-0.150	UCEC	0.471
miR-200a	MAPK signaling pathway	0.005	-0.337	BLCA	0.429
miR-200a	MAPK signaling pathway	0.036	-0.169	UCEC	0.154
miR-200a	MicroRNAs in cancer	0.000	-0.351	BLCA	0.286
miR-200a	MicroRNAs in cancer	0.001	-0.166	UCEC	0.231
miR-200a	Oxytocin signaling pathway	0.005	-0.436	STAD	0.000
miR-200a	Oxytocin signaling pathway	0.000	-0.239	BLCA	0.125
miR-200a	Oxytocin signaling pathway	0.004	-0.155	UCEC	0.000
miR-200a	Pathways in cancer	0.002	-0.287	BLCA	0.211
miR-200a	Pathways in cancer	0.025	-0.133	UCEC	0.056
miR-200a	PI3K-Akt signaling pathway	0.009	-0.351	BLCA	0.188
miR-200a	PI3K-Akt signaling pathway	0.030	-0.144	UCEC	0.063
miR-200a	Platelet activation	0.002	-0.247	BLCA	0.100
miR-200a	Proteoglycans in cancer	0.019	-0.263	BLCA	0.091
miR-200a	Proteoglycans in cancer	0.022	-0.137	UCEC	0.083
miR-200a	Rap1 signaling pathway	0.012	-0.147	UCEC	0.462
miR-200a	Ras signaling pathway	0.034	-0.276	BLCA	0.364
miR-200a	Ras signaling pathway	0.003	-0.122	UCEC	0.200
miR-200a	Regulation of actin cytoskeleton	0.001	-0.367	BLCA	0.143
miR-200a	Regulation of actin cytoskeleton	0.013	-0.130	UCEC	0.385
miR-200a	Renin secretion	0.002	-0.174	UCEC	0.250
miR-200a	Serotonergic synapse	0.049	-0.344	STAD	0.143
miR-200a	Sphingolipid signaling pathway	0.025	-0.167	BLCA	0.250
miR-200a	Thyroid hormone synthesis	0.031	-0.207	BLCA	0.000
miR-200a	Vascular smooth muscle contraction	0.001	-0.550	STAD	0.200
miR-200a	Vascular smooth muscle contraction	0.000	-0.259	BLCA	0.167
miR-200a	Vascular smooth muscle contraction	0.000	-0.173	UCEC	0.133
miR-20a	Adherens junction	0.001	-0.134	UCEC	0.417
miR-20a	Adrenergic signaling in cardiomyocytes	0.021	-0.340	PRAD	0.091
miR-20a	Aldosterone synthesis and secretion	0.013	-0.207	LUSC	0.308

miR-20a	Aldosterone synthesis and secretion	0.013	-0.123	UCEC	0.100
miR-20a	Aldosterone-regulated sodium reabsorption	0.001	-0.312	PRAD	0.143
miR-20a	Amoebiasis	0.046	-0.143	CHOL	0.455
miR-20a	Axon guidance	0.001	-0.305	PRAD	0.125
miR-20a	cGMP-PKG signaling pathway	0.000	-0.361	PRAD	0.235
miR-20a	cGMP-PKG signaling pathway	0.002	-0.269	ESCA	0.375
miR-20a	cGMP-PKG signaling pathway	0.022	-0.257	LUSC	0.263
miR-20a	Cholinergic synapse	0.012	-0.139	UCEC	0.333
miR-20a	Circadian entrainment	0.005	-0.160	UCEC	0.167
miR-20a	Cytokine-cytokine receptor interaction	0.017	-0.266	LUSC	0.333
miR-20a	ECM-receptor interaction	0.002	-0.175	CHOL	0.231
miR-20a	Endocrine and other factor-regulated calcium reabsorption	0.018	-0.333	PRAD	0.167
miR-20a	FoxO signaling pathway	0.001	-0.186	UCEC	0.313
miR-20a	Gap junction	0.007	-0.137	UCEC	0.364
miR-20a	Gastric acid secretion	0.012	-0.331	PRAD	0.250
miR-20a	Hedgehog signaling pathway	0.018	-0.298	PRAD	0.167
miR-20a	Hematopoietic cell lineage	0.017	-0.295	LUSC	0.071
miR-20a	Hippo signaling pathway	0.021	-0.205	LUSC	0.056
miR-20a	HTLV-I infection	0.021	-0.228	LUSC	0.154
miR-20a	HTLV-I infection	0.000	-0.127	UCEC	0.240
miR-20a	Insulin secretion	0.021	-0.324	PRAD	0.125
miR-20a	Leishmaniasis	0.002	-0.234	LUSC	0.143
miR-20a	MAPK signaling pathway	0.002	-0.211	ESCA	0.250
miR-20a	MAPK signaling pathway	0.004	-0.146	UCEC	0.273
miR-20a	Melanogenesis	0.018	-0.338	PRAD	0.222
miR-20a	Melanoma	0.044	-0.153	CHOL	0.333
miR-20a	Osteoclast differentiation	0.000	-0.254	LUSC	0.045
miR-20a	Oxytocin signaling pathway	0.005	-0.150	UCEC	0.375
miR-20a	Pancreatic secretion	0.013	-0.345	PRAD	0.222
miR-20a	Phagosome	0.021	-0.294	LUSC	0.167
miR-20a	Platelet activation	0.025	-0.232	ESCA	0.091
miR-20a	Platelet activation	0.026	-0.156	UCEC	0.417
miR-20a	Prostate cancer	0.023	-0.165	CHOL	0.364
miR-20a	Protein digestion and absorption	0.023	-0.151	CHOL	0.455
miR-20a	Ras signaling pathway	0.005	-0.327	PRAD	0.063
miR-20a	Ras signaling pathway	0.006	-0.141	UCEC	0.350
miR-20a	Regulation of actin cytoskeleton	0.001	-0.364	PRAD	0.176
miR-20a	Regulation of actin cytoskeleton	0.017	-0.228	ESCA	0.313
miR-20a	Renin secretion	0.010	-0.157	UCEC	0.444
miR-20a	Rheumatoid arthritis	0.036	-0.243	LUSC	0.000
miR-20a	Salivary secretion	0.027	-0.351	PRAD	0.125
miR-20a	Small cell lung cancer	0.010	-0.182	CHOL	0.250

miR-20a	Staphylococcus aureus infection	0.007	-0.287	LUSC	0.182
miR-20a	TGF-beta signaling pathway	0.023	-0.259	LUSC	0.250
miR-20a	Thyroid hormone signaling pathway	0.039	-0.345	PRAD	0.111
miR-20a	Thyroid hormone signaling pathway	0.048	-0.213	ESCA	0.200
miR-20a	Thyroid hormone synthesis	0.035	-0.322	PRAD	0.286
miR-20a	Tight junction	0.002	-0.135	LUSC	0.200
miR-20a	Vascular smooth muscle contraction	0.002	-0.362	PRAD	0.250
miR-20a	Vascular smooth muscle contraction	0.017	-0.271	LUSC	0.125
miR-20a	Vascular smooth muscle contraction	0.000	-0.171	UCEC	0.450
miR-21	AGE-RAGE signaling pathway in diabetic complications	0.047	-0.130	UCEC	0.000
miR-21	AMPK signaling pathway	0.002	-0.136	CHOL	0.462
miR-21	Focal adhesion	0.016	-0.157	BLCA	0.222
miR-21	Glucagon signaling pathway	0.032	-0.190	CHOL	0.333
miR-21	Glycerolipid metabolism	0.026	-0.233	CHOL	0.143
miR-21	Hedgehog signaling pathway	0.001	-0.118	UCEC	0.000
miR-21	Pathways in cancer	0.011	-0.141	BLCA	0.214
miR-21	Progesterone-mediated oocyte maturation	0.018	-0.137	BLCA	0.000
miR-21	Propanoate metabolism	0.018	-0.364	KIRC	0.400
miR-21	Proteoglycans in cancer	0.047	-0.125	UCEC	0.300
miR-21	Valine, leucine and isoleucine degradation	0.000	-0.358	KIRC	0.125
miR-27b	Amoebiasis	0.047	-0.158	KIRC	0.200
miR-27b	Cell adhesion molecules (CAMs)	0.001	-0.149	KIRC	0.125
miR-27b	Rap1 signaling pathway	0.044	-0.106	KIRC	0.250
miR-27b	Staphylococcus aureus infection	0.004	-0.160	KIRC	0.000
miR-28	Cell cycle	0.000	-0.233	STAD	0.077
miR-28	Fanconi anemia pathway	0.006	-0.249	STAD	0.333
miR-29c	ECM-receptor interaction	0.003	-0.117	LUSC	0.400
miR-30a	Breast cancer	0.038	-0.107	LUSC	0.267
miR-30a	Cell cycle	0.000	-0.372	STAD	0.211
miR-30a	Cell cycle	0.000	-0.336	LUAD	0.125
miR-30a	Cell cycle	0.000	-0.183	LUSC	0.240
miR-30a	Cell cycle	0.000	-0.126	ESCA	0.350
miR-30a	Cysteine and methionine metabolism	0.035	-0.190	LUAD	0.000
miR-30a	Cysteine and methionine metabolism	0.002	-0.135	LUSC	0.100
miR-30a	Cytokine-cytokine receptor interaction	0.005	-0.163	KIRC	0.273
miR-30a	Glutathione metabolism	0.015	-0.171	LUSC	0.222
miR-30a	Hippo signaling pathway	0.029	-0.141	LUSC	0.313
miR-30a	Homologous recombination	0.038	-0.126	LUSC	0.143
miR-30a	MicroRNAs in cancer	0.035	-0.274	LUAD	0.182
miR-30a	MicroRNAs in cancer	0.000	-0.237	STAD	0.333
miR-30a	MicroRNAs in cancer	0.019	-0.135	ESCA	0.200
miR-30a	MicroRNAs in cancer	0.000	-0.133	LUSC	0.048

miR-30a	Natural killer cell mediated cytotoxicity	0.031	-0.151	KIRC	0.083
miR-30a	Oocyte meiosis	0.022	-0.393	STAD	0.375
miR-30a	Oocyte meiosis	0.019	-0.144	ESCA	0.444
miR-30a	p53 signaling pathway	0.007	-0.327	STAD	0.286
miR-30a	p53 signaling pathway	0.002	-0.168	LUSC	0.167
miR-30a	p53 signaling pathway	0.000	-0.127	ESCA	0.300
miR-30a	Pathways in cancer	0.015	-0.109	LUSC	0.094
miR-30a	Phagosome	0.017	-0.219	KIRC	0.429
miR-30a	Pyrimidine metabolism	0.039	-0.172	LUSC	0.167
miR-30a	Rap1 signaling pathway	0.010	-0.154	KIRC	0.278
miR-30a	Ribosome biogenesis in eukaryotes	0.003	-0.147	LUSC	0.231
miR-30a	RNA transport	0.012	-0.147	LUSC	0.333
miR-30a	Small cell lung cancer	0.014	-0.303	STAD	0.143
miR-30a	Small cell lung cancer	0.030	-0.144	LUSC	0.182
miR-30a	Tuberculosis	0.039	-0.130	KIRC	0.429
miR-30a	Viral carcinogenesis	0.019	-0.150	ESCA	0.250
miR-378a	Biosynthesis of amino acids	0.010	-0.132	LUAD	0.143
miR-378a	Endocytosis	0.041	-0.168	CHOL	0.150
miR-378a	Pathways in cancer	0.004	-0.235	CHOL	0.000
miR-378a	Small cell lung cancer	0.041	-0.253	CHOL	0.300
miR-378a	Transcriptional misregulation in cancer	0.004	-0.196	CHOL	0.000
miR-425	Calcium signaling pathway	0.033	-0.218	BLCA	0.125
miR-425	Focal adhesion	0.001	-0.260	BLCA	0.154
miR-425	Focal adhesion	0.002	-0.165	UCEC	0.071
miR-425	FoxO signaling pathway	0.005	-0.221	BLCA	0.333
miR-425	MicroRNAs in cancer	0.018	-0.250	BLCA	0.250
miR-425	Oxytocin signaling pathway	0.005	-0.155	BLCA	0.200
miR-425	Phospholipase D signaling pathway	0.033	-0.229	BLCA	0.429
miR-425	PI3K-Akt signaling pathway	0.007	-0.177	UCEC	0.294
miR-425	Rap1 signaling pathway	0.012	-0.219	BLCA	0.400
miR-425	Vascular smooth muscle contraction	0.019	-0.240	BLCA	0.143
miR-708	Adrenergic signaling in cardiomyocytes	0.021	-0.108	BLCA	0.375
miR-708	Arrhythmogenic right ventricular cardiomyopathy (ARVC)	0.004	-0.166	ESCA	0.143
miR-708	cAMP signaling pathway	0.033	-0.105	BLCA	0.222
miR-708	Dilated cardiomyopathy	0.000	-0.215	ESCA	0.333
miR-708	Hematopoietic cell lineage	0.029	-0.204	LUSC	0.333
miR-708	Hypertrophic cardiomyopathy (HCM)	0.000	-0.197	ESCA	0.333
miR-708	MAPK signaling pathway	0.000	-0.094	BLCA	0.250
miR-708	Oxytocin signaling pathway	0.000	-0.091	BLCA	0.467
miR-708	Proteoglycans in cancer	0.015	-0.095	BLCA	0.200
miR-708	Rap1 signaling pathway	0.029	-0.208	LUSC	0.421
miR-708	Valine, leucine and isoleucine degradation	0.002	-0.179	KIRC	0.000

miR-93	Adherens junction	0.045	-0.277	LUSC	0.182
miR-93	Adherens junction	0.000	-0.150	UCEC	0.385
miR-93	Adrenergic signaling in cardiomyocytes	0.034	-0.216	BLCA	0.455
miR-93	Adrenergic signaling in cardiomyocytes	0.020	-0.163	ESCA	0.083
miR-93	AGE-RAGE signaling pathway in diabetic complications	0.004	-0.249	BLCA	0.273
miR-93	AGE-RAGE signaling pathway in diabetic complications	0.033	-0.156	KIRP	0.444
miR-93	Aldosterone synthesis and secretion	0.023	-0.193	KIRP	0.333
miR-93	Aldosterone-regulated sodium reabsorption	0.002	-0.366	PRAD	0.143
miR-93	Arrhythmogenic right ventricular cardiomyopathy (ARVC)	0.000	-0.433	PRAD	0.000
miR-93	Axon guidance	0.001	-0.226	BLCA	0.176
miR-93	Bladder cancer	0.043	-0.318	BLCA	0.000
miR-93	Butanoate metabolism	0.018	-0.191	LIHC	0.200
miR-93	Calcium signaling pathway	0.014	-0.285	BLCA	0.143
miR-93	cAMP signaling pathway	0.006	-0.261	BLCA	0.188
miR-93	cAMP signaling pathway	0.024	-0.157	ESCA	0.000
miR-93	Carbon metabolism	0.018	-0.233	LIHC	0.300
miR-93	cGMP-PKG signaling pathway	0.000	-0.482	STAD	0.263
miR-93	cGMP-PKG signaling pathway	0.000	-0.421	PRAD	0.158
miR-93	cGMP-PKG signaling pathway	0.000	-0.312	BLCA	0.200
miR-93	cGMP-PKG signaling pathway	0.001	-0.230	ESCA	0.235
miR-93	cGMP-PKG signaling pathway	0.000	-0.171	UCEC	0.440
miR-93	Chemokine signaling pathway	0.015	-0.278	BLCA	0.286
miR-93	Cholinergic synapse	0.007	-0.215	BLCA	0.455
miR-93	Chronic myeloid leukemia	0.038	-0.215	BLCA	0.000
miR-93	Circadian entrainment	0.000	-0.365	STAD	0.231
miR-93	Circadian entrainment	0.008	-0.253	BLCA	0.300
miR-93	Circadian rhythm	0.005	-0.280	BLCA	0.167
miR-93	Colorectal cancer	0.021	-0.218	BLCA	0.429
miR-93	Complement and coagulation cascades	0.006	-0.291	LIHC	0.222
miR-93	Cytokine-cytokine receptor interaction	0.045	-0.320	LUSC	0.231
miR-93	Dilated cardiomyopathy	0.002	-0.424	PRAD	0.000
miR-93	Dilated cardiomyopathy	0.020	-0.221	ESCA	0.000
miR-93	ECM-receptor interaction	0.017	-0.393	PRAD	0.250
miR-93	EGFR tyrosine kinase inhibitor resistance	0.004	-0.399	PRAD	0.111
miR-93	Endocrine and other factor-regulated calcium reabsorption	0.004	-0.397	PRAD	0.000
miR-93	Endocrine resistance	0.009	-0.230	BLCA	0.400
miR-93	ErbB signaling pathway	0.014	-0.177	BLCA	0.000
miR-93	Fatty acid degradation	0.005	-0.220	LIHC	0.429
miR-93	Focal adhesion	0.000	-0.409	PRAD	0.045
miR-93	Focal adhesion	0.000	-0.324	BLCA	0.000
miR-93	Focal adhesion	0.017	-0.204	ESCA	0.133

miR-93	Focal adhesion	0.000	-0.189	UCEC	0.240
miR-93	Focal adhesion	0.038	-0.166	KIRP	0.385
miR-93	FoxO signaling pathway	0.001	-0.317	BLCA	0.357
miR-93	FoxO signaling pathway	0.001	-0.218	UCEC	0.250
miR-93	FoxO signaling pathway	0.039	-0.119	CHOL	0.400
miR-93	Gap junction	0.014	-0.279	BLCA	0.333
miR-93	Gap junction	0.007	-0.158	UCEC	0.364
miR-93	Gastric acid secretion	0.011	-0.412	PRAD	0.375
miR-93	Glucagon signaling pathway	0.026	-0.215	BLCA	0.444
miR-93	Hedgehog signaling pathway	0.022	-0.245	BLCA	0.000
miR-93	Hematopoietic cell lineage	0.045	-0.378	LUSC	0.077
miR-93	Hippo signaling pathway	0.021	-0.284	BLCA	0.167
miR-93	HTLV-I infection	0.000	-0.235	BLCA	0.280
miR-93	HTLV-I infection	0.001	-0.150	UCEC	0.458
miR-93	Hypertrophic cardiomyopathy (HCM)	0.002	-0.424	PRAD	0.000
miR-93	Hypertrophic cardiomyopathy (HCM)	0.032	-0.232	ESCA	0.000
miR-93	Insulin resistance	0.016	-0.221	BLCA	0.100
miR-93	Insulin secretion	0.021	-0.398	PRAD	0.250
miR-93	Insulin signaling pathway	0.025	-0.207	BLCA	0.091
miR-93	Jak-STAT signaling pathway	0.012	-0.261	BLCA	0.077
miR-93	Leishmaniasis	0.045	-0.356	LUSC	0.455
miR-93	Leukocyte transendothelial migration	0.004	-0.305	BLCA	0.083
miR-93	Long-term potentiation	0.026	-0.184	BLCA	0.143
miR-93	MAPK signaling pathway	0.010	-0.173	ESCA	0.278
miR-93	MAPK signaling pathway	0.007	-0.160	UCEC	0.476
miR-93	Melanoma	0.034	-0.284	BLCA	0.429
miR-93	MicroRNAs in cancer	0.009	-0.357	PRAD	0.083
miR-93	MicroRNAs in cancer	0.000	-0.293	BLCA	0.000
miR-93	MicroRNAs in cancer	0.020	-0.223	ESCA	0.083
miR-93	MicroRNAs in cancer	0.001	-0.168	UCEC	0.294
miR-93	Osteoclast differentiation	0.000	-0.362	LUSC	0.045
miR-93	Osteoclast differentiation	0.038	-0.243	BLCA	0.400
miR-93	Osteoclast differentiation	0.036	-0.142	UCEC	0.417
miR-93	Oxytocin signaling pathway	0.000	-0.381	STAD	0.313
miR-93	Oxytocin signaling pathway	0.000	-0.269	BLCA	0.190
miR-93	Oxytocin signaling pathway	0.027	-0.229	ESCA	0.000
miR-93	Oxytocin signaling pathway	0.011	-0.170	UCEC	0.400
miR-93	Pancreatic secretion	0.012	-0.405	PRAD	0.333
miR-93	Pathogenic Escherichia coli infection	0.036	-0.197	BLCA	0.167
miR-93	Pathways in cancer	0.000	-0.373	PRAD	0.077
miR-93	Pathways in cancer	0.000	-0.258	BLCA	0.147
miR-93	Pathways in cancer	0.044	-0.206	BRCA	0.261
miR-93	Pathways in cancer	0.002	-0.173	UCEC	0.333

miR-93	Pathways in cancer	0.023	-0.143	KIRP	0.273
miR-93	Peroxisome	0.028	-0.211	LIHC	0.375
miR-93	Pertussis	0.050	-0.325	LUSC	0.455
miR-93	Phospholipase D signaling pathway	0.028	-0.290	BLCA	0.364
miR-93	PI3K-Akt signaling pathway	0.000	-0.346	PRAD	0.080
miR-93	PI3K-Akt signaling pathway	0.001	-0.309	BLCA	0.160
miR-93	PI3K-Akt signaling pathway	0.001	-0.183	UCEC	0.207
miR-93	Platelet activation	0.002	-0.237	BLCA	0.231
miR-93	Platelet activation	0.032	-0.179	ESCA	0.100
miR-93	Prolactin signaling pathway	0.036	-0.236	BLCA	0.143
miR-93	Propanoate metabolism	0.001	-0.215	LIHC	0.429
miR-93	Prostate cancer	0.015	-0.275	BLCA	0.444
miR-93	Proteoglycans in cancer	0.002	-0.425	PRAD	0.125
miR-93	Proteoglycans in cancer	0.000	-0.247	BLCA	0.095
miR-93	Proteoglycans in cancer	0.008	-0.174	ESCA	0.188
miR-93	Proteoglycans in cancer	0.016	-0.159	UCEC	0.294
miR-93	Rap1 signaling pathway	0.002	-0.390	PRAD	0.313
miR-93	Rap1 signaling pathway	0.017	-0.297	BLCA	0.400
miR-93	Rap1 signaling pathway	0.000	-0.289	LUSC	0.233
miR-93	Rap1 signaling pathway	0.041	-0.138	KIRP	0.308
miR-93	Ras signaling pathway	0.012	-0.385	PRAD	0.200
miR-93	Ras signaling pathway	0.015	-0.280	BLCA	0.313
miR-93	Ras signaling pathway	0.023	-0.158	UCEC	0.278
miR-93	Regulation of actin cytoskeleton	0.000	-0.418	PRAD	0.150
miR-93	Regulation of actin cytoskeleton	0.000	-0.339	BLCA	0.095
miR-93	Regulation of actin cytoskeleton	0.008	-0.209	ESCA	0.000
miR-93	Renin secretion	0.030	-0.165	UCEC	0.375
miR-93	Salivary secretion	0.029	-0.417	PRAD	0.375
miR-93	Signaling pathways regulating pluripotency of stem cells	0.026	-0.186	BLCA	0.091
miR-93	Sphingolipid signaling pathway	0.012	-0.220	BLCA	0.182
miR-93	Staphylococcus aureus infection	0.030	-0.390	LUSC	0.400
miR-93	TGF-beta signaling pathway	0.026	-0.247	BLCA	0.250
miR-93	Thyroid hormone signaling pathway	0.024	-0.244	BLCA	0.300
miR-93	Thyroid hormone signaling pathway	0.027	-0.173	ESCA	0.100
miR-93	Thyroid hormone signaling pathway	0.023	-0.166	KIRP	0.273
miR-93	Thyroid hormone synthesis	0.037	-0.381	PRAD	0.286
miR-93	Tight junction	0.023	-0.500	STAD	0.167
miR-93	Tight junction	0.024	-0.311	BLCA	0.091
miR-93	Tight junction	0.001	-0.200	LUSC	0.143
miR-93	TNF signaling pathway	0.016	-0.198	BLCA	0.400
miR-93	Transcriptional misregulation in cancer	0.047	-0.301	BLCA	0.083
miR-93	Tryptophan metabolism	0.001	-0.225	LIHC	0.375

miR-93	Tyrosine metabolism	0.008	-0.289	LIHC	0.333
miR-93	Valine, leucine and isoleucine degradation	0.000	-0.227	LIHC	0.455
miR-93	Vascular smooth muscle contraction	0.000	-0.506	STAD	0.125
miR-93	Vascular smooth muscle contraction	0.001	-0.443	PRAD	0.154
miR-93	Vascular smooth muscle contraction	0.000	-0.340	BLCA	0.133
miR-93	Vascular smooth muscle contraction	0.030	-0.309	LUSC	0.125
miR-93	Vascular smooth muscle contraction	0.017	-0.275	ESCA	0.091
miR-93	Vascular smooth muscle contraction	0.000	-0.196	UCEC	0.278
miR-93	Vascular smooth muscle contraction	0.033	-0.177	KIRP	0.300
miR-93	Wnt signaling pathway	0.046	-0.408	PRAD	0.100
miR-93	Wnt signaling pathway	0.006	-0.242	BLCA	0.154

$^{\$}$FDR (false discovery rate) adjusted *P*-value (hypergeometric test). $^{\#}$Score: Coordinated regulation strength. A lower score corresponds to 5p/3p pair mediated greater coordinated regulation of pathway genes.

Supplementary Table 7. Top 20 significantly enriched gene sets in the down-regulated genes that positively or negatively regulate the biological processes listed

Gene Ontology Biological Processes	*P*-value[$]	FDR[#]
Positive regulation of cell migration	1.068E-07	7.023E-05
Positive regulation of developmental process	1.523E-07	9.303E-05
Positive regulation of cell motility	1.797E-07	1.024E-04
Positive regulation of cellular component movement	2.570E-07	1.292E-04
Positive regulation of locomotion	4.457E-07	1.814E-04
Positive regulation of multicellular organismal process	4.692E-07	1.823E-04
Positive regulation of cell differentiation	9.159E-07	2.900E-04
Positive regulation of epithelial cell migration	1.199E-06	3.179E-04
Negative regulation of cell communication	1.776E-06	3.893E-04
Negative regulation of signaling	1.906E-06	4.073E-04
Positive regulation of protein tyrosine kinase activity	2.105E-06	4.389E-04
Negative regulation of response to stimulus	3.957E-06	7.197E-04
Positive regulation of cellular component organization	4.391E-06	7.820E-04
Negative regulation of signal transduction	8.959E-06	1.445E-03
Positive regulation of cell proliferation	1.015E-05	1.550E-03
Positive regulation of signal transduction	2.733E-05	3.157E-03
Positive regulation of nucleobase-containing compound metabolic process	3.180E-05	3.562E-03
Positive regulation of response to stimulus	4.047E-05	4.219E-03
Positive regulation of endothelial cell migration	5.968E-05	5.669E-03
Positive regulation of response to external stimulus	7.116E-05	6.542E-03

[#]FDR (false discovery rate) adjusted [$]*P*-value (hypergeometric test).

Supplementary Table 8. Top 20* significantly enriched gene sets in the up-regulated genes that positively or negatively regulate the biological processes listed

Gene Ontology Biological Processes	*P*-value$	FDR#
Positive regulation of cell communication	1.197E-04	2.047E-02
Positive regulation of multicellular organismal process	3.080E-04	3.872E-02
Positive regulation of response to stimulus	1.179E-04	2.047E-02
Positive regulation of signaling	1.397E-04	2.154E-02
Negative regulation of cell communication	2.259E-04	2.926E-02
Negative regulation of cell migration	3.407E-04	3.972E-02
Negative regulation of cellular component movement	3.438E-04	3.972E-02
Negative regulation of cellular response to growth factor stimulus	2.152E-04	2.831E-02
Negative regulation of multicellular organismal process	1.396E-04	2.154E-02
Negative regulation of proteolysis	3.380E-04	3.972E-02
Negative regulation of response to stimulus	1.500E-04	2.182E-02
Negative regulation of signaling	1.346E-04	2.154E-02
Negative regulation of transmembrane receptor protein serine/threonine kinase signaling pathway	1.506E-04	2.182E-02

*Only 13 pathways showed significant (FDR<0.05) enrichment. #FDR (false discovery rate) adjusted $*P*-value (hypergeometric test).

Supplementary Table 9. Overall survival association (z-score) of single strand, double strand, and signature (four strands) miRNA miR-30a and miR-145

miRNA	Z-score$	Cancer type
Signature miRNA	-1.814	BRCA
Signature miRNA	-2.183	CHOL
Signature miRNA	-3.184	KIRC
Signature miRNA	-2.489	LUAD
Signature miRNA	-2.734	LUSC
miR-30a 5p/3p	-1.33	BRCA
miR-30a 5p/3p	-1.143	CHOL
miR-30a 5p/3p	-1.007	KIRC
miR-30a 5p/3p	-1.197	LUAD
miR-30a 5p/3p	-2.18	LUSC
miR-30a-5p	-1.669	BRCA
miR-30a-5p	-1.889	CHOL
miR-30a-5p	-0.608	KIRC
miR-30a-5p	-1.602	LUAD
miR-30a-5p	-1.617	LUSC
miR-30a-3p	-1.597	BRCA
miR-30a-3p	-0.784	CHOL
miR-30a-3p	-2.553	KIRC
miR-30a-3p	-0.919	LUAD
miR-30a-3p	-1.039	LUSC
miR-145 5p/3p	0.479	BRCA
miR-145 5p/3p	-2.035	CHOL
miR-145 5p/3p	-2.009	KIRC
miR-145 5p/3p	-2.566	LUAD
miR-145 5p/3p	-1.514	LUSC
miR-145-5p	-0.141	BRCA
miR-145-5p	-1.795	CHOL
miR-145-5p	-1.08	KIRC
miR-145-5p	-2.304	LUAD
miR-145-5p	-1.428	LUSC
miR-145-3p	1.262	BRCA
miR-145-3p	-1.884	CHOL
miR-145-3p	-1.486	KIRC
miR-145-3p	-2.218	LUAD
miR-145-3p	-0.702	LUSC

$Lower the z-score indicates the greater association between miRNA down-regulation and reduced overall survival

References

1. Benjamini Y, Hochberg Y. Controlling the False Discovery Rate - a Practical and Powerful Approach to Multiple Testing. *Journal of the Royal Statistical Society Series B-Methodological* **57**, 289-300 (1995).
2. Yang X, *et al.* Both mature miR-17-5p and passenger strand miR-17-3p target TIMP3 and induce prostate tumor growth and invasion. *Nucleic Acids Res* **41**, 9688-9704 (2013).
3. Westermarck J, Kahari VM. Regulation of matrix metalloproteinase expression in tumor invasion. *FASEB J* **13**, 781-792 (1999).
4. Page-McCaw A, Ewald AJ, Werb Z. Matrix metalloproteinases and the regulation of tissue remodelling. *Nat Rev Mol Cell Biol* **8**, 221-233 (2007).
